# Automated radiosynthesis and preclinical evaluation of two new PSMA-617 derivatives radiolabelled via [^18^F]AlF^2+^ method

**DOI:** 10.1186/s41181-024-00280-0

**Published:** 2024-06-21

**Authors:** Marco Nicola Iannone, Silvia Valtorta, Stefano Stucchi, Stefano Altomonte, Elia Anna Turolla, Elisa Vino, Paolo Rainone, Valentina Zecca, Alessia Lo Dico, Marco Maspero, Mariangela Figini, Matteo Bellone, Samuele Ciceri, Diego Colombo, Clizia Chinello, Lisa Pagani, Rosa Maria Moresco, Sergio Todde, Patrizia Ferraboschi

**Affiliations:** 1 Tecnomed Foundation, University of Milano-Bicocca, Monza, Italy; 2 Institute of Molecular Bioimaging and Physiology (IBFM), National Research Council (CNR), Segrate, Italy; 3 NBFC, National Biodiversity Future Center, Palermo, Italy; 4 Department of Nuclear Medicine, San Raffaele Scientific Institute, IRCCS, Milan, Italy; 5 School of Medicine and Surgery, University of Milano-Bicocca, Monza, Italy; 6 ANP2, Department of Advanced Diagnostics, Fondazione IRCCS, Istituto Nazionale Dei Tumori, Milan, Italy; 7 Division of Immunology, Transplantation and Infectious Diseases, San Raffaele Scientific Institute, IRCCS, Milan, Italy; 8 Department of Medical Biotechnology and Translational Medicine, University of Milano, Milan, Italy

**Keywords:** PSMA-617, NODA, RESCA, [^18^F]AlF^2+^, Fluorine-18, Automation, Prostate cancer, Glioma

## Abstract

**Background:**

In the last decade the development of new PSMA-ligand based radiopharmaceuticals for the imaging and therapy of prostate cancer has been a highly active and important area of research. The most promising derivative in terms of interaction with the antigen and clinical properties has been found to be “PSMA-617”, and its lutetium-177 radiolabelled version has recently been approved by EU and USA regulatory agencies for therapeutic purposes. For the above reasons, the development of new derivatives of PSMA-617 radiolabelled with fluorine-18 may still be of great interest. This paper proposes the comparison of two different PSMA-617 derivatives functionalized with NODA and RESCA chelators, respectively, radiolabelled via [^18^F]AlF^2+^ complexation.

**Results:**

The organic synthesis of two PSMA-617 derivatives and their radiolabelling via [^18^F]AlF^2+^ complexation resulted to proceed efficiently and successfully. Moreover, stability in solution and in plasma has been evaluated. The whole radiosynthesis procedure has been fully automated, and the final products have been obtained with radiochemical yield and purity potentially suitable for clinical studies. The biodistribution of the two derivatives was performed both in prostate cancer and glioma tumour models. Compared with the reference [^18^F]F-PSMA-1007 and [^18^F]F-PSMA-617-RESCA, [^18^F]F-PSMA-617-NODA derivative showed a higher uptake in both tumors, faster clearance in non-target organs, and lower uptake in salivary glands.

**Conclusion:**

PSMA-617 NODA and RESCA derivatives were radiolabelled successfully via [^18^F]AlF^2+^ chelation, the former being more stable in solution and human plasma. Moreover, preclinical biodistribution studies showed that [^18^F]F-PSMA-617-NODA might be of potential interest for clinical applications.

**Supplementary Information:**

The online version contains supplementary material available at 10.1186/s41181-024-00280-0.

## Introduction

Positron emission tomography (PET) is an imaging technique which may have a significant impact in oncology, due to its sensitiveness, non-invasiveness and high-quality images. Nowadays, several increasingly selective and specific radiopharmaceuticals are available and routinely used for the diagnosis of a wide variety of pathological conditions, among which prostate cancer (PCa), the second most common type of diagnosed malignancy in men worldwide, is of great relevance (Duclos et al. 2021; Kawad et al. 2022). For the above reasons, since the’80 s PET researchers have focused their attention on suitable radiopharmaceuticals, with the development of [^11^C]choline, followed by other “metabolic” radiotracers such as [^18^F]fluoromethyl (and fluoroethyl) choline, [^18^F]fluciclovine and others (Welle et al. 2016).

In recent years, the prostate-specific membrane antigen (PSMA), was identified as a highly attractive target. It shows a significant overexpression in prostatic cancerous cells and upregulation in poorly differentiated, metastatic and hormone refractory carcinomas (Bednarova et al. 2017). Thus, a wide variety of PSMA-ligands, based on direct ligand–protein interaction, were developed and investigated. Receptor-specific molecules like [^18^F]F-DCFBC, [^18^F]F-DCPyL, [^18^F]F-PSMA-1007 and [^68^Ga]Ga-PSMA-11 were studied and resulted to be superior, compared with “metabolic” radiotracers in terms of biodistribution, accuracy and specificity. Indeed, PSMA-based ligands are now considered as the “gold standard” in the imaging of PCa (Barinka et al. 2008; Chen et al. 2011; Eder et al. 2012; Hillier et al. 2013; Benesova et al. 2015; Cardinale et al. 2017a), and also their therapeutic application are ever growing, with the recent approval by FDA and EMA of the radiopharmaceutical “Pluvicto” ([^177^Lu]Lu-PSMA-617) (Keam 2022). [^18^F]F-PSMA-1007 is currently one of the most frequently used PET radiopharmaceuticals, at least in the European Union (EU), because it may be prepared with high yield and purity using commercially available kit reagents and cassettes suitable for different automated synthesis platforms, exploiting the more favorable radiophysical characteristics of fluorine-18 over gallium-68 in terms of half-life, costs and amount of available radionuclide. Moreover, a specific European Pharmacopoeia monograph allows its preparation for routine clinical use as an officinal galenic preparation in several EU countries (Giesel et al. 2017; Cardinale et al. 2017b; PSMA-1007 (18F) Injection; European Medicines Agency 2024).

Among the various PSMA derivatives, in 2015 was first reported the synthesis of PSMA-617, which was originally designed in view of its possible use as a therapeutic agent (Benešová et al. 2016); indeed, it was then radiolabelled with different radioisotopes, mostly gallium-68 for imaging purposes and lutetium-177 and actinium-225 for therapy (Ruigrok et al. 2019). PSMA-617 was designed with a modified linker, which improved its interaction with S1-accessory hydrophobic pocket in protein binding site (Davis et al. 2005; Zhang et al. 2010); this change, in turn, resulted in an improved pharmacokinetic profile and a reduced radiation dose to healthy tissues such as the salivary glands, which is a positive feature both in imaging and therapy (Heynickx et al. 2021a).

In addition, it has been recently demonstrated that PSMA is also expressed in the tumour-associated neo-vasculature of high-grade glioma (HGG) and might interact with different pathways promoting angiogenesis (Matsuda et al. 2018; Truckenmueller et al. 2022). At the moment, only a few studies evaluating PSMA targeted imaging of HGG in clinical practice have been performed, but they could confirm that HGG/glioblastoma are PSMA-avid compared to low-grade tumours (Valtorta et al. 2020).

Fluorine-18 can be introduced into macromolecules by means of several methods, the most frequent of which is via nucleophilic substitution on highly reactive leaving groups. This method occurs in “harsh” reaction conditions, often requiring temperature > 80 °C, and can’t be sustainable for fluorine-18 introduction into biologically active macromolecules, due to their inherent structural thermal sensitivity (Ajenjo et al. 2021).

In order to overcome this limitation, several strategies were designed, one of the most popular of which is the so called “click” chemistry. This approach comprises a set of reactions which have several advantages: they are fast, regioselective, easily give pure products with excellent yields and, which is most important in this context, they are carried out under *mild* conditions in aqueous media (Kolb et al. 2001; Ahmad Fuaad et al. 2013; Aragao-Leoneti et al. 2010).

Other radiosynthetic routes include the formation of strong bonds between fluorine-18 and a Lewis acid. Silicon and boron are the most used and a wide variety of biologically active molecules were radiolabelled with fluorine-18 using such a radiochemistry approach (Schirrmacher et al. 2006; Ting et al. 2005; Bernard-Gauthier et al. 2016). Moreover, recently new hybrid PSMA radioligands were prepared and also tested in preclinical studies (Oh et al. 2020; Wurzer et al. 2022).

A further and highly promising approach in the radiofluorination of biologically active molecules was originally described by McBride et al. in 2009, who proposed to use fluorine-18 in the form of a [^18^F]AlF^2+^ complex, that can then be coordinated by various chelators functionally linked to the molecule of interest (McBride et al. 2009). Differently to “classic” approach, which involves the formation of carbon–fluorine covalent bond, in this case fluorine-18 is first strongly bound to the metal, forming aluminum monofluoride [^18^F]AlF^2+^ at a tightly controlled pH, which can then be trapped by suitable chelators linked to the biomolecule, in aqueous media (McBride et al. 2009).

In recent years, many works related to this methodology have been carried out and published, and many biologically active molecules were radiolabelled (Hausner et al. 2020; Alonso Martinez et al. 2018; Tshibangu et al. 2020), including PSMA-ligands, testing different cyclic (e.g. NODA, DOTA, NOTA) and non-cyclic (e.g. HBED) chelators (Ajenjo et al. 2021; Al-Momani et al. 2017; Malik et al. 2015; Boschi et al. 2016; Schmitt and Moreau 2023), although the best results were obtained with macrocyclic chelators such as NODA (pentadentate 1,4,7-triazacyclononane-1,4-diacetic acid) or NOTA (hexadentate ligand 1,4,7-triazacyclononane-1,4,7-triacetic acid). Indeed, they proved to chelate [^18^F]AlF^2+^ complex with high yields and stability in solution and in vivo (Schmitt and Moreau 2023; Liu et al. 2019; Wang et al. 2022). Results showed that in order to introduce the aluminum-monofluoride species into the macrocycle, heating at 100–120 °C is mandatory. Moreover, in case of NODA chelator, the “N_3_O_2_” donor configuration of the chelator facilitates the formation of stable octahedral aluminum complexes and only one unique coordination site is kept for fluorine-18. Macrocyclic chelators exhibit a high stability once the metal complex is formed, but the rigid cyclic structure requires “harsh” conditions to be rearranged and to accommodate [^18^F]AlF^2+^ (D’Souza et al. 2011; Fersing et al. 2019).

A more recent work of Cleeren et al., showed the design and synthesis of new polydentate ligands that allow chelation of aluminum monofluoride at moderate temperatures (< 40 °C), due to a more flexible and non-cyclic structure, which allows to reduce activation energy of [^18^F]AlF^2+^ chelation (Cleeren et al. 2016).

Indeed, several non-cyclic chelators were synthesized, conjugated with PSMA and tested, but most of them resulted to be unstable, in vitro and in vivo, except for H_3_L_3_ chelators (Cleeren et al. 2016). Later, the same authors reported about a new chelator, namely RESCA, an acyclic pentadentate chelator with a N_2_O_3_ set of donor atoms, with increased rigidity and capable to bind [^18^F]AlF^2+^ complex at room temperature while at the same time having suitable stability (Cleeren et al. 2018).

**Fig. 1:**
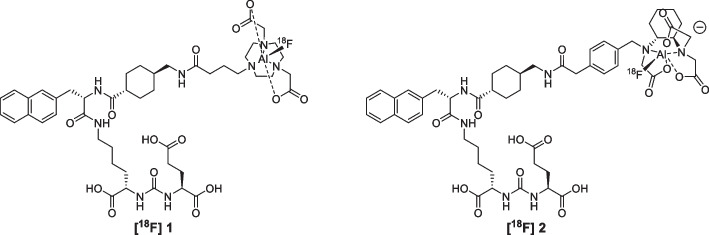
PSMA-617 derivatives prepared in this work: **[**^**18**^**F]1**: [^18^F]F-PSMA-617-NODA derivative, **[**^**18**^**F]2**: [^18^F]F-PSMA-617-RESCA derivative

The aim of the present work is the radiosynthesis, characterization and comparison of two new PSMA-617 derivatives functionalized with NODA and RESCA chelators (Fig. 1), respectively.

Furthermore, the radiosynthesis procedures have been fully automated, with the aim to implement a fast, efficient and “general purpose” method for the radiolabelling with [^18^F]AlF^2+^ of different biologically active macromolecules. Finally, the above radiolabelled derivatives have been tested in vivo in two preclinical cancer models (prostate and high grade glioma), to evaluate their pharmacokinetic properties, also by comparison with above mentioned well known and routinely used [^18^F]F-PSMA-1007.

## Results

### Cold chemistry

**Scheme 1:**
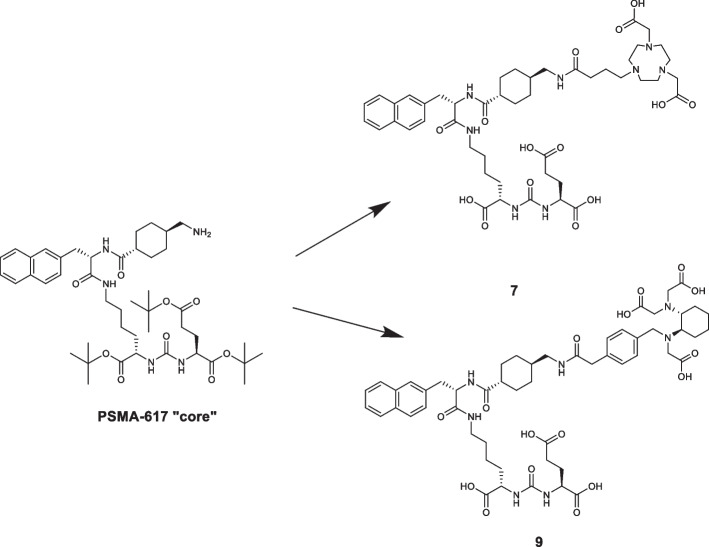
Syntheses of precursor **7** (PSMA-617-NODA derivative) and **9** (PSMA-617-RESCA derivative) starting from PSMA-617 “core”

The two PSMA-617 derivatives **[**^**18**^**F] 1** and **[**^**18**^**F] 2** shown in Fig. 1 were radiolabelled with [^18^F]AlF^2+^ starting from precursors **7** (PSMA-617-NODA precursor) and **9** (PSMA-617-RESCA precursor) shown in Scheme 1. The synthesis was designed adding specific linkers and chelators to the PSMA-617 “core”, the biologically active part of the molecule, whose synthetic strategy has been previously published (Iannone et al. 2022).

Precursor **7** was obtained starting from PSMA-617 “core”, commercial NO_2_AtBu (commercial NODA derivative) and 4-bromobutyric acid in order to provide a propyl linker. Precursor **9** was obtained by a similar procedure, but using a RESCA chelator derivative ((+)-RESCA-TFP).

**Scheme 2:**
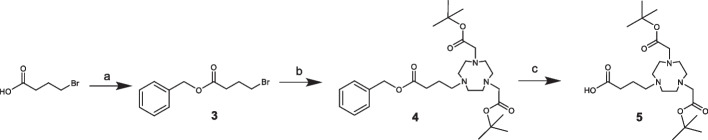
Synthesis of NODA-linker. Reagent and conditions: **a** PTSA·H_2_O, BzOH, cyclohexane, reflux, 10 h, 93%; **b** NO_2_A*t*Bu, K_2_CO_3_, dry ACN, rt, overnight, 79%; **c** H_2_, Pd/C 10%, EtOH, rt, overnight, 94% (Suppl. S1–S10, S53–S56)

In detail, the synthesis of precursor **7** was designed starting from commercial bromo-butyric acid and di-*tert*-butyl 2,2′-(1,4,7-triazacyclononane-1,4-diyl)diacetate (NO_2_A*t*Bu). Compound **3** was then reacted with NO_2_A*t*Bu via a nucleophilic substitution, yielding compound **4**. Finally, the benzylic group was removed by hydrogenolysis in ethanol (Scheme 2, reaction c) and compound **5** was obtained avoiding work up in aqueous media. Purification of **4** and **5** was not performed, due to their poor stability in chromatography column conditions. NODA-linker was obtained with an overall yield of 69%, and used as such for the subsequent synthetic steps.

**Scheme 3:**
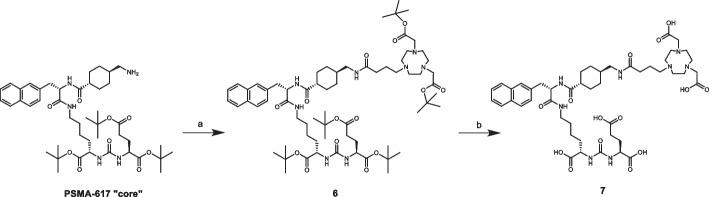
Synthesis of PSMA-617-NODA derivative (**7**). Reagent and conditions: **a** compound **5**, HATU, TEA, DMF, 0 °C-rt, 3 h, (> 99%); **b** 4 M HCl, dioxane, rt, 4 h, (45%) (Suppl. S11–S20, S30, S57–S61)

Compound **5** was then coupled with the PSMA-617 “core” in a two-step synthetic procedure. First, coupling reaction was conducted using 1-[bis(dimethylamino)methylene]-1H-1,2,3-triazole[4,5-b]pyridinium 3-oxide hexafluorophosphate (HATU) as a condensing agent in presence of triethylamine, and compound **6** was obtained in high yields (Scheme 3). Again, for the same reasons already depicted, no purification was performed at the end of the synthesis. *Tert*-butyl protecting groups were removed in acidic media. Finally, PSMA-617-NODA derivative precursor **7** was purified through semi-preparative RP-HPLC with an overall synthesis yield of ~ 31%, calculated from the starting 4-bromobutyric acid.

**Scheme 4:**
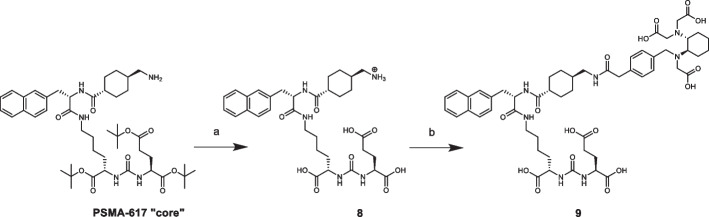
Synthesis of PSMA-617-RESCA derivative (**9**). Reagent and conditions: **a** TFA, DCM, rt, overnight, (59%); **b** (+)-RESCA-TFP, DMSO, 0.05 M buffer sodium acetate, rt, 4 h, (42%) (Suppl. S21–S29, S31, S35, S62–S66)

PSMA-617-RESCA derivative (precursor **9**) was also obtained after a two-step synthesis (Scheme 4). However, in this case PSMA-617 “core” was previously deprotected from tert-butyl esters in acidic media affording compound **8**. Due to the presence of by-products which can compete in the subsequent nucleophilic substitution step (*e.g.* via glutamate residue elimination), a purification by semi-preparative RP-HPLC was mandatory in order to obtain compound **8** with the requested purity. Afterwards, as already mentioned, introduction of RESCA chelator was performed using a commercial RESCA-derivative activated ester ((+)-RESCA-TFP); the reaction was conducted in acetate buffer at controlled pH of ~ 8.6 (Cleeren et al. 2018). Finally, precursor **9** was purified by semi-preparative RP-HPLC. The overall yield of this synthesis, calculated from starting PSMA-617 “core”, was ~ 25%.

### Radiochemistry

Precursors **7** and **9** were radiolabelled with [^18^F]AlF^2+^, following a fully automated process using a radiosynthesis system Trasis All-in-One (Additional file 1: S73). Radiolabelling procedures were implemented using “empty” cassettes, whose positions were filled as described in figure S73. Fluids were properly transferred through the cassette pathways by means of vacuum, nitrogen pressure and/or the electrically actuated syringe in position 9. The software sequence was adapted from a general-purpose procedure originally developed for the preparation of radiopharmaceuticals labelled with fluorine-18.

**Scheme 5:**
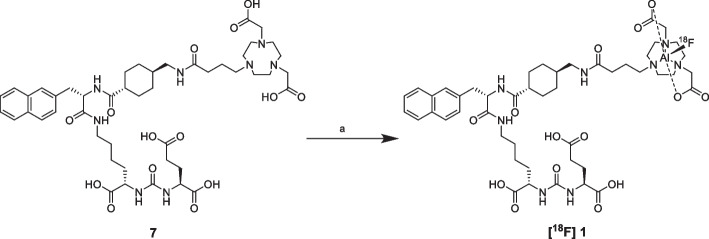
Radiosynthesis of **[**^**18**^**F]1**. Reagent and conditions: **a** [^18^F]AlF^2+^, 0.1 M sodium acetate buffer pH = 4, ethanol; 110 °C, 15 min

The major difference between the two radiosynthesic procedures relied on reaction temperature, while all the other parameters (e.g. reaction time, precursor amount and concentration, ratio AlCl_3_/biomolecule) were similar (Turolla et al. 2018). Indeed, according to the known behaviour of cyclic chelators, optimal conditions for the reaction with NODA-derivative were obtained by heating at 110 °C (Scheme 5) (D’Souza et al. 2011; Fersing et al. 2019).

**Fig. 2:**
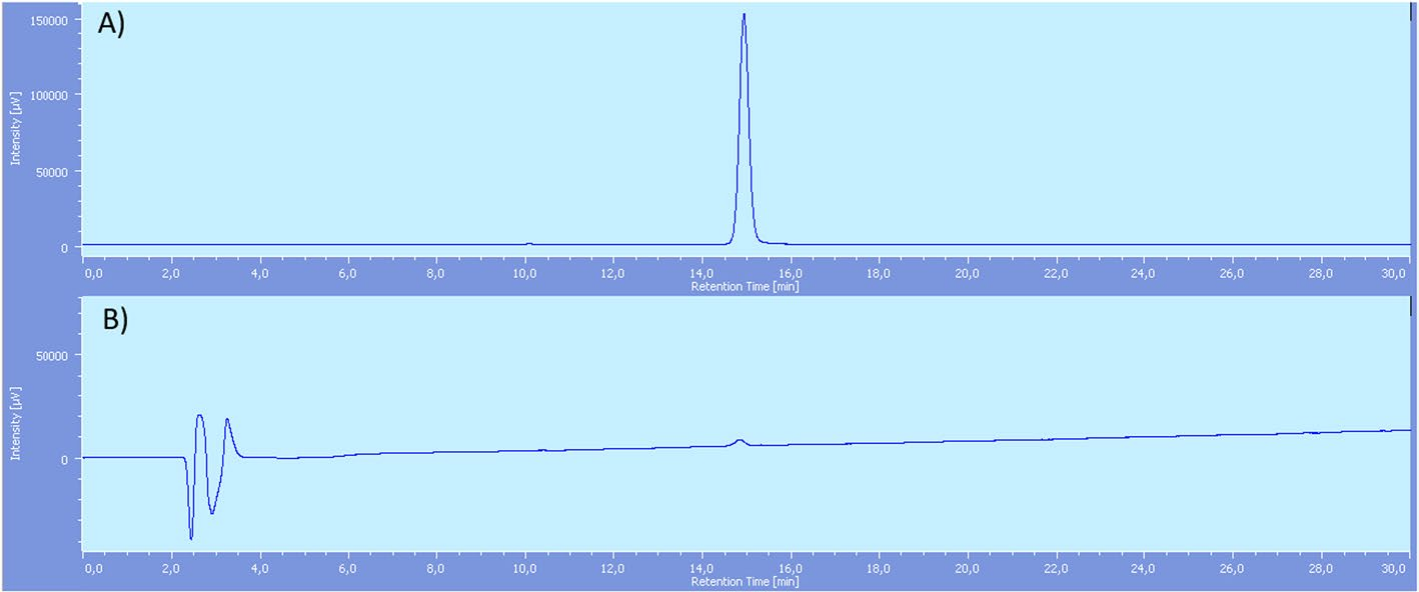
[^18^F]F-PSMA-617-NODA (**[**^**18**^**F]1)** after RP-HPLC purification: **A** radiochemical detector. **B** UV detector. Rt **[**^**18**^**F]1**: 14.9 min (Additional file 1: S34)

At first, aqueous fluorine-18 (around 40–50 GBq, see section "Radiochemistry") was converted to [^18^F]NaF in a QMA cartridge conditioned with NaCl solution. Then, [^18^F]AlF^2+^ was produced by reaction of [^18^F]NaF with AlCl_3_ in sodium acetate buffer at controlled pH = 4. Once [^18^F]AlF^2+^ was obtained, it was allowed to react with precursor **7** for 15 min in 0.1 M sodium acetate buffer pH = 4 (450 µl) and ethanol (350 µl), heating at 110 °C. As removal of by-products revealed to be troublesome using standard SPE cartridges (C_18_ Sep-Pak cartridge, see discussion in section "Discussion"), reaction mixture was then submitted to semi preparative RP-HPLC for purification. Finally, ~ 9–11 GBq at the end of synthesis (EOS) of product **[**^**18**^**F]1** were obtained, with > 99% of radiochemical purity (Fig. 2) and a RCY of 23 ± 3.3% (n = 4), not decay corrected (overall radiosynthesis time = 59 min, apparent molar activity(Coenen et al. 2017) 170 ± 46 GBq/μmol).

Purified product **[**^**18**^**F]1** was dissolved in physiological saline solution to have the final formulation suitable for in vivo preclinical testing.

Chemical and radiochemical stability in the final solution were tested by analytical HPLC and radio-TLC (Additional file 1: S37–S41); samples of the purified product were analyzed up to four hours after the end of radiosynthesis, at time intervals of two hours and storing the vial at room temperature. Under the above conditions, product **[**^**18**^**F]1** was stable and no loss of radioactivity due to radiolytic degradation and aluminum fluoride release were detected. **[**^**18**^**F]1** analyzed in the same conditions as above described, also proved to be stable in human plasma (Additional file 1: S42–S45).

**Scheme 6:**
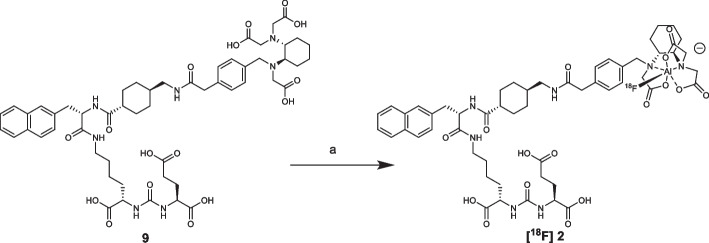
Radiosynthesis of **[**^**18**^**F]2**. Reagent and conditions: **a** [^18^F]AlF^2+^, 0.1 M sodium acetate buffer pH = 4, ethanol; rt, 15 min

As shown in Scheme 6, the [^18^F]AlF^2+^ reaction with precursor **9** was performed at the same reaction conditions (reagent amount and concentrations, reaction time, etc.) above described for the preparation of **[**^**18**^**F]1**, except for reaction temperature, which was set at room temperature.

 ~ 13–18 GBq at EOS of product **[**^**18**^**F]2** were obtained, with an overall RCY of 36 ± 4.9% (n = 4), non-decay corrected, with an overall radiosynthesis time of 42 min and an apparent molar activity 95 ± 20 GBq/μmol. Finally, product **[**^**18**^**F]2** was formulated with saline physiological solution to make it available for in vivo preclinical testing.

**Fig. 3:**
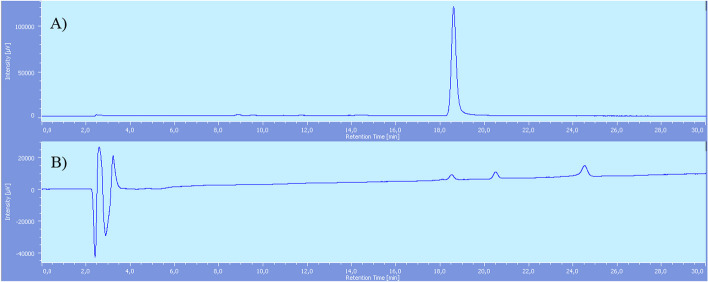
[^18^F]F-PSMA-617-RESCA (**[**^**18**^**F]2)** after SPE purification chromatograms: **A** Radiochemical detector. **B** UV detector. Rt [^18^F]AlF^2+^: 2.5 min, Rt product **[**^**18**^**F] 2**: 18.6 min, Rt PSMA-617-RESCA complexed with [Al(OH)]^2+^: 20.7 min, Rt precursor **9**: 24.7 min

In this case, purification was conducted by C_18_ Sep-Pak cartridges only and a suitable radiochemical purity of  > 95% was obtained (Fig. 3).

Molar activity was very high (967 ± 21 GBq/μmol) if calculated considering “cold” mass of **[**^**19**^**F]2** only. However, as shown in Fig. 3, also PSMA-617-RESCA complexed with [Al(OH)]^2+^ (Carroll et al. 2024) and precursor **9** were detected, and identified by injecting the respective reference standards. As the above by-products may compete with **[**^**19**^**F]2** in binding the receptor, it is more correct to refer to apparent molar activity(Coenen et al. 2017), which was calculated as 95 ± 20 GBq/μmol.

Stability in solution and in plasma of **[**^**18**^**F]2** were tested using analytical HPLC and radio-TLC and a gradual release of aluminum fluoride from the product was observed (Additional file 1: S46–S52), reaching a maximum of ~ 10% at four hours after end of synthesis.

### In vitro* experiments*

Competitive cell binding experiments were conducted using PC3-PIP cells, [^18^F]F-PSMA-1007, 300 nM PSMA-1007, and 8 different concentrations of PSMA-617-NODA (**7**) and PSMA-617-RESCA (**9**) (0, 0.01, 0.1, 1, 10, 100, 1000, 10,000 nM)*.* Experiments revealed an inhibition potency toward PMSA in the nanomolar range (IC_50_) (64 and 20 nM for PSMA-617-NODA and PSMA-617-RESCA, respectively) (Additional file 1: S67).

### In vivo biodistribution of PSMA radiotracers in a prostate *cancer* model

The uptake of [^18^F]F-PSMA-1007 and [^18^F]F-PSMA-617-NODA (**[**^**18**^**F]1**) was compared in vivo in a xenograft model of human prostate cancer obtained using LNCaP cells which are PSMA positive, as confirmed by real time PCR-analysis (Additional file 1: S69A). LNCaP tumour-bearing NSG mice were injected i.v. with either [^18^F]F-PSMA-1007 or [^18^F]F-PSMA-617-NODA (~ 3.8 MBq/mouse, ~ 20 pmol for [^18^F]F-PSMA-1007 and ~ 50 pmol **[**^**18**^**F]1**). Radiotracer uptake was assessed after 10, 60 and 120 min post injection by whole-body PET/CT acquisitions.

Table 1 summarizes the in vivo biodistribution of [^18^F]F-PSMA-1007 and [^18^F]F-PSMA-617-NODA (**[**^**18**^**F]1**) derivative. [^18^F]F-PSMA-617-NODA showed a rapid clearance especially in muscle, heart, liver, lung, small intestine and gall bladder compared to [^18^F]F-PSMA-1007. [^18^F]F-PSMA-617-NODA uptake of salivary glands was significantly lower than that of [^18^F]F-PSMA-1007 (Additional file 1: S68A). The uptake of the bone was similar for both the radiolabelled compounds (Additional file 1: S68B).

**Table 1:** In vivo biodistribution results of [^18^F]F-PSMA-1007 and [^18^F]F-PSMA-617-NODA ([^18^F]1)

Tissue (SUV mean)	[^18^F]F-PSMA-1007 (n = 4)			[^18^F]F-PSMA-617-NODA ([^18^F]1) (n = 4)		
	10’	60’	120’	10’	60’	120’
Muscle	0.23 ± 0.03	0.20 ± 0.04**	0.15 ± 0.05*	0.15 ± 0.05	0.04 ± 0.01	0.02 ± 0.01
Heart	0.38 ± 0.09	0.28 ± 0.06*	0.24 ± 0.03**	0.30 ± 0.03	0.09 ± 0.02	0.03 ± 0.01
Liver	0.50 ± 0.04**	0.33 ± 0.06*	0.21 ± 0.02***	0.22 ± 0.02	0.10 ± 0.02	0.04 ± 0.01
Kidney	9.55 ± 2.63	12.04 ± 2.49**	14.49 ± 1.41**	8.50 ± 1.40	6.39 ± 2.14	2.43 ± 1.24
Salivary gland	1.40 ± 0.25**	1.40 ± 0.20**	1.58 ± 0.12***	0.49 ± 0.05	0.18 ± 0.08	0.04 ± 0.02
Lung	0.46 ± 0.09*	0.40 ± 0.16*	0.31 ± 0.07**	0.31 ± 0.04	0.14 ± 0.01	0.04 ± 0.01
Small instestine	0.60 ± 0.05**	0.49 ± 0.12*	0.30 ± 0.07*	0.23 ± 0.05	0.16 ± 0.05	0.06 ± 0.03
Colon	0.35 ± 0.27	0.28 ± 0.14	0.26 ± 0.08*	0.30 ± 0.08	0.17 ± 0.02	0.13 ± 0.06
Bone	0.36 ± 0.09	0.26 ± 0.09	0.21 ± 0.08	0.20 ± 0.12	0.23 ± 0.11	0.25 ± 0.12
Gall bladder	0.99 ± 0.40*	1.02 ± 0.43*	0.75 ± 0.48	0.27 ± 0.05	0.14 ± 0.07	0.03 ± 0.02
Brain	0.12 ± 0.02**	0.09 ± 0.03*	0.10 ± 0.03*	0.05 ± 0.003	0.03 ± 0.01	0.02 ± 0.01

Data are reported as SUV mean of respective volumes of interest (VOI). Average values ± SD (*, *p* < 0.05, **, *p* < 0.01 and ***p < 0.001 by paired *t* test) were calculated per group at 10, 60 and 120 min

^*^p, **p, ***p versus [^18^F]F-PSMA-617-NODA

**Fig. 4:**
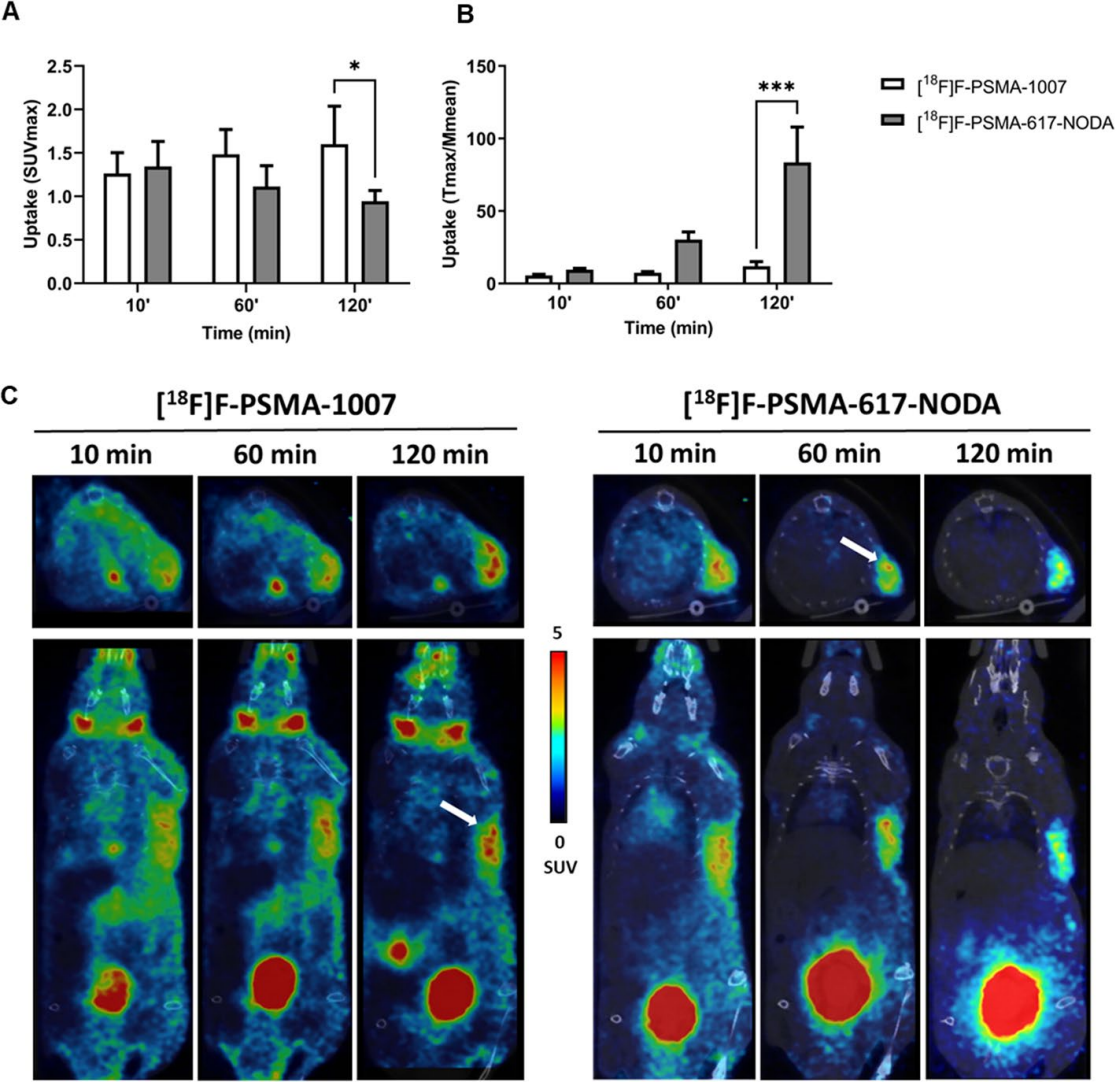
PET/CT uptake evaluation of [^18^F]F-PSMA-1007 and [^18^F]F-PSMA-617-NODA (**[**^**18**^**F]1**) in a subcutaneous prostate cancer model (LNCaP cells); comparison was performed by administering, in different days, the two radiolabelled products to the same 4 mice. **A** The quantification data are reported as SUV max of tumour uptake, and as **B** tumour to muscle ratios (tumour SUV max/muscle SUV mean). Bars, mean ± SD (n = 4 mice); *p < 0.05, **p < 0.01 and ***p < 0.001 by 2-way ANOVA Sidak’s multiple comparison test. **C** Representative PET/CT images in transaxial (upper) and coronal (bottom) viewers of the same mouse for each experimental condition (scale bar is reported as SUV mean). White arrows, LNCaP tumour

**[**^**18**^**F]1** showed a lower uptake in the tumour expressed as SUV max after 60 and 120 min (1.11 ± 0.24 and 0.94 ± 0.12) compared to [^18^F]F-PSMA-1007 (1.48 ± 0.29 and 1.60 ± 0.44) (Fig. 4A and Additional file 1: S69B) but uptake expressed as ratio between tumour and muscle (30.29 ± 10.46 at 60’ and 83.53 ± 48.71 at 120’ for [^18^F]F-PSMA-617-NODA; 7.46 ± 1.55 at 60’ and 11.99 ± 6.16 at 120’ for [^18^F]F-PSMA-1007) (Fig. 4B and Additional file 1: S69C) was significantly higher for **[**^**18**^**F]1**, as also visible in the PET images (Fig. 4C). The same results were obtained by comparison of respective tumour to blood ratio (Additional file 1: S70 A-B).

### [^18^F]F-PSMA-617-NODA ([^18^F]1) and [^18^F]F-PSMA-617-RESCA ([^18^F]2) tumour uptake in a glioma model

Subsequently, we evaluated the uptake of [^18^F]F-PSMA-617-NODA (**[**^**18**^**F]1**) and [^18^F]F-PSMA-617-RESCA (**[**^**18**^**F]2**) in an orthotopic glioma model obtained with Gli36ΔEGFR cells in female nude mice. Gli36ΔEGFR tumours-bearing nude mice (n = 3) were injected i.v. with either [^18^F]F-PSMA-617-NODA or [^18^F]F-PSMA-617-RESCA (~ 3.8 MBq/mouse, ~ 50 pmol for **[**^**18**^**F]1** and ~ 170 pmol **[**^**18**^**F]2**). Radiotracer uptake was assessed after 10, 60 and 120 min post injection by whole-body PET/CT acquisitions. Table 2 summarizes the peripheral uptake of the two radiotracers. Also in nude female mice, a rapid clearance of **[**^**18**^**F]1** in all organs and the same kinetic observed in NSG male mice (Additional file 1: S71A and B) were confirmed. In particular, we observed a significantly lower uptake of **[**^**18**^**F]1** in salivary glands, lungs and small intestine compared to **[**^**18**^**F]2**. It is interesting to note that the uptake of **[**^**18**^**F]1** in kidneys decreased over time in both models (Additional file 1: S71A and B), while the uptake of [^18^F]F-PSMA-1007 in NSG model and **[**^**18**^**F]2** in nude female mice model increased (Additional file 1: S71C and D).

**Table 2:** In vivo biodistribution results of [^18^F]F-PSMA-617-NODA ([^18^F]1) and [^18^F]F-PSMA-617-RESCA ([^18^F]2) derivatives

Tissue (SUV mean)	[^18^F]F-PSMA-617-NODA ([^18^F]1) (n = 3)			[^18^F]F-PSMA-617-RESCA ([^18^F]2) (n = 3)		
	10’	60’	120’	10’	60’	120’
Muscle	0.14 ± 0.01	0.07 ± 0.01	0.03 ± 0.01	0.19 ± 0.05	0.12 ± 0.02	0.11 ± 0.04
Heart	0.26 ± 0.05	0.10 ± 0.03	0.04 ± 0.002^§^	0.35 ± 0.05	0.17 ± 0.03	0.11 ± 0.03
Liver	0.22 ± 0.01^§^	0.11 ± 0.01	0.05 ± 0.01	0.98 ± 0.15	0.65 ± 0.22	0.30 ± 0.11
Kidney	13.11 ± 1.60^§^	13.32 ± 2.23	9.30 ± 3.60	6.92 ± 1.16	10.21 ± 0.80	10.64 ± 1.25
Salivary gland	0.54 ± 0.17	0.24 ± 0.09^§^	0.08 ± 0.04^§^	0.54 ± 0.14	0.45 ± 0.10	0.28 ± 0.10
Lung	0.20 ± 0.04	0.09 ± 0.04^§^	0.04 ± 0.01^§^	0.31 ± 0.01	0.19 ± 0.04	0.12 ± 0.03
Small instestine	0.32 ± 0.05	0.25 ± 0.06^§^	0.15 ± 0.06^§^	1.99 ± 1.44	3.07 ± 1.02	5.79 ± 2.07
Colon	0.37 ± 0.08	0.27 ± 0.13	0.16 ± 0.03	0.29 ± 0.23	0.78 ± 1.05	3.87 ± 5.37
Bone	0.18 ± 0.09	0.13 ± 0.08	0.12 ± 0.09	0.21 ± 0.03	0.20 ± 0.07	0.15 ± 0.11
Gall bladder	0.33 ± 0.11	0.10 ± 0.03	0.04 ± 0.01	3.28 ± 3.18	5.22 ± 4.23	5.07 ± 4.26
Brain	0.06 ± 0.01	0.03 ± 0.01^§^	0.02 ± 0.01^§^	0.09 ± 0.02	0.07 ± 0.02	0.07 ± 0.01

^§^*p* versus [^18^F]PSMA-617-RESCA

Data are reported as SUV mean of respective regions of interest. Average values ± SD (§, *p* < 0.05 by unpaired *t* test) were calculated per group at 10, 60 and 120 min

**Fig. 5:**
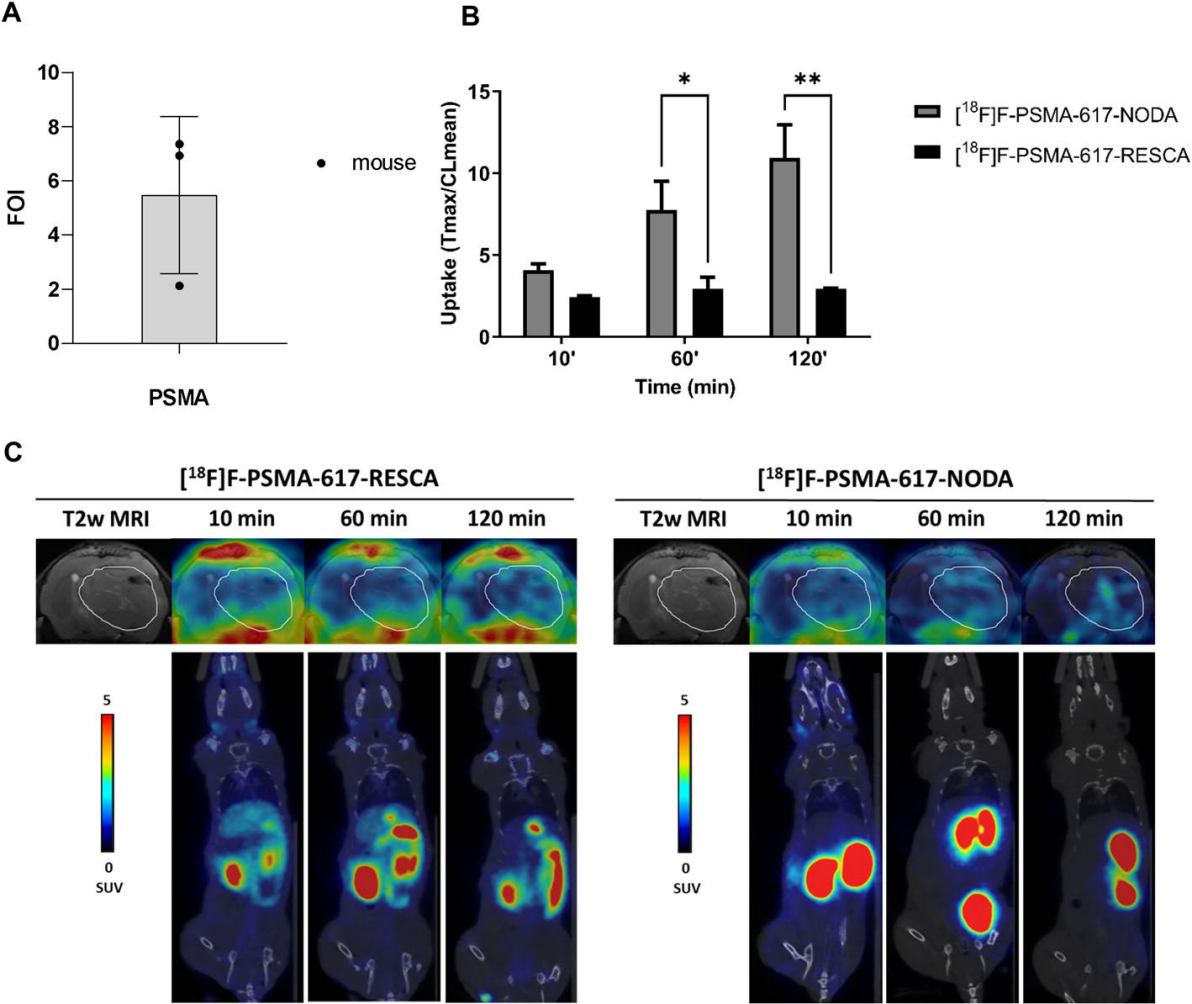
PET/CT uptake evaluation of [^18^F]F-PSMA-617-NODA (**[**^**18**^**F]1**) and [^18^F]F-PSMA-617-RESCA (**[**^**18**^**F]2**) in a glioma orthotopic model (Gli36ΔEGFR cells); comparison was performed by administering, in different days, the two radiolabelled products to the same mice (3 mice). **A** The relative quantification data of PSMA expression in glioma compared to contralateral healthy tissue, are reported as ΔΔct. **B** The tumour uptake results are expressed as tumour to contralateral ratios (tumour SUV max/contralateral SUV mean). Bars, mean ± SD (n = 3 mice); *p < 0.05, **p < 0.01 and ***p < 0.001 by 2-way ANOVA Sidak’s multiple comparison test. **C)** Representative PET/CT images, brain (transaxial, upper) and total body (coronal, bottom) of the same mouse for each experimental condition (scale bar is reported as SUV mean). White line indicates the glioma tumour area depicted by MRI images (T2w) and transferred to PET images

We confirmed the expression of PSMA in the Gli36ΔEGFR tumour, compared to healthy brain tissue, using *ex-vivo* real time PCR (Fig. 5A). After PET images quantification, [^18^F]F-PSMA-617-NODA (**[**^**18**^**F]1**) at 60 and 120 min showed a significant increase of tumour uptake, expressed as tumour-to-contralateral healthy brain (7.75 ± 3.06 at 60’ and 10.92 ± 3.56 at 120’) compared to [^18^F]F-PSMA-617-RESCA (**[**^**18**^**F]2**) (2.93 ± 1.23 at 60’ and 2.93 ± 0.08 at 120’) (Fig. 5B, C and Additional file 1: S72A). In detail, the absolute uptake of the tumour mass was the same for the two radiotracers but the healthy brain contralateral to the tumour showed a significantly lower uptake of [^18^F]F-PSMA-617-NODA compared to that of [^18^F]F-PSMA-617-RESCA (Additional file 1: S72B-D).

## Discussion

PSMA-617 proved to be a useful ligand, due to its favourable receptor interaction and pharmacokinetic, and it has been radiolabelled most notably with gallium-68, lutetium-177 and actinium-225, while only a few works have been published related to its radiolabelling with fluorine-18 in general, and using [^18^F]AlF^2+^ method in particular. For instance, in the work of Liu et al. (Liu et al. 2019) the PSMA-617 derivative PSMA-BCH has been radiolabelled with [^18^F]AlF^2+^, subsequently tested in mice PC tumour models, and compared with [^68^Ga]Ga-PSMA-617-NOTA; however, its preparation was performed manually, on a small scale. Thus, we believed that the development of a large scale, fully automated method for the radiolabelling of suitable PSMA-617 derivatives could be of interest and help to provide a straightforward method of preparation, potentially allowing radiopharmaceutical distribution to other Centres.

Indeed, this work was initially aimed to the development of two radiolabelled PSMA-617 derivatives (**[**^**18**^**F]1** and **[**^**18**^**F]2**), functionalized with the chelators NODA and RESCA, respectively, with [^18^F]AlF^2+^, following fully automated procedures that also allow to implement a "general purpose" methodology, that might then be exploited for the radiolabelling of other useful biological molecules of potential interest. A primary goal was also the preclinical evaluation of the new derivatives, and their comparison with [^18^F]F-PSMA-1007, which is currently the main radiopharmaceutical used in PET routine for PCa imaging.

Many scientific paper reported about chelators suitable for radiolabelling with [^18^F]AlF^2+^, to date. As already mentioned above, macrocyclic chelators require high temperatures (> 80 °C) to accomodate the aluminum-fluoride ion, but usually form very stable complexes, while acyclic pluridentate chelators, such as RESCA, have the great advantage of being able to chelate aluminum fluoride at room temperature, due to more favourable kinetic. On the other hand, it is well known that the former is thermodynamically stable while the latter may be more problematic, as only a few structures proved to be sufficiently stable.

The synthesis of a suitable PSMA-617-NODA derivative precursor was inspired by the work of Shetty et al. (Shetty et al. 2011): a study aimed to determine the relationship between NODA chelating agent, linker and radiolabelling efficiency. It took advantage of X-ray crystallography techniques to elucidate the structure of [^19^F]AlF-chelators complexes. One of their major findings was that, in terms of radiolabelling yield and stability, an ideal spacer between the chelator and the biomolecule should bear a propyl group. The above findings were further confirmed by the work of Wang et al., where it was noticed that propyl chains are able to reduce potential steric hindrance during the AlF^2+^ chelating process with NODA (Wang et al. 2022).

For this reason, the synthesis of a precursor (compound **7**) including a three-carbon atoms linker was designed, starting from commercially available bromo-butyric acid as shown above in Scheme 2. First attempts, performed linking the chelator to pure bromo-butyric acid through an S_N_2 nucleophilic substitution, were not satisfactory. Several attempts to protect acidic function using different esters (e.g. methyl and ethyl esters) were then performed, and subsequent S_N_2 reactions with NO_2_AtBu were successful, but its saponification to obtain compound **5** occurred in basic aqueous media. Indeed, workup of compound **5** in aqueous media revealed to be troublesome due to its zwitterionic form, and extraction by organic solvents was not successful. The best results were achieved by protecting –COOH as carboxybenzyl ester (compound **3**) (Springer et al. 2008), because its removal occurs under reducing conditions and no aqueous media is requested (Scheme 2).

The other steps of the synthesis of precursor **7** were straightforward. It is worth to mention that, due to the degradation of some intermediates on SiO_2_ chromatographic column, final purification of **7** was performed by RP-HPLC (Scheme 3).

PSMA-617-RESCA derivative (compound **9**) was obtained after a two-step synthesis (Scheme 4). The key factor here was the need to tightly control the pH, in the narrow range of 8.5–8.7, during the reaction between RESCA chelator and PSMA-617 “core” (Scheme 4, reaction b) (Cleeren et al. 2018). Indeed, adequate buffering is important for the efficiency of conjugation: a more basic pH promotes hydrolysis of active ester (+)-RESCA-TFP, while more acidic pH can protonate the amino group of PSMA-617 “core”.

Once the two precursors were obtained, their radiolabelling procedures with [^18^F]AlF^2+^ were implemented (Turolla et al. 2018), using an automated Trasis All-in-One radiosynthesis module (Additional file 1: Fig. S73). Preliminary tests were performed on a NODA chelator alone (NH_2_-MPAA-NODA) in order to find the best complexation conditions. In detail, several tests were performed at different temperature, reaction time, buffer molarity and chelator-AlCl_3_ equivalent ratios. Experimental results showed that the best complex coordination conditions were obtained with an equivalent ratio between AlCl_3_ and chelator of 1:2, heating at 110 °C, allowing the reaction to proceed for 15 min and using 0.1 M sodium acetate buffer pH = 4. All the subsequent radiosyntheses were performed consistently, using an average low amount (~ 0.2 mg) of precursors **7** and **9**.

In case of [^18^F]AlF^2+^ introduction into precursor **7**, reaction occurred heating at 110 °C (Scheme 5), which is compatible with tetra-peptide PSMA ligands structure.

**Fig. 6:**
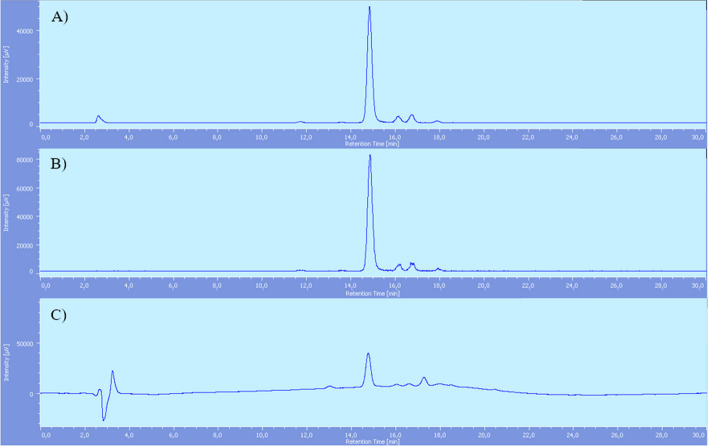
[^18^F]F-PSMA-617-NODA reaction mixture before (**A**) and after (**B**) C_18_ Sep-Pak cartridge purification (radiochemical detector) C) UV detector; reaction mixture after C_18_ Sep-Pak cartridge purification. Rt [^18^F] AlF^2+^: 2.5 min, Rt product **[**^**18**^**F]1**: 14.9 min, Rt PSMA-617-NODA derivative + [Al(OH)]^2+^: 14.9 min, Rt precursor** 7**: 17.2 min

The analysis of the crude reaction mixture based on HPLC (Fig. 6, entry a) showed an efficient coordination of [^18^F]AlF^2+^ with the chelator (only ~ 5% of free [^18^F]AlF^2+^ in solution).

The mixture was then passed through a C_18_ Sep-Pak cartridge (Fig. 6 entry b), which allowed to quantitatively remove [^18^F]AlF^2+^, but it was not effective with the other radioactive by-products, that represented an overall of ~ 14% of the total activity, based on combined analytical radio-HPLC and radio-TLC analyses. Our results are in line with data showed in the paper of Martin et al., who also found that the above impurities may be expected as a result of a thermally induced cyclization via condensation on Glu-urea-Lys residue (Martin et al. 2021). Since **[**^**18**^**F]1** is structurally very similar to the impurities, it was extremely difficult to separate them through SPE only, and a semi-preparative HPLC purification step was necessary to their complete removal (see Fig. 2, Additional file 1: S32-33).

Unfortunately, it was noticed that a by-product, consisting of PSMA-617-NODA complexed with [Al(OH)]^2+^, had a retention time fully overlapping with that of the product **[**^**18**^**F]1**, and they could not be separated even by semi-preparative HPLC. Such contamination led to a decreased molar activity. However, even considering in calculation the sum of the contributes coming from the “cold” **[**^**19**^**F]1** and the above by-product, the apparent molar activity resulted to be 170 ± 46 GBq/μmol.

**Fig. 7:**
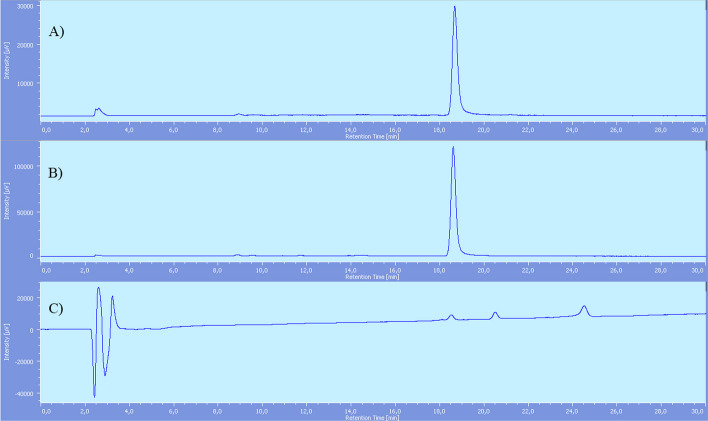
[^18^F]F-PSMA-617-RESCA (**[**^**18**^**F]2)** reaction mixture chromatograms: (**A**) Radiochemical detector; reaction mixture. (**B**) Radiochemical detector; reaction mixture after SPE purification. (**C**) UV detector; reaction mixture after SPE purification. Rt [^18^F] AlF^2+^: 2.5 min, Rt [^**18**^**F]2**: 18.6 min, Rt PSMA-617-RESCA complexed with [Al(OH)]^2+^: 20.7 min, Rt precursor **9**: 24.7 min. (Additional file 1: S36)

On the contrary, radiolabelling reaction of PSMA-617-RESCA derivative (precursor **9**) with [^18^F]AlF^2+^ occurred at room temperature (Scheme 6). Results showed an efficient [^18^F]AlF^2+^ coordination, with a radiochemical conversion of 84%, based on HPLC analysis of crude reaction mixture (Fig. 7). In this case, no radioactive by-products were formed, probably due to *milder* reaction conditions, and a purification with C18 Sep-pak cartridge was sufficient to obtain a product with > 95% of radiochemical purity, without the need for an additional semi-preparative HPLC purification step (Fig. 3).

In the end, the most significant difference between the two radiolabelling methods was represented by [^18^F]AlF^2+^ reaction temperature, which affected chemical and radiochemical purity of the products and reaction time. Indeed, comparing the above chromatograms (Figs. 6, 7) it is clear that the radiochemical quality of **[**^**18**^**F]2** was adequate after SPE purification, while **[**^**18**^**F]1** required an additional HPLC semi-preparative purification step, thus also extending the overall preparation time. The latter may be an important factor, in view of possible future application of the implemented "general purpose" method to the radiolabelling of other biologically active molecules.

As expected, compared to [^18^F]PSMA-617-RESCA, [^18^F]PSMA-617-NODA derivative showed better stability both in solution and in plasma, since after 4 h neither the release of [^18^F]AlF^2+^ in solution, nor the formation of other degradation by-products was noticed. Stability tests in plasma showed no substantial release of [^18^F]AlF^2+^ (see also Additional file 1: S37-S45) and protein uptake was ~ 11%.

On the contrary, in case of **[**^**18**^**F]2**, a significant release (up to 10%) of aluminum fluoride was observed at four hours after end of synthesis.

In detail, release of [^18^F]AlF^2+^ increased from 1.2% at EOS to 4.9% after 2 h of incubation at room temperature in solution, and then to 10% after 4 h. However, animal experiments should not have been affected significantly, considered that mice were injected with **[**^**18**^**F]2** approximately 85 min after EOS, when the amount of free [^18^F]AlF^2+^ in solution was supposed to be < 5% (Additional file 1: S46-S52).

The major drawback of the PSMA-targeting radiopharmaceuticals currently used for therapeutic purposes is the adverse toxicity due to their significant uptake in salivary glands and other PSMA-expressing organs (Klein-Nulent et al. 2018), which limits the dose that is possible to administer during PSMA-targeted radionuclide therapy (Heynickx et al. 2021b). To this regard, comparison of [^18^F]F-PSMA-617-NODA (**[**^**18**^**F]1)** and [^18^F]F-PSMA-1007 showed that the former has an encouraging biodistribution profile, with a lower uptake in salivary glands (Table 1), thus confirming biodistribution data obtained by Ruigrok et al. using [^177^Lu]Lu-PSMA-617 (Ruigrok et al. 2021). In addition, **[**^**18**^**F]1** displayed a faster clearance from all organs, which is also accompanied by a higher tumour-to-background ratio signal in LNCaP tumour compared to [^18^F]F-PSMA-1007. The above findings were confirmed in female mice (Table 2). Indeed, the uptake of **[**^**18**^**F]1** does not seem to be influenced by gender, as we observed the same biodistribution profile both in male and in female mice (Additional file 1: Fig. S71). The above results prompt for a further characterization of **[**^**18**^**F]1**, and potentially for its future use in patients affected by PCa and other tumour. As stated above, findings related to the non-PCa specific expression of PSMA receptors are continuously emerging, and HGG and glioblastoma (GBM), which are among the most highly invasive and aggressive brain tumours with dismal prognosis and limited therapeutic approach, are of increasing interest in exploring targets that may serve not only as diagnostics but also in radioligand therapy (RLT)(Truckenmueller et al. 2022). In glioma PSMA levels were found to be associated with vessels and, in turn, with patient’s outcome(Holzgreve et al. 2011). Recently, Truckenmueller et al.(Truckenmueller et al. 2022) correlated the [^68^Ga]Ga-PSMA uptake with histological PSMA expression in HGG relapsing patients and on the basis of TBRmax thresholds they selected patients for [^177^Lu]Lu-PSMA RLT. Due to the interesting biodistribution data of [^18^F]F-PSMA-617-NODA obtained in the prostate cancer model (LNCaP), as a proof-of-concept we evaluated the uptake of [^18^F]F-PSMA-617-NODA also in a glioma model and compared with the uptake of [^18^F]F-PSMA-617-RESCA.

**[**^**18**^**F]1** showed a faster clearance in all organs also compared to **[**^**18**^**F]2** (Table 2). For this reason, we decided to limit the development of the latter to glioma. In addition, we observed a higher tumour-to-contralateral ratio of [^18^F]F-PSMA-617-NODA compared to [^18^F]F-PSMA-617-RESCA (Additional file 1: S72). In detail, we observed the same absolute uptake of the two radiotracers in the tumour but a faster washout of [^18^F]F-PSMA-617-NODA from healthy brain contralateral to the tumor. For **[**^**18**^**F]1** absolute values of radioactivity concentration were lower in glioma than in prostate lesions in agreement with the prevalent vascular expression of PSMA in HGG, as previously stated. Modification in blood–brain–barrier (BBB) permeability might contribute to glioma/normal brain tissue positive ratios for both the radiotracers. In absence of a target, BBB permeability affects radioactivity uptake at early time after injection but is not involved in radiotracer retention at later times as we and other observed with PSMA ligands in glioma.

## Conclusions

Novel derivatives of PSMA-617 functionalized with NODA and RESCA chelator were synthesized and characterized. Both products were obtained in good yield, purity and apparent molar activity. Moreover, the whole radiolabelling procedures were fully automated.

Results showed marked differences in terms of reaction conditions and stability in solution and plasma. Indeed, NODA derivative (**[**^**18**^**F]1**) showed better stability in solution and in plasma, but in order to introduce [^18^F]AlF^2+^ heating at high temperatures was mandatory. Moreover, semi-preparative HPLC purification was necessary to obtain a product with suitable chemical and radiochemical purity. On the contrary, RESCA derivative (**[**^**18**^**F]2**) showed better radiochemical yield, also due to a shorter preparation time, milder reaction conditions and easy purification on SPE, proving its potential in the radiolabelling of large biomolecules in mild conditions, but also showing poor stability, thus limiting its use in vivo.

Furthermore, automation of radiolabelling procedure via “aluminum-fluoride introduction” was implemented, and similar protocols might be applied to the radiolabelling of other potentially interesting biomolecules (Tshibangu et al. 2020; Barnes et al. 2022; Allott et al. 2017). Of note, [^18^F]F-PSMA-617-NODA showed a more rapid clearance in non-target organs in comparison with the clinical established [^18^F]F-PSMA-1007 and the novel radiopharmaceuticals [^18^F]F-PSMA-617-RESCA. Moreover, [^18^F]F-PSMA-617-NODA showed high uptake levels expressed as tumour-to-background ratio not only in a prostate cancer model but also in glioma. Taken together, our results indicate the potential interest of [^18^F]F-PSMA-617-NODA for a further use into clinical practice.

## Material and methods

Solvents and reagents were purchased from Merck (Germany) and Carlo Erba (Italy).

Chelators NO_2_AtBu and ( +)-RESCA-TFP were purchased by Chematech (France).

TLC analyses were performed on silica gel 60 F254 pre-coated plates (Merck, Germany) by detection with a 5% phosphomolybdic acid solution in ethanol or 10% ninhydrin in butanol, and heating at 110 °C. TLC used for radio-TLC analyses were pre-coated TLC sheets ALUGRAM®Xtra (Macherey–Nagel, Germany).

Mass spectra ESI + were acquired using Impact HD™ UHR-QqToF (Bruker Daltonics, Germany) mass spectrometer (Resolution (max) in MS and MS/MS: 40,000 at m/z 1522) and by AmaZon ETD (Bruker Daltonics) ion trap mass spectrometer (Resolving power (max): up to 20,000 in full scan mode across the 50–3000 m/z range), depending on the required performances). Samples were solubilized in methanol and then infused in ESI source at a flow rate of 3 µL/min.

Concerning ion trap instrument, ESI source was used with the following settings: capillary − 4500 V, end plate offset − 500 V, nebulizer 20 psi, dry gas 9 l/min at 200 °C). Ions from the source were detected in a mass range of 70 to 1000 m/z, with 200,000 ICC, and 50 ms as maximum accumulation time. A target mass of 300 m/z and a trap drive of 100% were employed.

For UHR-QqToF spectrometer, parameters for the ESI source were set as follow: capillary 4000 V, end plate offset 500 V, nebulizer 0.3 bar, dry gas 4 l/min at 200 °C. Ions from the source were detected in a mass range from 50 to 3000 m/z. For Mass Spectrometer the following tuning was applied: Funnel 1 radiofrequency (RF) of 400 Vpp, Funnel 2 RF of 400 Vpp, Hexapole RF of 400 Vpp and a prepulse of 12 µs.

Fragmentation spectra for both the instrumentations were acquired using MRM modality and by optimizing both the isolation window and the fragmentation energy for each analyte.

Data results were processed by DataAnalysis™ 4.0 (Bruker Daltonics, Germany) software.

Concerning the ESI- and Elemental composition analysis, spectra were acquired using LC–MS, performed by Acquity UPLC- Synapt G2-Si QTof HDMS.

NMR spectra were recorded on a Bruker AVANCE 500 spectrometer equipped with a 5 mm broadband reverse probe with field *z*-gradient operating at 500.13 and 125.76 MHz for ^1^H and ^13^C, respectively. NMR spectra were recorded at 298 K in CDCl_3_, CD_3_OD or d-6 DMSO (isotopic enrichment 99.95%) solution and the chemical shifts were reported on a δ (ppm) scale. Data were collected and processed by XWIN-NMR software (Bruker Daltonics, Germany) running on a PC with Microsoft Windows 7. The samples were dissolved in the appropriate solvent in a 5 mm NMR tube. Acquisition parameters for 1D were as follows: ^1^H spectral width of 5000 Hz and 32 K data points providing a digital resolution of ca. 0.305 Hz per point, relaxation delay 10 s; ^13^C spectral width of 29,412 Hz and 64 K data points providing a digital resolution of ca. 0.898 Hz per point, relaxation delay 2 s. Chemical shifts (δ) of the ^1^H NMR and ^13^C NMR spectra are reported in ppm using the signal of residual solvent protons resonance as internal standard. ^1^H NMR: CDCl_3_ 7.26 ppm, CD_3_OD 3.31 ppm and d-6 DMSO 2.50 ppm; ^13^C NMR: CDCl_3_ 77.16 ppm (central line), CD_3_OD 49.00 ppm and d-6 DMSO 39.52 ppm. The splitting pattern abbreviations are as follows: s, singlet; d, doublet; t, triplet; q, quartet; m, multiplet, and br, broad signal. For two-dimensional experiments, Bruker microprograms using gradient selection (gs) were applied. All two-dimensional spectra (COSY, HSQC, HMBC) were acquired with 2048 data points for t_2_ and 256 for t_1_ increments.

[^18^F]fluoride was produced by a cyclotron (Cyclone 18/9, IBA, Belgium) via the ^18^O(p,n)^18^F nuclear reaction, by proton beam irradiation of a target containing 2 mL of > 97% enriched [^18^O]water (Taiyo Nippon Sanso, Japan).

Radioactive tests were carried out on a commercially available radiochemistry automated system (Trasis All-in-One) located in a suitably shielded hot cell (MIP-2, Comecer, Italy). Cassettes and reagent kits for the radiosynthesis of [^18^F]F-PSMA-1007 were purchased from Trasis (Belgium) and ABX (Germany), respectively.

Sep-Pak Light Waters Accel Plus QMA and SepPak C18 cartridges were from Waters Corp (USA).

Radiolabelled preparations and “cold” references were analyzed by RP-HPLC on a Jasco (Italy) PU-2089i system equipped with an automated injector, DAD detector, and radiochemical detector Raytest Gabi Star. Semi-preparative purification was carried out on a RP-HPLC equipped with a Perkin Elmer (USA) Flexar system quaternary pump and a Knauer WellChrom mod. K-2501 UV detector or on a Knauer P4.1S pump and a TOYDAD400 2Ch UV detector, connected to the automated module for radiosynthesis. Wavelength was set at 220 nm.

Analytical RP-HPLC column (Xterra C18 250 × 4.6 mm, 5 μm) was purchased from Waters Corp (USA), while semi-preparative RP-HPLC column (Clarity Oligo-RP C18, 250 × 10 mm, 5 µm) from Phenomenex (USA).

Radio TLC analyses were performed using a PerkinElmer Cyclone® Plus, equipped with Cyclone® Plus Phosphor Scanner and software OptiQuant™. The used dose calibrator was VIK-202 (Comecer, Italy).

### Chemistry

#### Benzyl 4-bromo butanoate (3)

A solution of commercial 4-bromobutyric acid (0.34 g, 1.93 mmol), benzylic alcohol (0.25 ml, 2.51 mmol) and *para*-toluene sulfonic acid monohydrate (37 mg, 2.514 mmol) in cyclohexane (20 ml) was brought to reflux. The reaction progress was monitored by TLC (hexane/EtOAc 9:1). After 10 h the solution was diluted in water (20 ml). The aqueous phase was extracted with EtOAc (3 × 20 ml). The collected organic phases were washed with brine (20 ml), dried over sodium sulfate and the solvent was removed at reduced pressure. The crude product was finally purified by silica gel column chromatography (eluent: gradient of DCM/acetone) to afford the pure **3** as a pale yellow liquid (0.46 g, 1.8 mmol, 93%).

R_f_: 0.8

Chemical and physical properties are in agreement with the reported ones (Springer et al. 2008).

#### di-*tert*-butyl 2,2′-(7-(4-(benzyloxy)-4-oxobutyl)-1,4,7-triazonane-1,4-diyl)diacetate (4)

Under anhydrous conditions, a suspension of commercial NO_2_A*t*Bu (50 mg, 0.14 mmol) and potassium carbonate (58 mg; 0.419 mmol) in dry acetonitrile (1 ml) was stirred at room temperature. Then a solution of compound **3** (54 mg; 0.21 mmol) in dry acetonitrile (2 ml) was added and the mixture was stirred overnight at room temperature. The reaction progress was monitored by TLC (DCM/MeOH 9:1 + 3 drops ammonium hydroxide 30% solution). The reaction was filtered through a folded paper filter and acetonitrile was evaporated to afford the desired product as a yellow semisolid (94 mg, 0.18 mmol, 79%).

R_f_: 0.25.

^1^H-NMR (CDCl_3_): δ 1.42 (s, 18H), 1.91 (br, 2H), 2.42 (t, 2H), 2.79 (s, 6H), 2.87–3.10 (br, 7H), 3.20–3.36 (m, 5H), 5.08 (d, 2H), 7.27–7.36 (m, 5H).

^13^C- NMR (CDCl_3_): δ 21.73, 28.19, 31.58, 54.37, 56.73, 58.97, 66.31, 81.08, 128.19, 128.24, 128.26, 128.57, 135.88, 171.14, 172.87.

MS (m/z) (ESI +) 534.3530 [M + H]^+^.

#### 4-(4,7-. bis(2-(*tert*-butoxy)-2-oxoethyl)-1,4,7-triazonan-1-yl)butanoic acid (5)

A suspension of Compound **4** (92 mg, 0.17 mmol), 10% Pd–C (49 mg) in ethanol (3 ml) was stirred overnight at room temperature under hydrogen flow. The reaction progress was monitored by TLC (DCM/MeOH + 3 drops ammonium hydroxide 30% solution, 8:2). At the starting material disappearance, the reaction was filtered through a Celite pad and the solvent was evaporated at reduced pressure. The desired crude product was obtained as a yellow semisolid (72 mg, 0.16 mmol, 94%).

^1^H-NMR (CDCl_3_): δ 1.42 (s, 18H), 2.06 (br, 2H), 2.48 (m, 2H), 2.58–2.90 (m, 3H), 2.91–3.29 (m, 5H), 3.29–3.62 (m, 8H).

^13^C- NMR (CDCl_3_): δ 21.17, 28.23, 31.59, 48.28, 51.68, 52.27, 55.06, 57.13, 170.81.

MS (m/z) (ESI +) 444.3070 [M + H]^+^.

#### di-*tert*-butyl (((S)-6-((S)-2-((1R,4S)-4-((4-(4,7-bis(2-(tert-butoxy)-2-oxoethyl)-1,4,7-triazonan-1-yl)butanamido)methyl)cyclohexane-1-carboxamido)-3-(naphthalen-2-yl)propanamido)-1-(tert-butoxy)-1-oxohexan-2-yl)carbamoyl)-L-glutamate (6)

Under anhydrous conditions, a solution of compound **5** (36 mg, 0.08 mmol), “PSMA-617”core (Cleeren et al. 2018) (67 mg, 0.08 mmol) and HATU (34 mg, 0.09 mmol) in DMF (3 ml) was cooled to 0 °C. TEA (0.03 ml, 0.20 mmol) was added dropwise over 5 min and the mixture was stirred 3 h at room temperature. Reaction was monitored by TLC (DCM/MeOH + 3 drops ammonium hydroxide 30% solution, 8.5:1.5). At the starting material disappearance, the mixture in DMF was dripped into H_2_O (30 ml) and the solution was extracted with EtOAc (3 × 30 ml). Collected organic phases were washed with brine (90 ml), dried over sodium sulfate and the solvent was removed at reduced pressure. The crude product **6** was obtained as a brown semisolid (100 mg, 0.08 mmol, > 99%).

R_f_: 0.5

^1^H-NMR (CD_3_OD): δ 0.83–1.02 (m, 3H), 1.20–1.37 (m, 8H), 1.44 (s, 45H), 1.52–1.65 (m, 3H), 1.65–1.84 (m, 4H), 2.03 (m, 3H), 2.15 (m, 1H), 2.31 (m, 4H), 2.71 (m, 1H), 2.90 (m, 9H), 3.02–3.13 (m, 3H), 3.22 (m, 5H), 3.30 (m, 4H), 3.46 (m, 2H), 4.05 (q, 1H), 4.19 (q, 1H), 4.65 (m, 1H), 7.35–7.47 (m, 3H), 7.67 (s, 1H), 7.74–7.83 (m, 3H).

^13^C-NMR (CD_3_OD): δ 20.25, 22.22, 26.91, 26.95, 26.98, 27.05, 27.71, 28.24, 28.38, 28.88, 29.54, 29.65, 31.12, 31.45, 31.77, 37.16, 38.02, 38.48, 44.50, 45.17, 46.55, 50.02, 50.81, 51.44, 52.79, 53.37, 54.52, 55.76, 80.37, 81.11, 81.30, 81.50, 125.25, 125.71, 127.12, 127.17, 127.26, 127.59, 127.64, 132.51, 133.52, 134.57, 158.52, 171.28, 172.21, 172.34, 172.54, 172.65, 177.09, 177.30.

MS (m/z) (ESI +) 1049.8066 [M + H]^+^.

#### (((S)-5-((S)-2-((1R,4S)-4-((4-(4,7-bis(carboxymethyl)-1,4,7-triazonan-1-yl)butanamido)methyl)cyclohexane-1-carboxamido)-3-(naphthalen-2-yl)propanamido)-1-carboxypentyl)carbamoyl)-L-glutamic acid (7)

Compound **6** (200 mg, 0.16 mmol) was dissolved in dioxane (5 ml). 4 M HCl (1.4 ml) was added dropwise in 2 h. The solution was stirred for 2 h and the reaction progress was monitored by analytical RP-HPLC. At the starting material disappearance, the solvent was removed at reduced pressure.

Crude mixture was purified by semi-preparative HPLC. Pure precursor **7** was obtained as a white solid (50 mg, 0.05 mmol, 45%).

Analytical RP-HPLC condition: column Xterra C18 5 µm, 250 × 4.6 mm; water + 0.1%TFA/acetonitrile + 0.1%TFA isocratic at 80:20 for 1 min, gradient from 80:20 to 60:40 in 30 min, gradient from 60:40 to 20:80 in 2 min; 1 ml/min, 220 nm, UV detector.

R_t_: 17.2 min.

Semi-preparative RP-HPLC condition: column Clarity Oligo-RP 5 µm, 250 × 10 mm; water + 0.1%TFA/acetonitrile + 0.1%TFA gradient from 80:20 to 60:40 in 30 min; 5 ml/min, 220 nm, UV detector.

R_t_: 18 min.

^1^H-NMR (CD_3_OD): δ 0.92 (m, 2H), 1.18–1.30 (m, 5H), 1.31–1.44 (m, 4H), 1.51 (m, 1H), 1.59 (m, 1H), 1.64–1.73 (m, 2H), 1.78 (m, 2H), 1.88 (m, 1H), 2.04 (m, 2H), 2.13 (m, 2H), 2.32 (t, 2H), 2.40 (m, 2H), 2.78 (m, 2H), 2.85 (m, 2H), 2.92–3.02 (m, 4H), 3.02–3.18 (m, 6H), 3.18–3.28 (m, 6H), 3.56 (d, 4H), 4.16 (m, 1H), 4.30 (m, 1H), 4.64 (t, 1H), 7.35–7.47 (m, 3H), 7.67 (s, 1H), 7.74–7.83 (m, 3H).

^13^C-NMR (CD_3_OD): δ 20.06, 22.20, 27.54, 28.19, 28.38, 28.82, 29.53, 29.63, 29.73, 31.43, 31.79, 37.18, 37.94, 38.43, 44.48, 45.16, 46.76, 50.23, 51.47, 52.12, 52.51, 54.53, 54.69, 54.85, 125.25, 125.71, 127.09, 127.15, 127.25, 127.57, 127.63, 132.51, 133.51, 134.53, 158.73, 172.23, 172.73, 173.48, 174.56, 175.01, 172.42.

MS (m/z) (TOF MS ESI-) 967.4794 [M-H]^−^.

#### ((1S,4R)-4-(((S)-1-(((S)-5-carboxy-5-(3-((S)-1,3-dicarboxypropyl)ureido)pentyl)amino)-3-(naphthalen-2-yl)-1-oxopropan-2-yl)carbamoyl)cyclohexyl)methanaminium (8)

Under anhydrous conditions, PSMA-617 “core” (Cleeren et al. 2018) (172 mg, 0.21 mmol) was dissolved in dichloromethane (4 ml). TFA was added dropwise (1 ml) and stirred at room temperature overnight. Reaction progress was monitored by analytical RP-HPLC. At the starting material disappearance, the solvent was removed at reduced pressure.

Crude mixture was purified by semi-preparative HPLC. Pure precursor **8** was obtained as a white solid (81 mg, 0.12 mmol, 59%).

Analytical RP-HPLC condition: column Xterra C18 5 µm, 250 × 4.6 mm; water + 0.1%TFA/acetonitrile + 0.1%TFA isocratic at 80:20 for 1 min, gradient from 80:20 to 60:40 in 30 min; 1 ml/min, 220 nm, UV detector.

R_t_: 14.3 min.

Semi-preparative RP-HPLC condition: column Clarity Oligo-RP 5 µm, 250 × 10 mm; water + 0.1%TFA/acetonitrile + 0.1%TFA gradient from 80:20 to 60:40 in 30 min; 5 ml/min, 220 nm, UV detector.

R_t_: 12.5 min.

^1^H-NMR (DMSO-d_6_): δ 0.80 (m, 2H), 1.02 (m, 1H), 1.20 (m, 3H), 1.28 (m, 2H), 1.34–1.52 (m, 3H), 1.57 (m, 1H), 1.67 (m, 4H), 1.89 (m, 1H), 2.06 (m, 1H), 2.21 (m, 2H), 2.59 (t, 2H), 2.89–3.13 (m, 4H), 4.00 (m, 1H), 4.07 (m, 1H), 4.52 (m, 1H), 6.28 (dd, 2H), 7.33–7–48 (m, 3H), 7.61 (br, 3H), 7.66 (s, 1H), 7.70–7.86 (m, 3H), 7.95 (m, 2H), 12.00–12.71 (br, 3H).

^13^C-NMR (DMSO-d_6_): δ 23.04, 28.02, 28.41, 28.81, 29.18, 29.37, 30.37, 32.19, 35.49, 38.69, 38.83, 43.63, 44.81, 52.14, 52.74, 54.08, 122.71, 126.00, 126.37, 127.71, 127.75, 127.92, 128.38, 132.24, 133.35, 136.22, 157.75, 171.44, 174.18, 174.62, 175.01, 175.11.

MS (m/z) (ESI+) 656.3288 [M + H]^+^.

#### (((S)-5-((S)-2-((1R,4S)-4-((2-(4-((((1R,2R)-2-(bis(carboxymethyl)amino)cyclohexyl)(carboxymethyl)amino)methyl)phenyl)acetamido)methyl)cyclohexane-1-carboxamido)-3-(naphthalen-2-yl)propanamido)-1-carboxypentyl)carbamoyl)-L-glutamic acid (9).

In a 0.05 M sodium bicarbonate buffer solution pH ~ 8.6 (2 ml), a solution of compound **8** (25 mg, 0.04 mmol) in DMSO (0.3 ml) was added. After pH correction to ~ 8.6 by adding dropwise a 2 M sodium carbonate buffer solution, a solution of commercial (+)-RESCA-TFP (23 mg, 0.04 mmol) in DMSO (0.2 ml) was added. pH was again corrected to ~ 8.6 and the solution was stirred at room temperature for 3 h.

Crude solution mixture was purified by semi-preparative HPLC. Pure precursor **9** was obtained as a white solid (18 mg, 0.02 mmol, 42%).

Analytical RP-HPLC condition: column Xterra C18 5 µm, 250 × 4.6 mm; water + 0.1%TFA/acetonitrile + 0.1%TFA isocratic at 80:20 for 1 min, gradient from 80:20 to 60:40 in 30 min; 1 ml/min, 220 nm, UV detector.

R_t_: 24.7 min.

Semi-preparative RP-HPLC condition: column Clarity Oligo-RP 5 µm, 250 × 10 mm; water + 0.1%TFA/acetonitrile + 0.1%TFA gradient from 80:20 to 50:50 in 40 min; 5 ml/min, 220 nm, UV detector.

R_t_: 25 min.

^1^H-NMR (CD_3_OD): δ 1.15–1.45 (m, 11H), 1.45–1.62 (m, 3H), 1.62–1.83 (m, 5H), 1.82–1.92 (m, 2H), 2.05–2.20 (m, 3H), 2.30 (d, 1H), 2.40 (m, 2H), 2.84–3.02 (m, 4H), 3.02–3.14 (m, 4H), 3.22 (m, 2H), 3.38 (m, 1H), 3.52 (m, 3H), 3.84 (d, 1H), 4.17 (m, 1H), 4.20–4.35 (m, 3H), 4.55 (d, 1H), 4.65 (t, 1H), 7.31–7.39 (m, 3H), 7.42 (m, 2H), 7.61 (m, 2H), 7.67 (s, 1H), 7.73–7.82 (m, 3H).

^13^C-NMR (CD_3_OD): δ 22.20, 23.87, 24.04, 25.06, 27.54, 28.19, 28.35, 28.84, 29.45, 29.59, 29.74, 31.44, 37.18, 37.96, 38.46, 42.08, 44.49, 45.25, 49.36, 52.14, 52.51, 53.83, 54.54, 57.32, 60.00, 63.85, 117.79, 125.26, 125.72, 127.10, 127.15, 127.25, 127.59, 127.64, 129.72, 131.29, 132.51, 133.51, 134.53, 138.09, 158.73, 161.11, 161.38, 168.06, 172.13, 172.24, 174.58, 175.03, 177.43.

MS (m/z) (TOF MS ESI–) 1079.4899 [M–H]^−^.

### Radiochemistry

#### (((S)-5-((S)-2-((1R,4S)-4-((4-(4,7-bis(carboxymethyl)-1,4,7-triazonan-1-yl)butanamido)methyl)cyclohexane-1-carboxamido)-3-(naphthalen-2-yl)propanamido)-1-carboxypentyl)carbamoyl)-L-glutamic acid [^18^F] aluminium fluoride ([^18^F] 1)

Aqueous [^18^F]fluoride solution, containing a range of 40–50 GBq, was passed through a Sep-Pak light QMA cartridge pre-conditioned with NaCl solution (10 ml). The cartridge was washed with 10 ml of ultrapure water. Then, 300 μl of 0.1 M sodium acetate buffer pH = 4 solution were passed through the cartridge eluting the activity in the Na^+^[^18^F]^−^ form directly in the reaction vial, where 50 μl of AlCl_3_ 2 mM solution were previously loaded (0.1 μmol, 24 μg). The reaction mixture was bubbled by nitrogen for 5 min at room temperature. Then, a 2 mM solution of precursor **7** (0.2 μmol, 0.2 mg) in 100 μl of 0.1 M sodium acetate buffer pH = 4 and 350 μl ethanol was added, and the reaction mixture was heated at 110 °C for 15 min. Then, in order to purify product **[**^**18**^**F] 1**, the mixture was diluted with 8 ml of 80:20 water + 0.1% TFA/ acetonitrile + 0.1% TFA and submitted to semi-preparative RP-HPLC. The fraction containing the desired product was collected, diluted with water (10 ml) and passed through a C_18_ Sep-Pak Plus cartridge previously conditioned with ethanol (10 ml) and water (10 ml). The cartridge was washed with water (10 ml) and the final product was obtained by elution with EtOH (1 ml) and saline physiological solution (19 ml) into the final container. The average obtained final RCY not corrected for decay was in the range of 23 ± 3.3% (n = 4), > 99% radiochemical purity (apparent molar activity 170 ± 46 GBq/μmol). Preparation time was 59 min.

Semi-preparative RP-HPLC condition: column Clarity Oligo-RP 5 µm, 250 × 10 mm; water + 0.1%TFA/acetonitrile + 0.1%TFA gradient from 80:20 to 60:40 in 30 min; 5 ml/min, 220 nm, UV detector.

R_t_: 20 min.

Analytical RP-HPLC condition: column XTerra C18 5 µm, 250 × 4.6 mm; water + 0.1%TFA/acetonitrile + 0.1%TFA isocratic at 80:20 for 1 min, gradient from 80:20 to 60:40 in 30 min, gradient from 60:40 to 20:80 in 2 min; 1 ml/min, 220 nm, UV detector.

R_t_: 14.9 min.

Radio-TLC conditions: Eluent: ammonium acetate 0.05 M, pH 5.5 / ACN 1:1.

R_f_: 0.7

#### (((S)-5-((S)-2-((1R,4S)-4-((2-(4-((((1R,2R)-2-(bis(carboxymethyl)amino)cyclohexyl)(carboxymethyl)amino)methyl)phenyl)acetamido)methyl)cyclohexane-1-carboxamido)-3-(naphthalen-2-yl)propanamido)-1-carboxypentyl)carbamoyl)-L-glutamic acid [^18^F] aluminium fluoride ([^18^F] 2)

Aqueous [^18^F]fluoride solution, containing a range of 40–50 GBq, was passed through a Sep-Pak light QMA cartridge pre-conditioned with NaCl solution (10 ml). The cartridge was washed with 10 ml of ultrapure water. Then, 300 μl of 0.1 M sodium acetate buffer pH = 4 solution were passed through the cartridge eluting the activity in the Na^+^[^18^F]^−^ form directly in the reaction vial, where 50 μl of AlCl_3_ 2 mM solution were previously loaded (0.1 μmol, 24 μg). The reaction mixture was bubbled by nitrogen for 5 min at room temperature. Then, a 2 mM solution of precursor **9 (**0.2 μmol, 0.21 mg) in 100 μl of 0.1 M sodium acetate buffer pH = 4 and 350 μl ethanol was added and the reaction mixture bubbled with nitrogen for 15 min at room temperature. Then, in order to purify product **[**^**18**^**F] 2**, the mixture was diluted with 15 ml of water and passed through C_18_ Sep-Pak Plus cartridge previously conditioned with ethanol (10 ml) and water (10 ml). The cartridge was washed with water (30 ml) and the final product was obtained by elution with EtOH (1 ml) and saline physiological solution (19 ml) into the final container. The average obtained final RCY not corrected for decay was in the range of 36 ± 4.9% (n = 4), > 95% radiochemical purity (Molar activity (A_m_) 967 ± 21 GBq/μmol, apparent molar activity 95 ± 20 GBq/μmol). Preparation time was 42 min.

Analytical RP-HPLC condition: column XTerra C18 5 µm, 250 × 4.6 mm; water + 0.1%TFA/acetonitrile + 0.1%TFA isocratic at 80:20 for 1 min, gradient from 80:20 to 60:40 in 30 min, gradient from 60:40 to 20:80 in 2 min; 1 ml/min, 220 nm, UV detector.

R_t_: 18.6 min.

Radio-TLC conditions: Eluent: ammonium acetate 0.05 M, pH 5.5 / ACN 1:1.

R_f_: 0.8

#### Radiosynthesis of [^18^F]F-PSMA-1007

Radiosynthesis of [^18^F]F-PSMA-1007 was performed using the same automated system platform previously described, and commercially available cassettes and reagent kits following a well-established radiosynthetic pathway (Giesel et al. 2017; Cardinale et al. 2017b). (RCY ndc = 45 ± 5%, radiochemical purity > 95%, overall time synthesis = 42 min, Molar activity 300 ± 19 GBq/μmol). See supporting information (S74).

#### Calibration curves

Calibration curve was created using 5 different concentrations (5, 10, 25, 50 and 250 nM) of PSMA NODA (**1**) and PSMA-RESCA precursors (**2**), respectively. Linearity (R^2^ > 0.99) and specificity (resolution > 2.5) comply with commonly accepted criteria.

### In vitro stability studies

Stability in solution of [^18^F]F-PSMA-617-NODA (**[**^**18**^**F]1**) and [^18^F]F-PSMA-617-RESCA (**[**^**18**^**F]2**) were evaluated injecting in RP-HPLC (for experimental conditions, see sections “(((S)-5-((S)-2-((1R,4S)-4-((4-(4,7-bis(carboxymethyl)-1,4,7-triazonan-1-yl)butanamido)methyl)cyclohexane-1-carboxamido)-3-(naphthalen-2-yl)propanamido)-1-carboxypentyl)carbamoyl)-L-glutamic acid [^18^F] aluminium fluoride ([^18^F] 1)” and “(((S)-5-((S)-2-((1R,4S)-4-((2-(4-((((1R,2R)-2-(bis(carboxymethyl)amino)cyclohexyl)(carboxymethyl)amino)methyl)phenyl)acetamido)methyl)cyclohexane-1-carboxamido)-3-(naphthalen-2-yl)propanamido)-1-carboxypentyl)carbamoyl)-L-glutamic acid [18F] aluminium fluoride ([18F] 2)”) samples (20 µl) of respective radiopharmaceutical solutions at t = 0 (EOS), after 2 h and after 4 h. Moreover, ≈ 7.5 kBq of **[**^**18**^**F]1** or **[**^**18**^**F]2** solution were deposited on TLC sheet. The TLC was imaged using Cyclone® Plus Phosphor Scanner and software OptiQuant™.

Stability in plasma was evaluated mixing aliquots of **[**^**18**^**F]1** or **[**^**18**^**F]2** solutions (10 MBq for 2 h incubation and 20 MBq for 4 h incubation samples) with 200 µL of human serum. The mixtures were incubated at 37 °C. Aliquots were taken at 2 h and 4 h. Plasma pellet was precipitated with 200 µL acetonitrile and centrifuged (13,500 rpm, 10 min). The supernatant was removed, and the activity of the supernatant solution and pellet was measured using a dose calibrator (VIK-202 Comecer). The supernatant solution was analyzed injecting in RP-HPLC (diluted 1:5 in water) and Radio-TLC as above described.

### *Study design of *in vivo* biodistribution*

Total body biodistribution of [^18^F]F-PSMA-1007, [^18^F]F-PSMA-617-NODA and [^18^F]F-PSMA-617-RESCA was evaluated in vivo using imaging PET/CT. [^18^F]F-PSMA-617-NODA uptake was compared to that of the [^18^F]F-PSMA-1007 in a PSMA positive prostate tumour xenograft model (LNCaP). [^18^F]F-PSMA-617-NODA and [^18^F]F-PSMA-617-RESCA uptake were also evaluated using an orthotopic glioma model (Gli36ΔEGFR).

### Cell cultures

LNCaP were maintained in RPMI1640 medium and Gli36ΔEGFR in Dulbecco’s Modified Eagle Medium (DMEM) with high glucose. Both medium were supplemented with 10% heat-inactivated Foetal Bovine Serum (FBS), 2 mM L-glutamine, and 50 IU/ml Penicillin/Streptomycin (P/S) (all Euroclone, UK). Both cells were incubated at 37 °C in a 5% CO_2_/95% air atmosphere. Cells were carefully cultured and monitored and in vitro displayed a typical growth pattern and phenotype.

### In vitro* uptake*

For in vitro uptake, the PC3-PIP cell line, which is isogenic to PC3 and stably expresses PSMA(Ghosh et al. 2005), and PC-3 cell line (PSMA negative) were used. 40.000 PC3-PIP and PC3 cells were seeded in 96-well cell culture plates and at the confluence were fixed adding 100 µl of 0.2% glutaraldehyde in PBS to medium. After 5 min incubation at 37 °C, the cells were washed six times with PBS. Then 200 µl of 0.1 M Glycine in PBS with 0.02% of sodium azide were added. After 5 min, the cells were washed 6 times with PBS and finally 250 µl of 1% BSA in PBS with 0.2% of sodium azide were added. The competitive cell binding assays were performed as follows: PC3-PIP fixed cells were incubated with [^18^F]F-PSMA-1007 at a concentration of 0.75 nM in the presence of 8 different concentrations of PSMA-617-NODA (**7**) and PSMA-617-RESCA (**9**) (0, 0.01, 0.1, 1, 10, 100, 1000, 10,000 nM) and of 300 nM of PSMA-1007. PC3 fixed cells were incubated with [^18^F]F-PSMA-1007 and used as negative control. After 60 min incubation at room temperature, cells were washed three times with PBS. Then cells were incubated for 10 min with NaOH (100 mM) and collected. The activity in the supernatants and cell pellets was determined using a gamma counter (1282 Compugamma CS, LKB Wallac, Finland). Cellular uptake was calculated as the percentage of radioactivity in cell pellets. The 50% inhibitory concentrations (IC_50_) were calculated by fitting the data with a nonlinear regression algorithm (Prism 9, GraphPad Software Inc., USA). Experiments were performed in triplicate.

### In vivo* models*

Animal experiments were carried out in compliance with institutional guidelines for the care and the use of experimental animals, which have been authorized by the Italian Ministry of Health (n° 378/2019-PR and 817/2021-PR). The animals were housed at constant temperature (23 °C) and relative humidity (40%) under a regular light/dark cycle with food and water available ad libitum. Seven to eight weeks old male NSG mice (Charles River Laboratories Italia s.r.l., Italy) were subcutaneously inoculated in the right flank with 5 × 10^6^ of PSMA‐positive human prostate cancer cells (LNCaP) suspended in matrigel (1: 1, total volume 200 ul). The growth tumour was monitored periodically with calliper and tumour volume was calculated using the following formula:

Tumour volume = (l^2^ * L)/2, where “l” is the minor side while “L” is the greater side.

When the tumour volume was about 230.8 ± 60.3 mm^3^ (approximately 15 days after tumour cells inoculation), 4 animals performed an in vivo biodistribution of [^18^F]F-PSMA-1007 and [^18^F]F-PSMA-617-NODA, on successive but non-consecutive days, using PET/CT.

5 × 10^5^ Gli36ΔEGFR cells were injected in the right striatum of seven to eight weeks old female nude mice (Envigo RMS s.r.l., Italy) as previously described (Valtorta et al. 2021). Ten days after mice underwent a T2-weighted Magnetic Resonance Imaging (MRI) using a 7 T small animal magnetic resonance scanner (Bruker, BioSpec 70/30 USR, Paravision 5.1, Germany) to confirm the growth of the tumour. Then, the same mice underwent in vivo biodistribution of [^18^F]F-PSMA-617-NODA and [^18^F]F-PSMA-617-RESCA, on successive but non-consecutive days, using PET/CT.

After cell injection, mice were monitored every day for body weight and clinical signs of disease (fur, eye, motor impairment) and sacrificed at the appearance of evident signs of illness or at the loss of more than 25% of the initial body weight.

### *Total body *in vivo* biodistribution*

Mice were injected into a tail vein with 3.8 ± 0.1 MBq of [^18^F]F-PSMA-1007, 4.0 ± 0.2 MBq of [^18^F]F-PSMA-617-NODA (**[**^**18**^**F]1**) and 3.8 ± 0.1 MBq of [^18^F]F-PSMA-617-RESCA (**[**^**18**^**F]2**), respectively. All the radiopharmaceuticals, used in each experiment, had a radiochemical purity greater than 95%.

Apparent molar activities at time of injection in case of LNCaP-NSG mice were 225 GBq/µmol for [^18^F]F-PSMA-1007 and 130 GBq/µmol for [^18^F]F-PSMA-617-NODA, respectively.

Apparent molar activities at time of injection in case of Gli36ΔEGFR-nu/nu mice were 172 GBq/µmol for [^18^F]F-PSMA-617-NODA and 72 GBq/μmol for [^18^F]F-PSMA-617-RESCA, respectively.

Total body PET/CT acquisitions were performed using PET/CT tomographs dedicated to small animals, X-ß-CUBE (Molecubes, Gent, Belgium) after 10, 60 and 120 min from tracer injection. Each acquisition lasted 30 min and mice were maintained anesthetized (4% isoflurane in induction and 2% in maintenance in air) during acquisition.

After the last study, mice were sacrificed and tumour collected to evaluate PSMA expression.

### Images quantification

Image quantification analysis was performed with PMOD 4.105 software (Zurich, Switzerland). Volumes of interest (VOIs) were drawn on peripheral organs (tumour, thoracic muscle, heart, liver, kidney, salivary glands, lung, small intestine, colon, bone, gall bladder) and on the healthy brain using CT images. For the glioma model, PET/CT images were co-registered to MRI images and two different VOIs were defined: (1) a control VOI covering the healthy left striatum (volume 4 mm^3^) was drawn on the axial MR images, adjusted on the other imaging planes and then copied on the PET images of each mouse; (2) a second glioma-covering VOI was drawn in the tumour-affected brain hemisphere and centered on mice lesions.

For quantification, Standardized Uptake Value (SUV) was calculated according to the formula:

SUV = organ concentration activity [MBq/g]/(injected activity [MBq]/animal weight [g]).

The data were expressed as standardized-uptake-values (SUVmean and SUVmax) and as tumour-to-muscle (T/M) and tumour-to-blood (T/B) ratio in PCa model and as tumour-to-contralateral (T/Cl) ratio in glioma model.

#### RNA extraction and Real-Time PCR

Post mortem, molecular analysis of PSMA expression has been evaluated on LNCaP tumour, Gli36ΔEGFR tumour and on healthy brain tissue by Real-Time PCR. Total RNA was isolated and it was reverse transcribed to cDNA using RNeasy Micro Kit (Qiagen, Germany) by following the manufacturer’s instructions. The Real-time PCRs were performed in duplicate for each data point using the Sybr Green technique; the oligonucleotides used were for β-actin forward: 5′-TCAAGATCATTGCTCCTCCTG-3′ and reverse: 5′-CCAGAGGCGTACAGGGATAG-3′; for PSMA forward: 5′-CAGGTCTGGAGCGAATTC-3′ and reverse: 5′-AGTCTCTCTCAATCTCAC-3′. The changes in target mRNA content in relation to the β-actin housekeeping gene were determined using the ΔΔct Method.

#### Statistical analysis

Data are presented as mean values ± standard deviation (SD). For statistical analysis, unpaired t-test Welch correction or 2-way ANOVA Sidak’s multiple comparison test were performed using Prism 9 (GraphPad Software Inc., USA). Differences were considered statistically significant when *p* < 0.05.

### Supplementary Information

**Supplementary Material**.

## Data Availability

Data used to generate results in the paper are available in the manuscript and in Supplementary Materials.

## Abbreviations

EMA: European Medicines Agency
EOS: End of synthesis
FDA: US Food and Drug Administration
GBM: Glioblastoma
HGG: High-grade glioma
HPLC: High-performance liquid chromatography
LNCaP: Lymph node carcinoma of the prostate
NO_2_A*t*Bu: Di-*tert*-butyl 2,2′-(1,4,7-triazacyclononane-1,4-diyl)diacetate
NODA: Pentadentate 1,4,7-triazacyclononane-1,4-diacetic acid
NOTA: Hexadentate ligand 1,4,7-triazacyclononane-1,4,7-triacetic acid
NSG mouse: NOD Scid Gamma mouse
PCa: Prostate cancer
PET: Positron emission tomography
PSMA: Prostate-specific membrane antigen
QMA cartridge: Quaternary methyl ammonium cartridge
RCY: Radiochemical yield
RESCA: Restrained complexing agent
SPE: Solid phase extraction
SUV: Standardized uptake value
TLC: Thin-layer chromatography
VOI: Volumes of interest
