# Supplementary material for: Automated radiosynthesis and preclinical evaluation of two new PSMA-617 derivatives radiolabelled via [18F]AlF2+ method
Source: EJNMMI Radiopharm Chem. 2024 Jun 21;9:50. doi: 10.1186/s41181-024-00280-0 (PMC11192711; doi:10.1186/s41181-024-00280-0)
Supplement: Supplementary file 1 — Supplementary Material. [file 41181_2024_280_MOESM1_ESM.pdf]

SUPPORTING INFORMATION

Automated radiosynthesis and preclinical evaluation of two new PSMA-617 derivatives radiolabelled via $[^{18}F]AlF^{2+}$ method.

Marco Nicola Iannone[1*], Silvia Valtorta[2,3,4*] Stefano Stucchi[1], Stefano Altomonte[5], Elia Anna Turolla[1], Elisa Vino[1], Paolo Rainone[4,5], Valentina Zecca[2,4], Alessia Lo Dico[2,3], Marco Maspero[2,4], Mariangela Figini[6], Matteo Bellone[7], Samuele Ciceri[8], Diego Colombo[8], Clizia Chinello[5], Lisa Pagani[5], Rosa Maria Moresco[2,4,5], Sergio Todde[1,2], Patrizia Ferraboschi[8].

[1] University of Milano-Bicocca, Tecnomed Foundation, Monza, Italy.

[2] Institute of Molecular Bioimaging and Physiology (IBFM), National Research Council (CNR), Segrate, Italy.

[3] NBFC, National Biodiversity Future Center, Palermo, Italy.

[4] Department of Nuclear Medicine, San Raffaele Scientific Institute, IRCCS, Milano, Italy.

[5] School of Medicine and Surgery, University of Milano-Bicocca, Monza, Italy.

[6]ANP2, Department of Advanced Diagnostics, Fondazione IRCCS, Istituto Nazionale dei Tumori, Milan, Italy

[7] Division of Immunology, Transplantation and Infectious Diseases, San Raffaele Scientific Institute, IRCCS, Milano, Italy.

[8] University of Milano, Department of Medical Biotechnologies and Translational Medicine, Milan, Italy.

*These authors contributed equally to this work.

Correspondence: Marco Nicola Iannone marco.iannone@unimib.it.

Contents

- NMR spectra of compounds **4**, **5**, **6, 7, 8, 9**
- RP-HPLC chromatograms of compounds **7, 8, 9, [18F]1, [18F]2**
- RP-HPLC chromatograms and radio-TLC for in vitro stability of **$[^{18}F]AlF^{2+}$, $[^{18}F]$1, $[^{18}F]$2**
- MS spectra of compounds **4**, **5**, **6, 7, 8, 9**
- Biodistribution images

NMR ANALYSIS

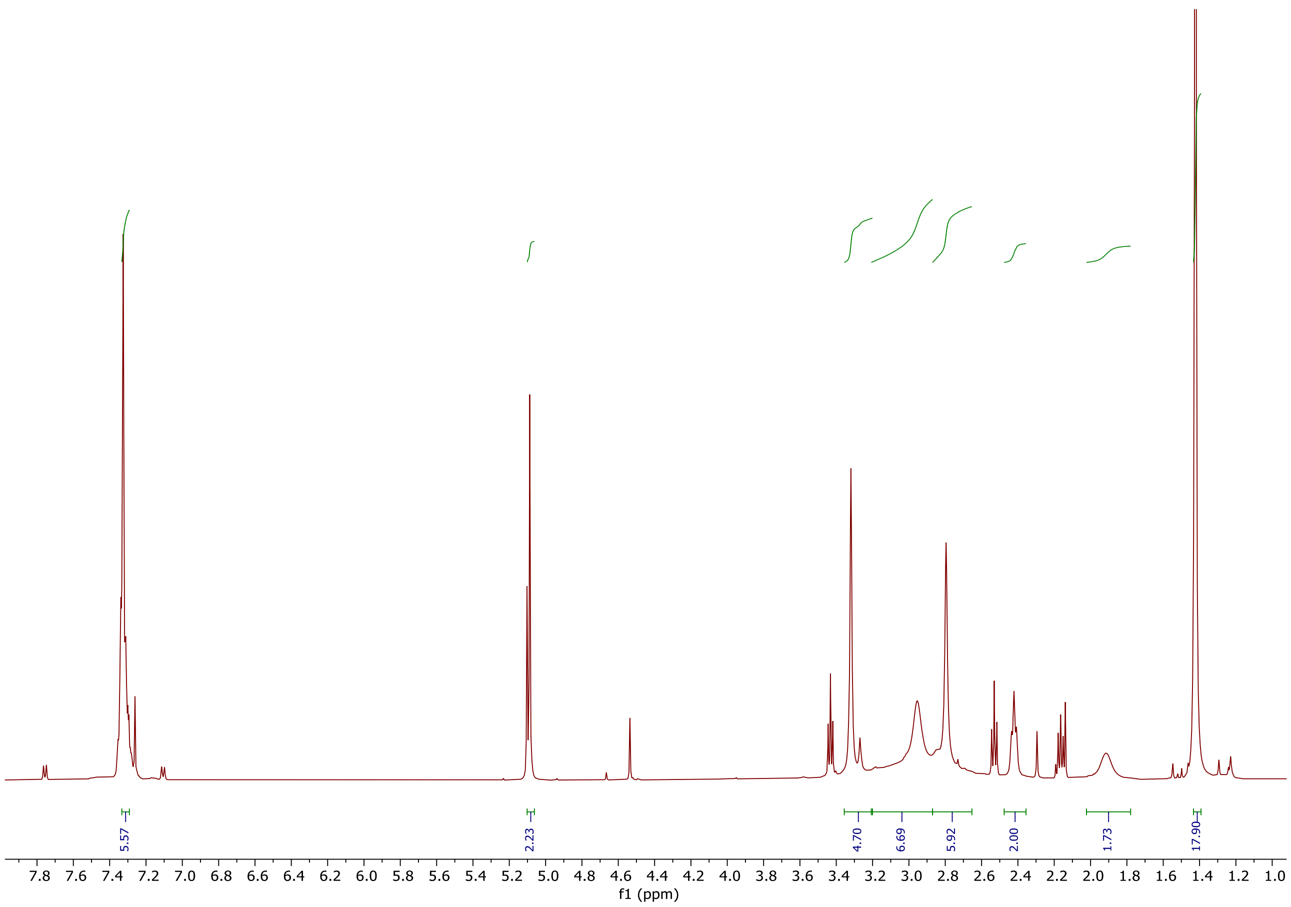

Figure S1: ^{1}H-NMR of Compound **4**

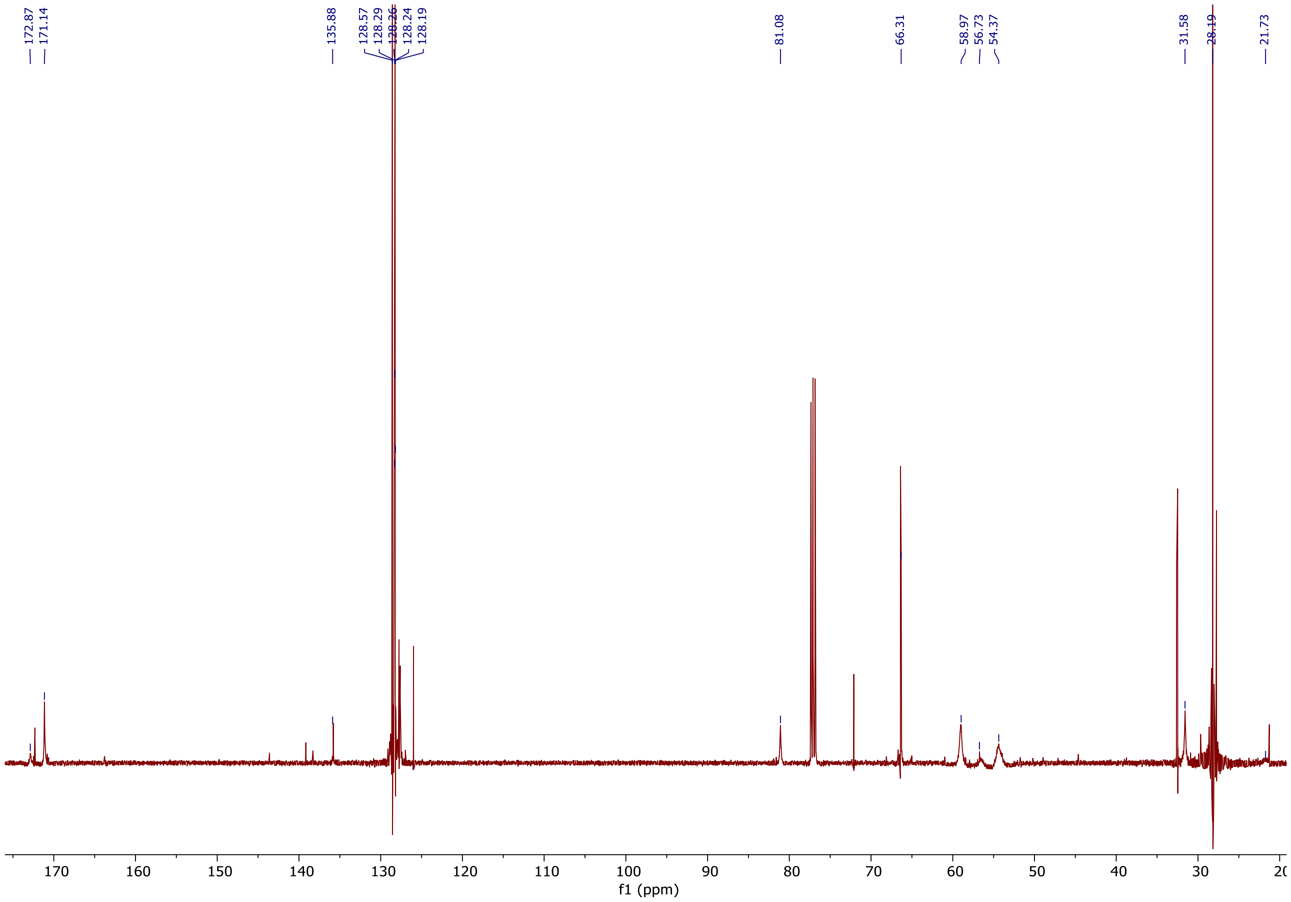

Figure S2: ^{13}C-NMR of Compound **4**

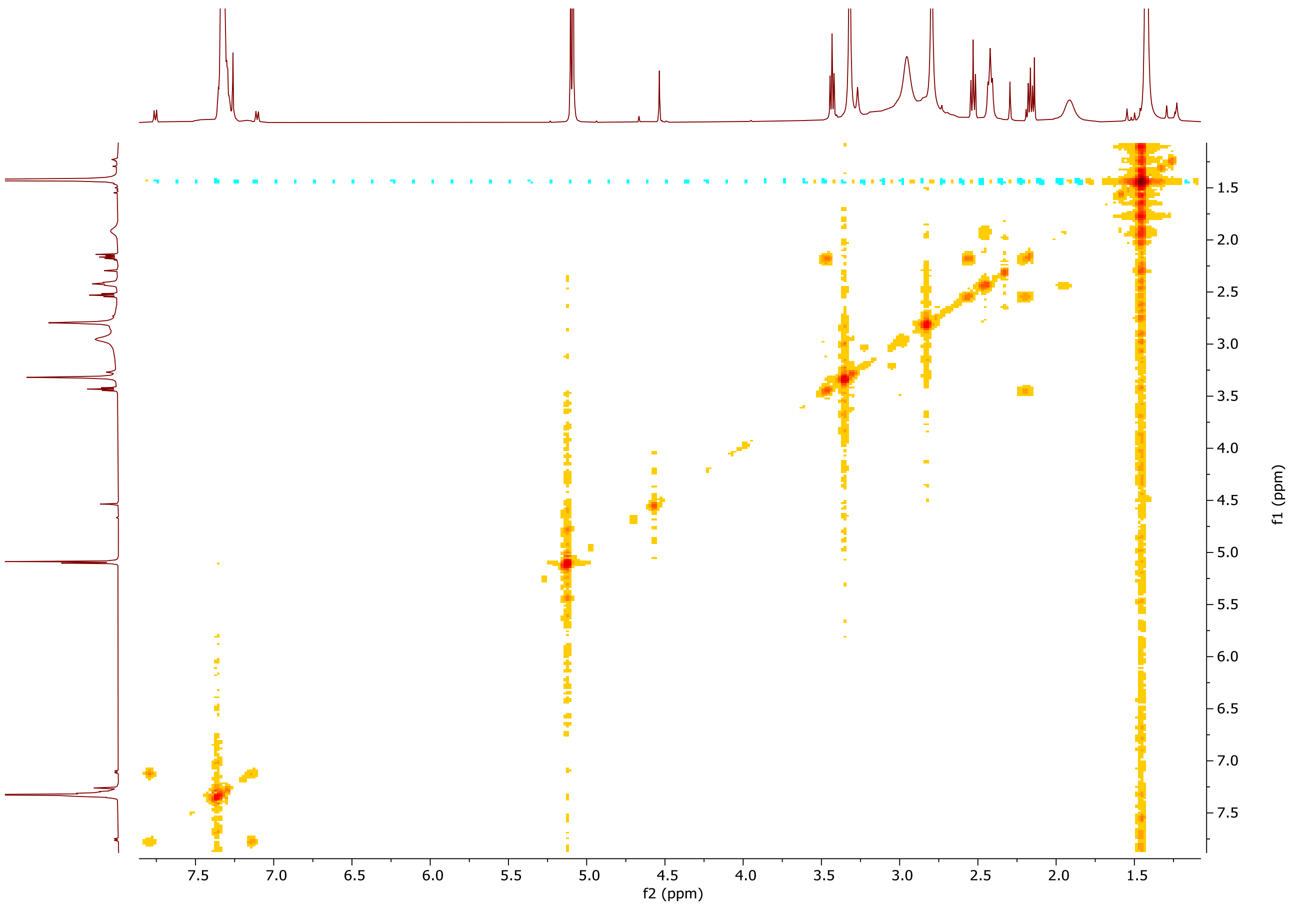

Figure S3: COSY-NMR of Compound **4**

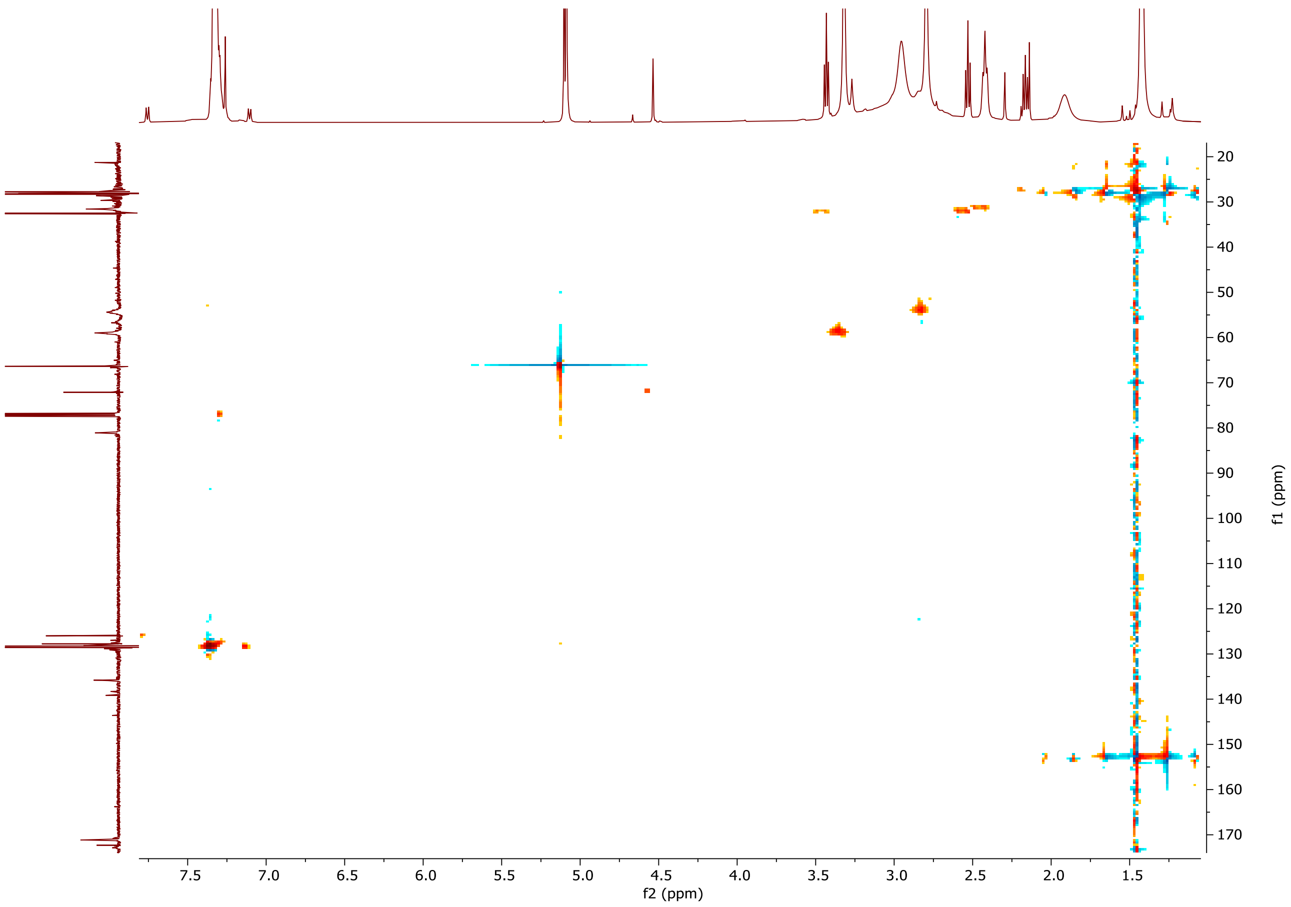

Figure S4: HSQC-NMR of Compound **4**

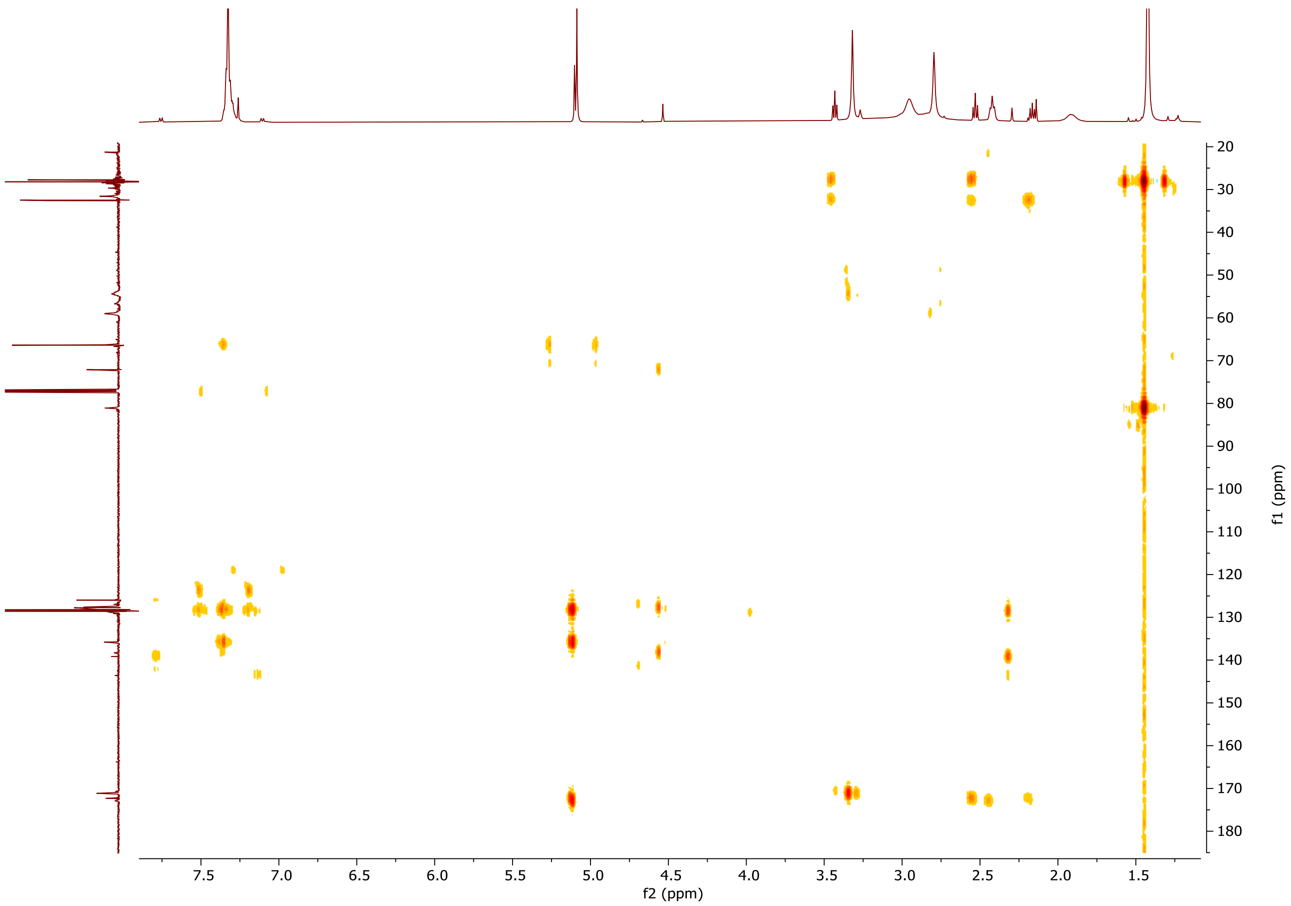

Figure S5: HMBC-NMR of Compound **4**

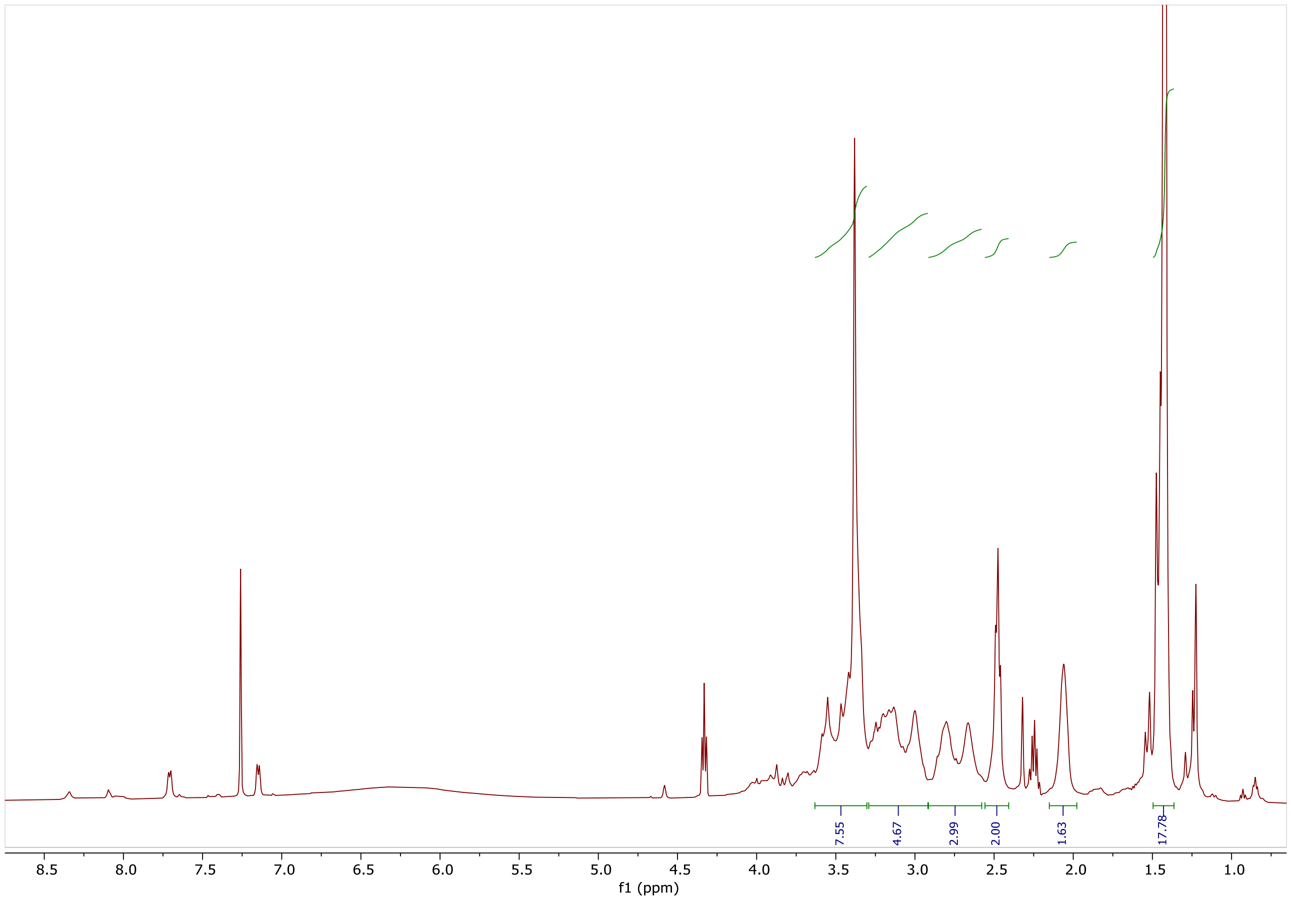

Figure S6: ^{1}H-NMR of Compound **5**

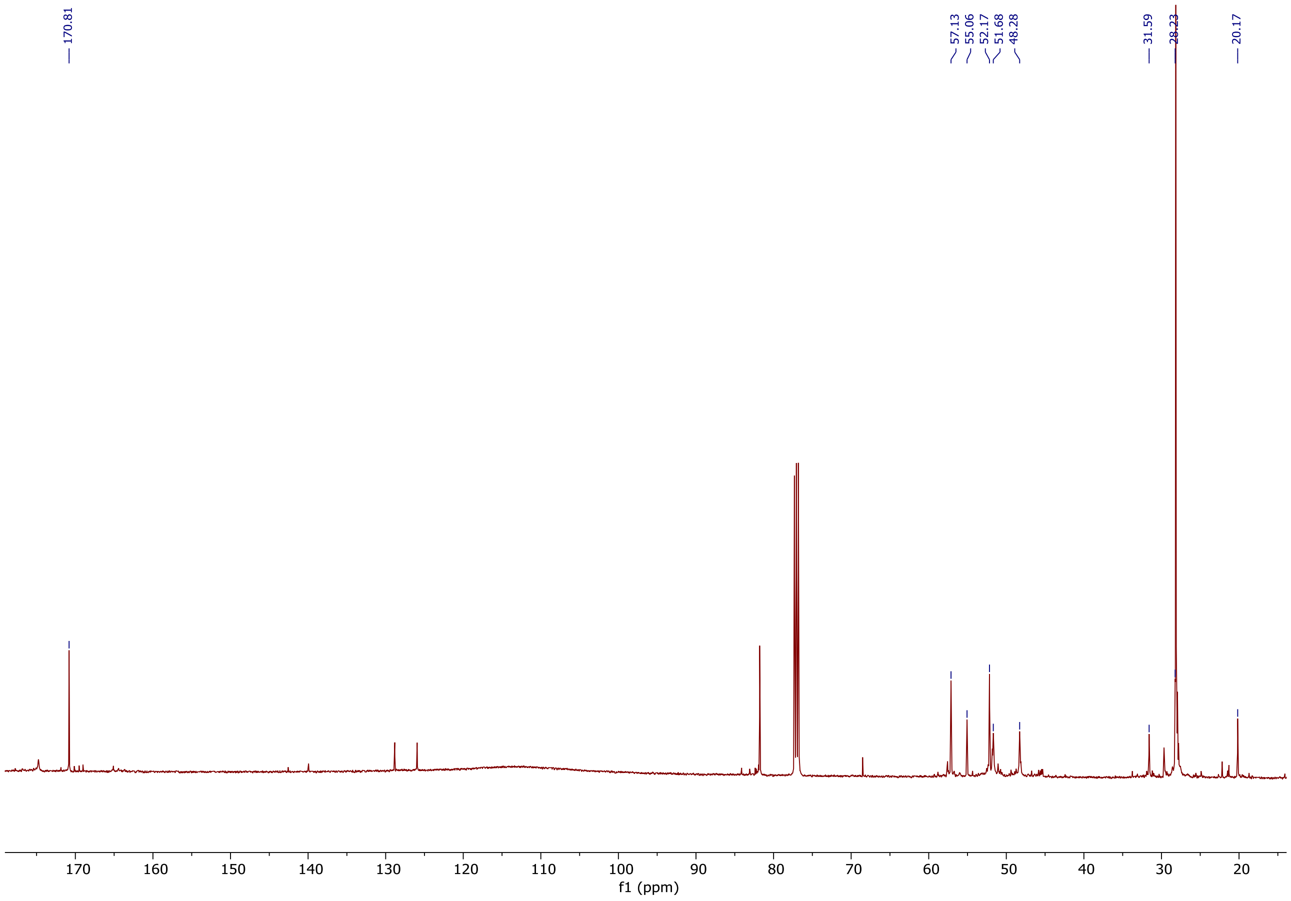

Figure S7: ^{13}C-NMR of Compound **5**

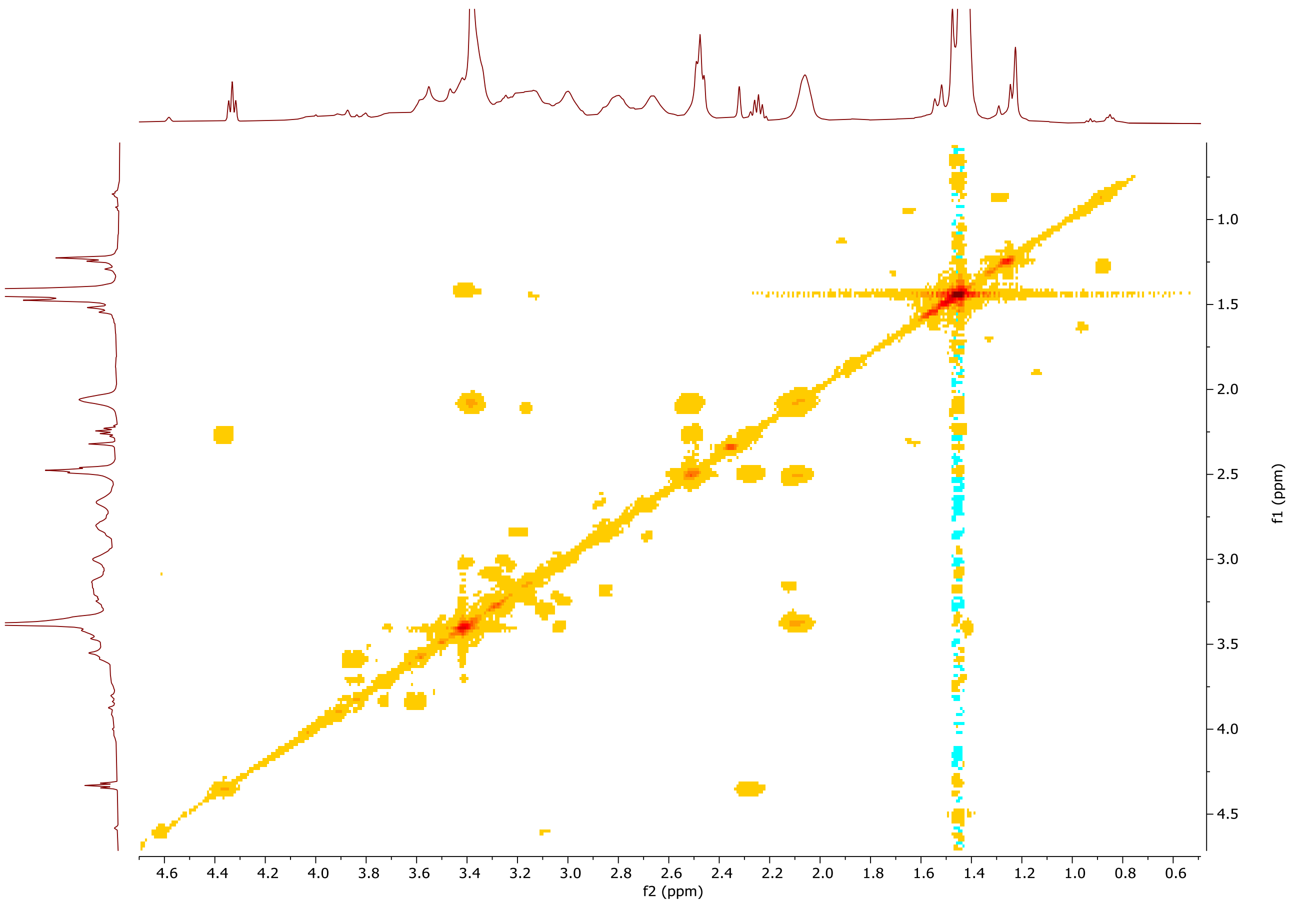

Figure S8: COSY-NMR of Compound **5**

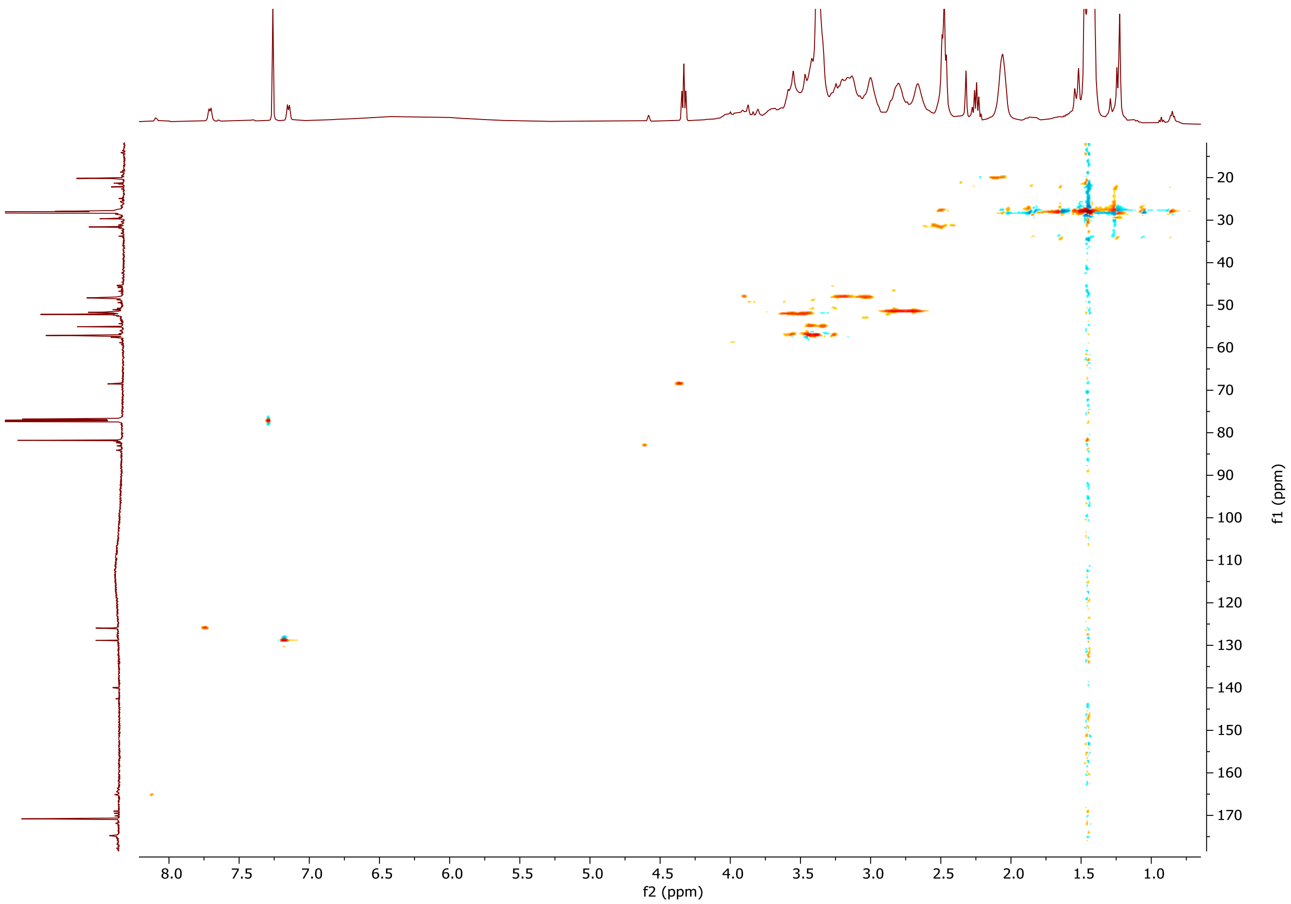

Figure S9: HSQC-NMR of Compound **5**

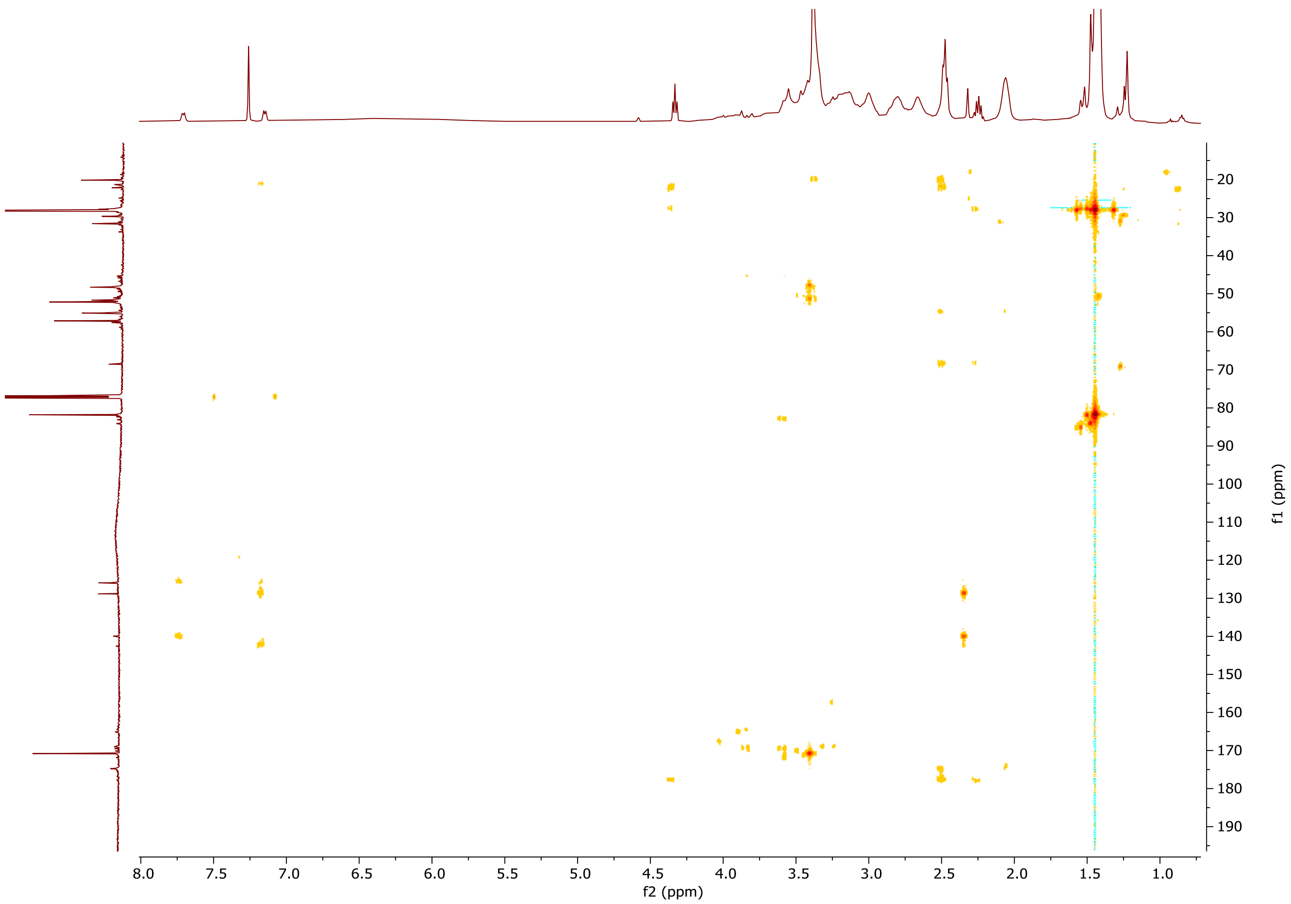

Figure S10: HMBC-NMR of Compound **5**

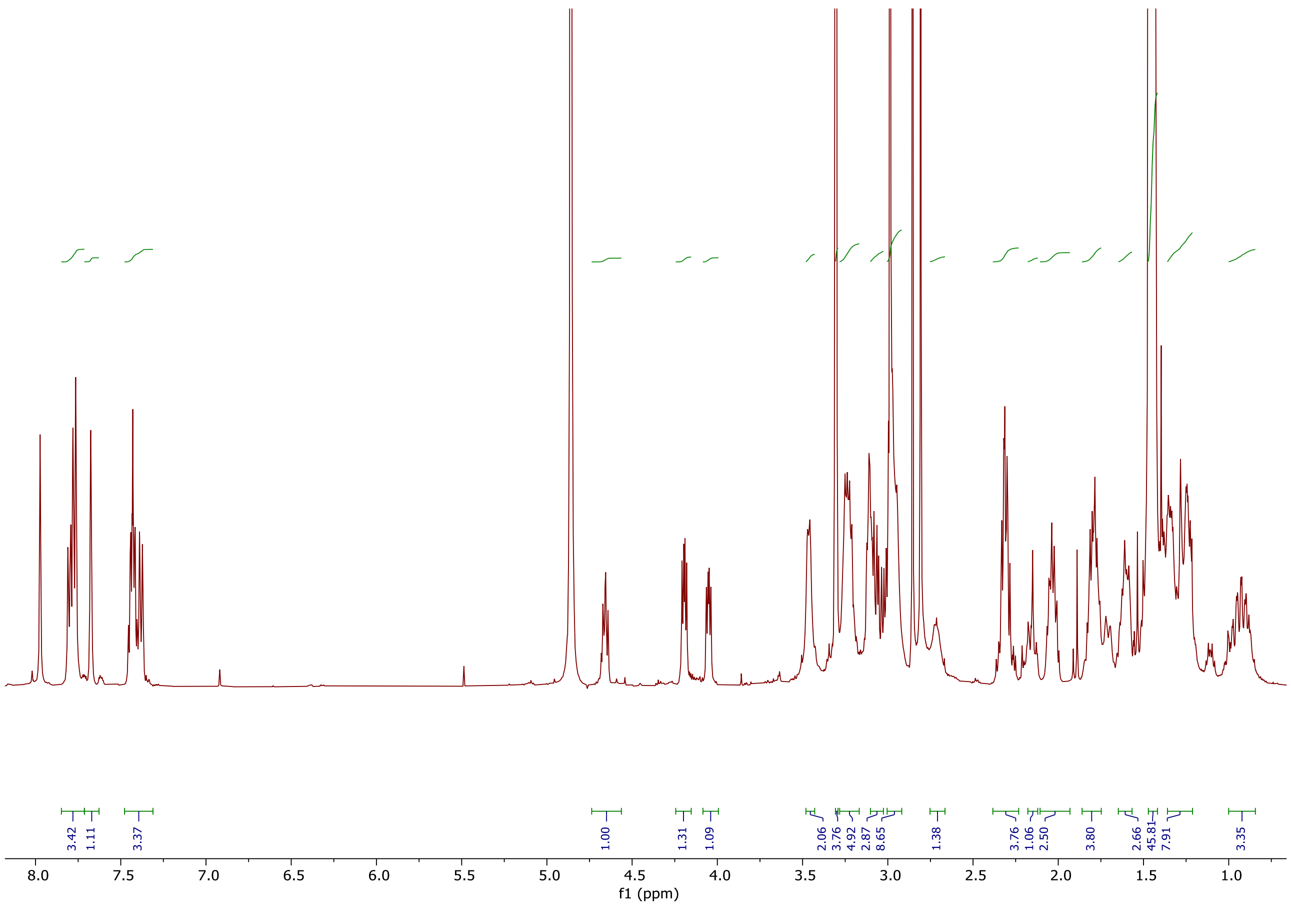

Figure S11: ^{1}H-NMR of Compound **6**

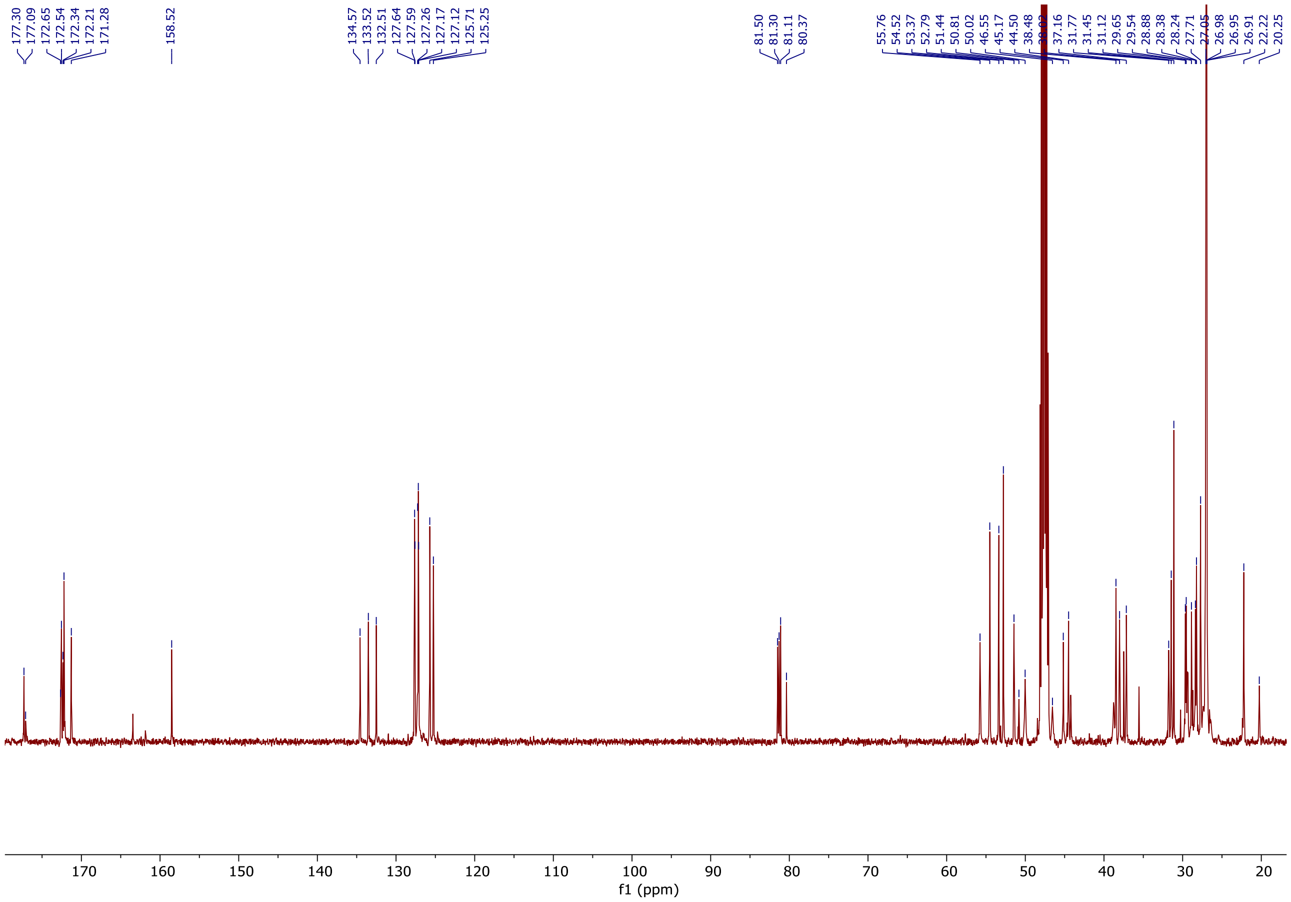

Figure S12: ^{13}C-NMR of Compound **6**

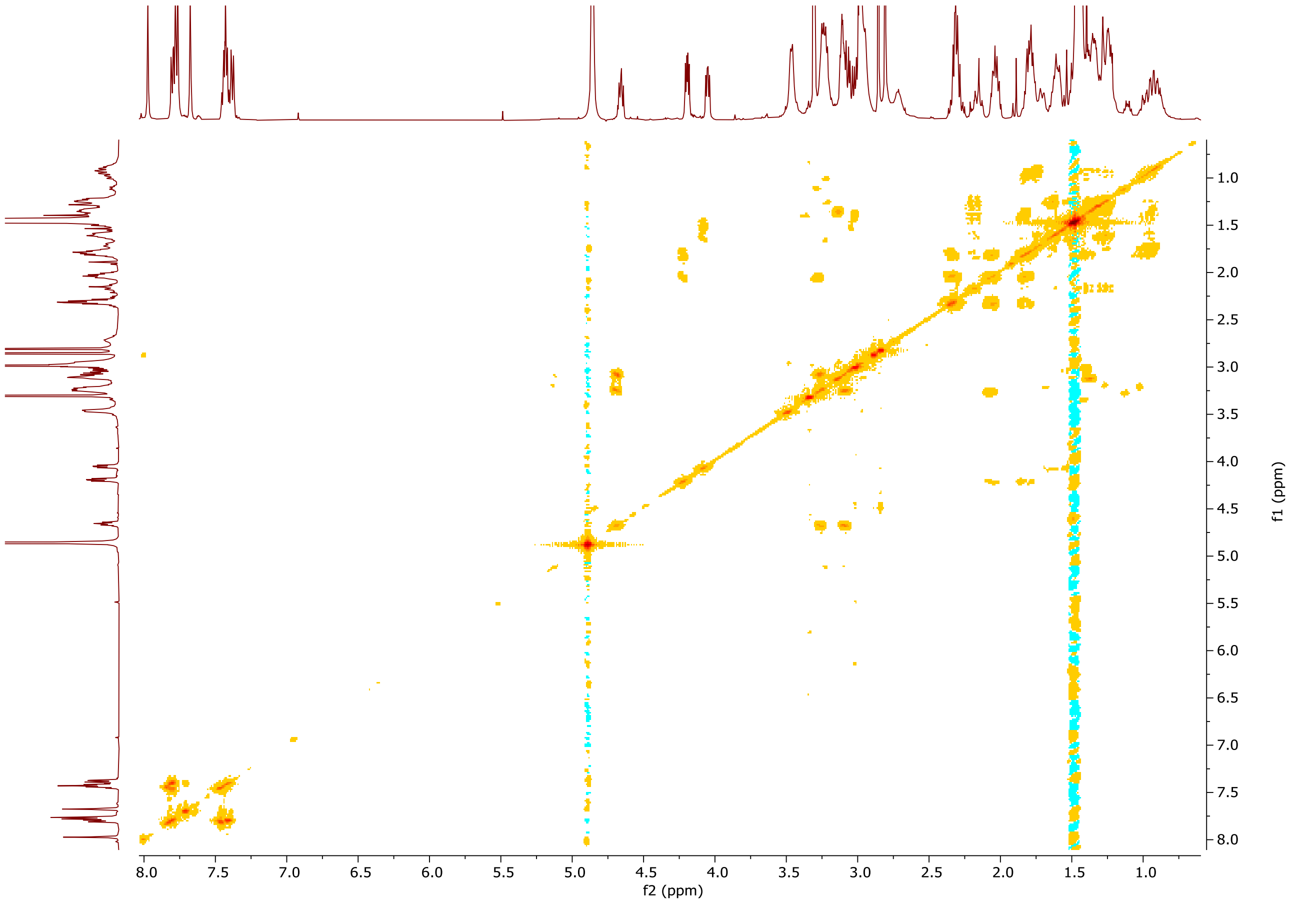

Figure S13: COSY-NMR of Compound **6**

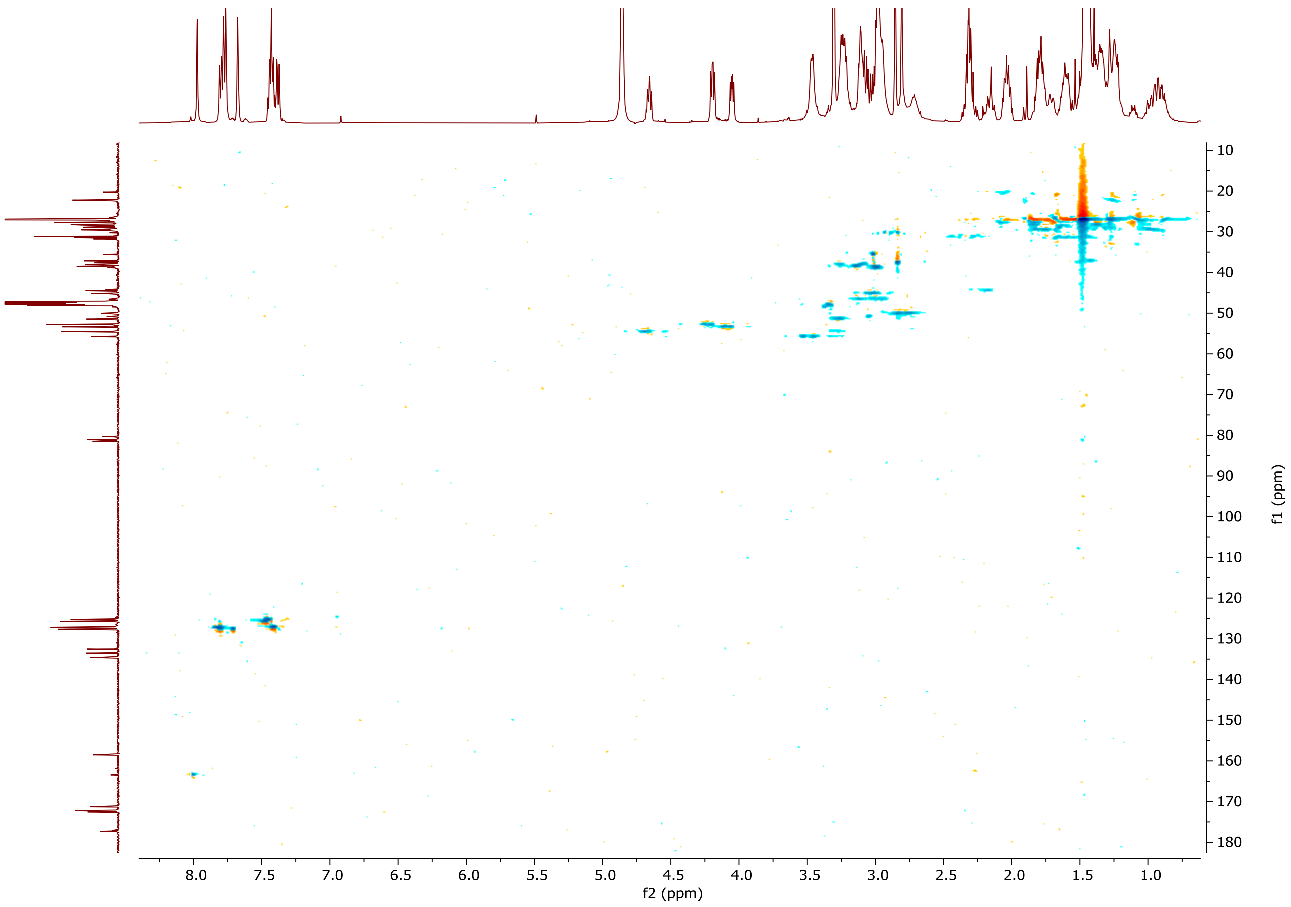

Figure S14: HSQC-NMR of Compound **6**

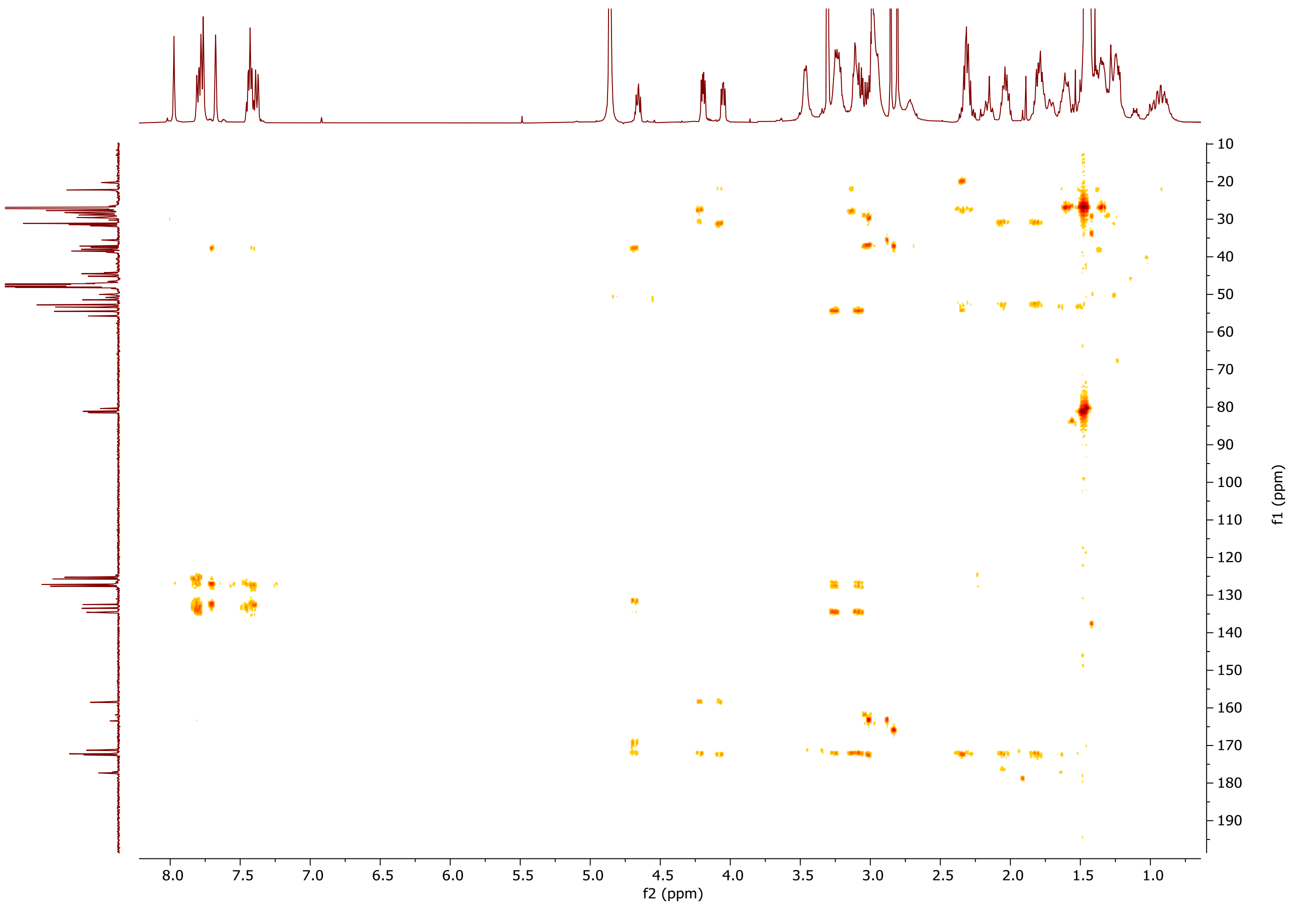

Figure S15: HMBC-NMR of Compound **6**

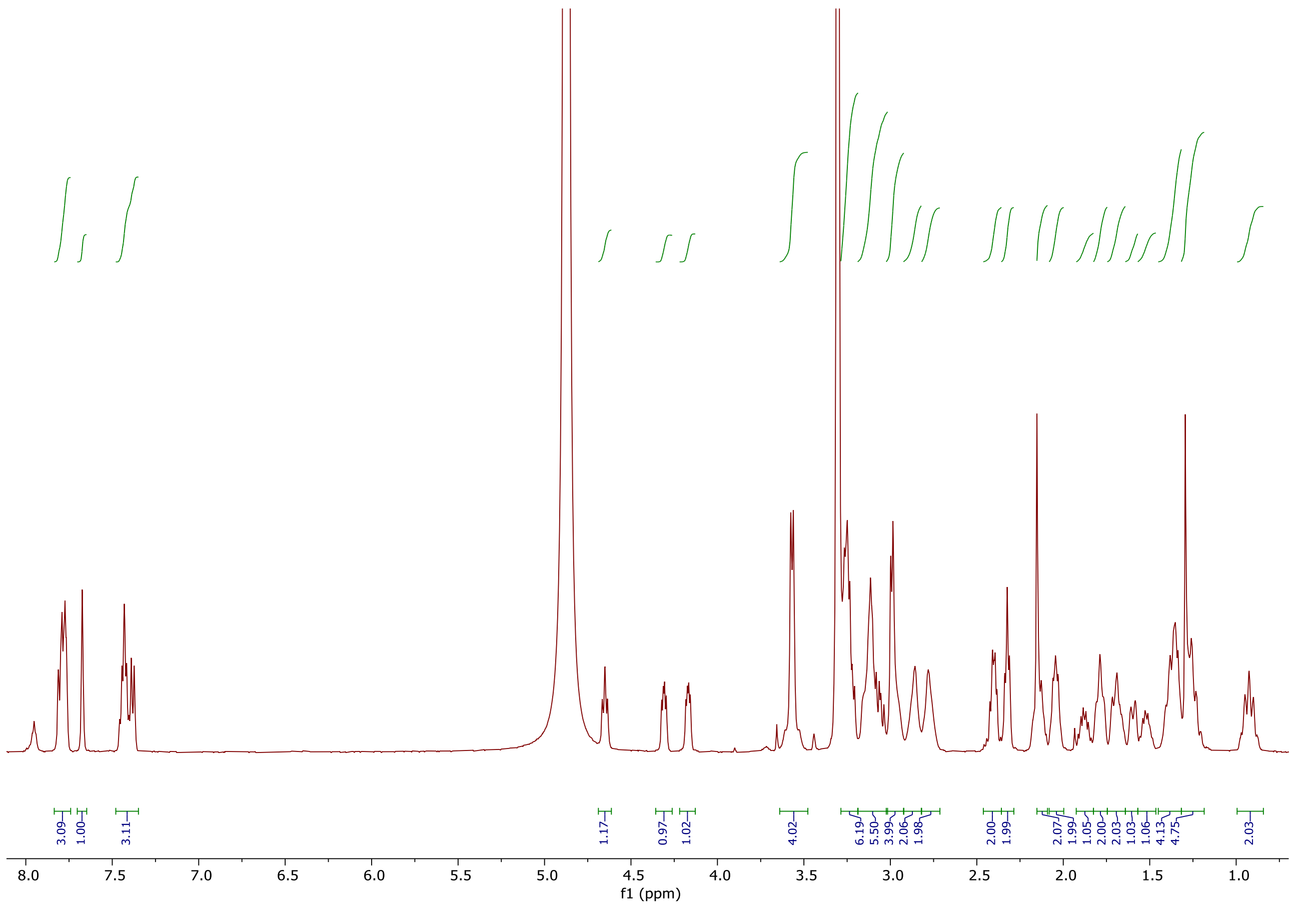

Figure S16: ^{1}H-NMR of Compound **7**

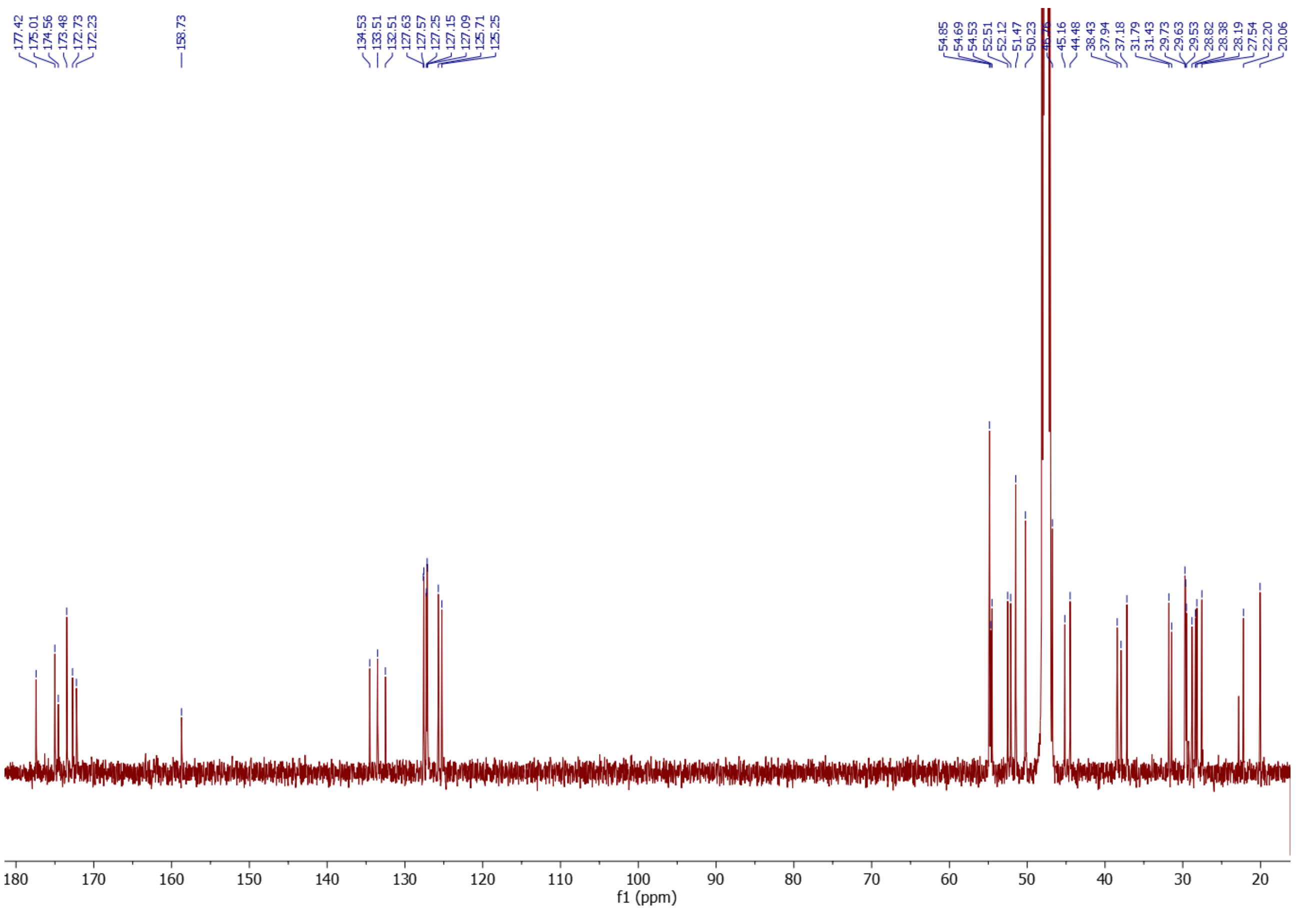

Figure S17: ^{13}C-NMR of Compound **7**

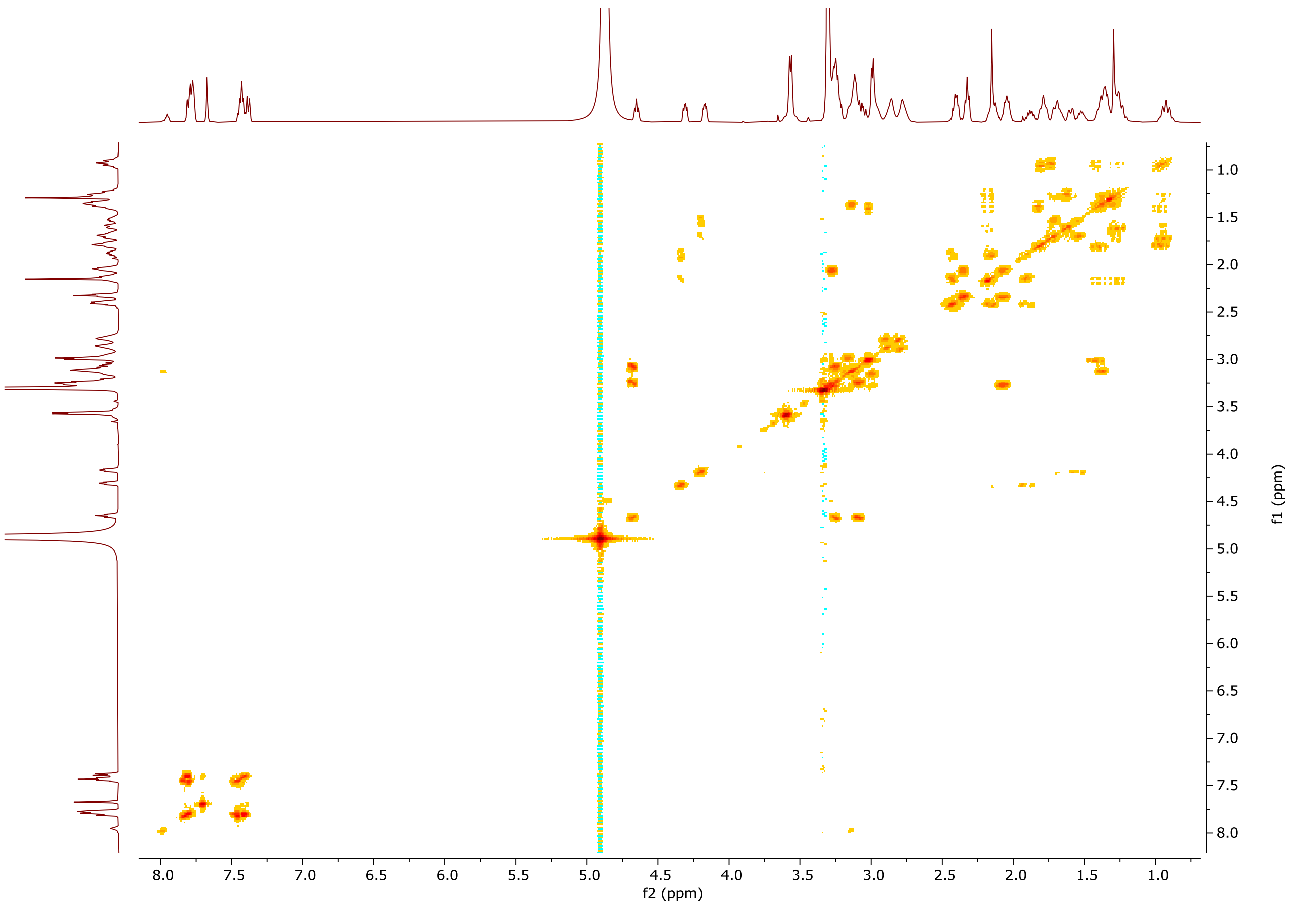

Figure S18: COSY-NMR of Compound **7**

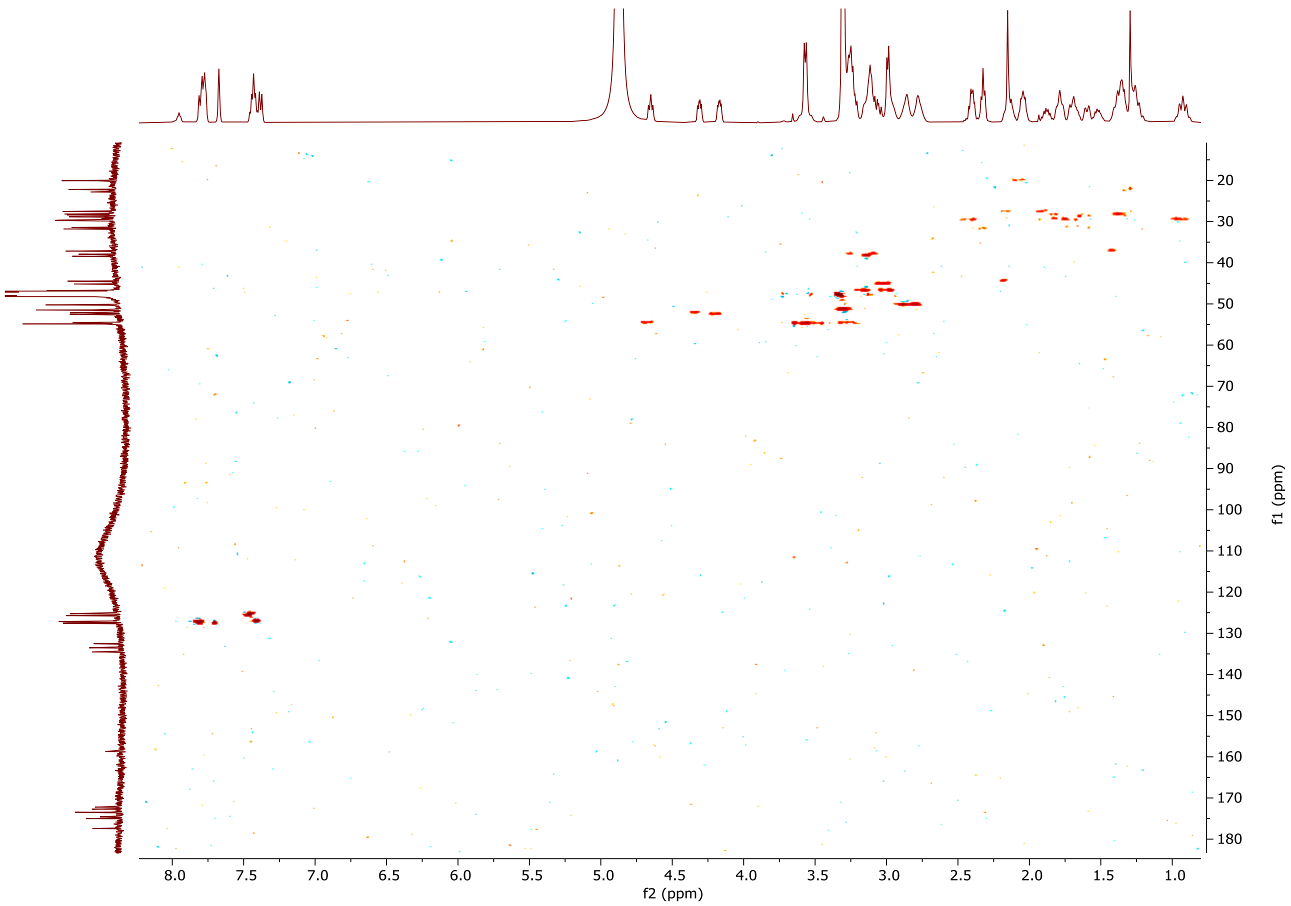

Figure S19: HSQC-NMR of Compound **7**

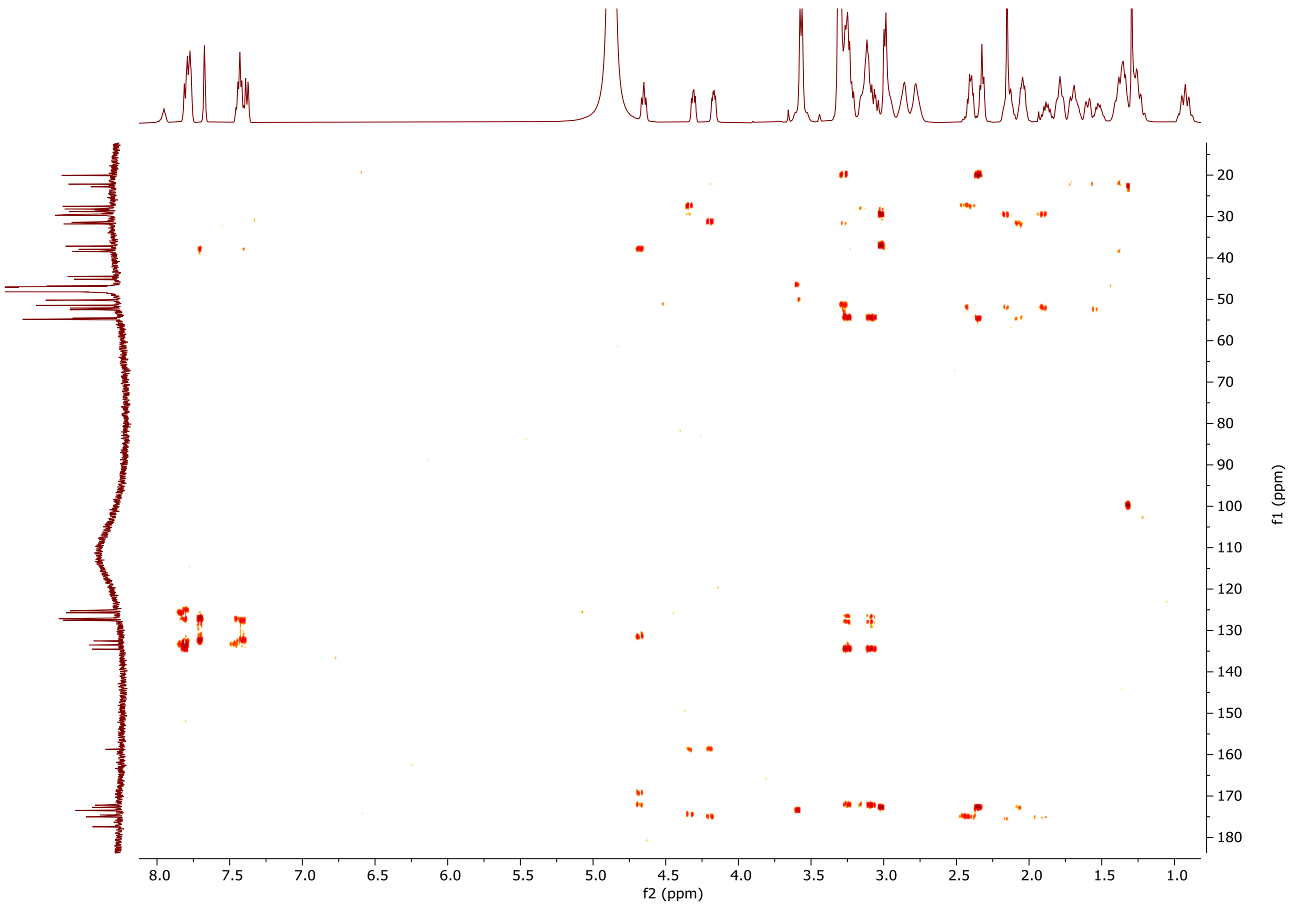

Figure S20: HMBC-NMR of Compound **7**

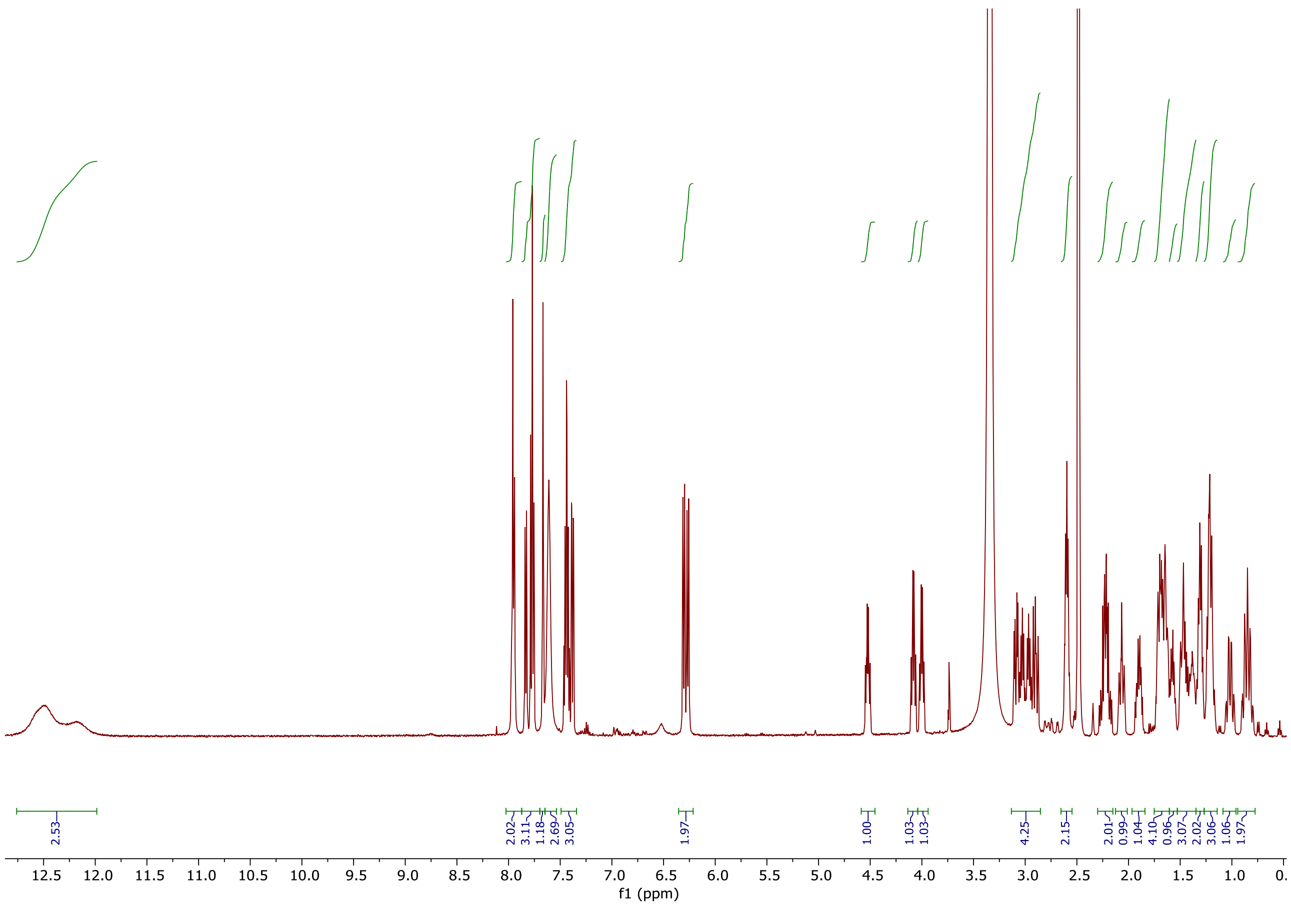

Figure S21: ^{1}H-NMR of Compound **8**

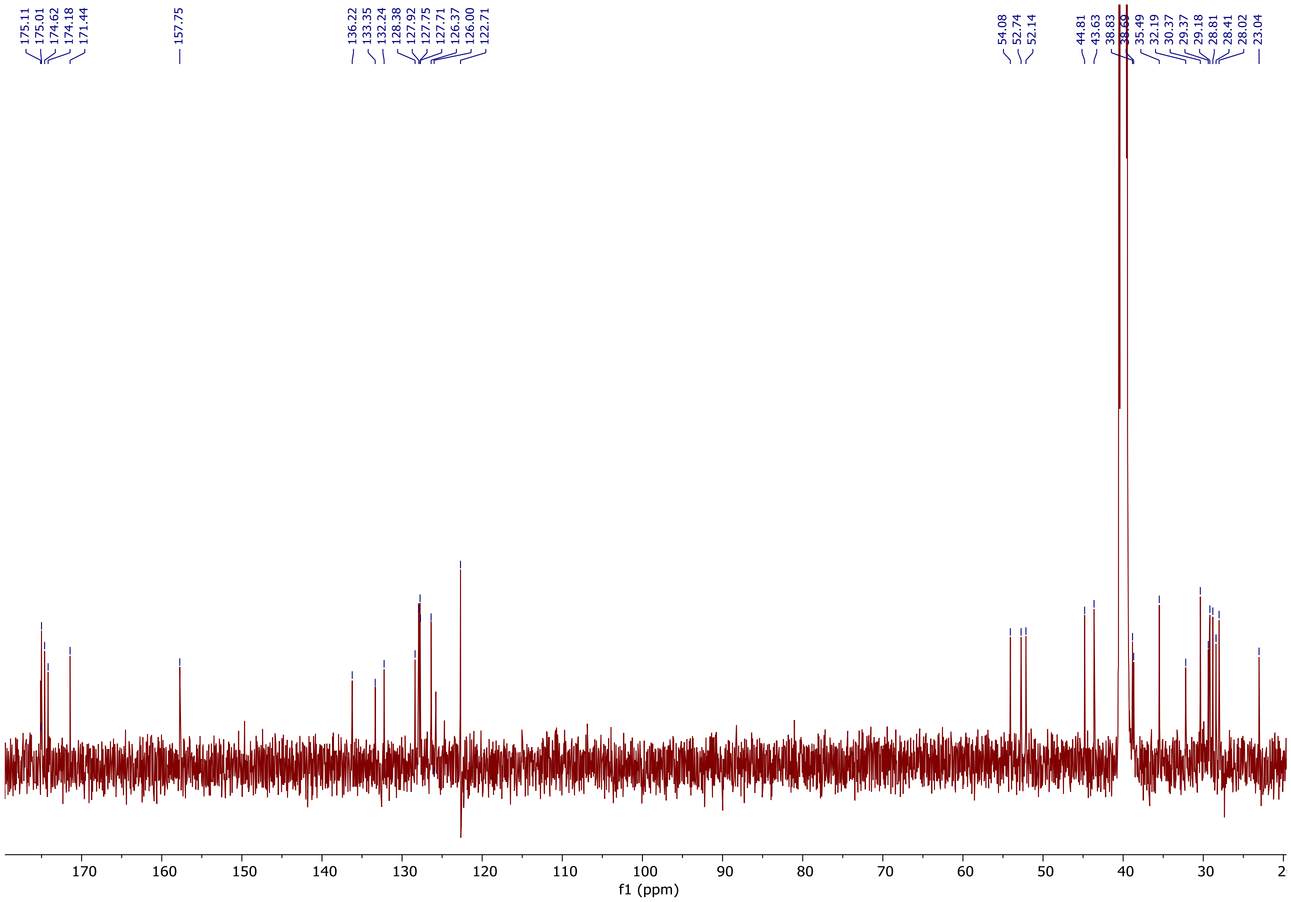

Figure S22: ^{13}C-NMR of Compound **8**

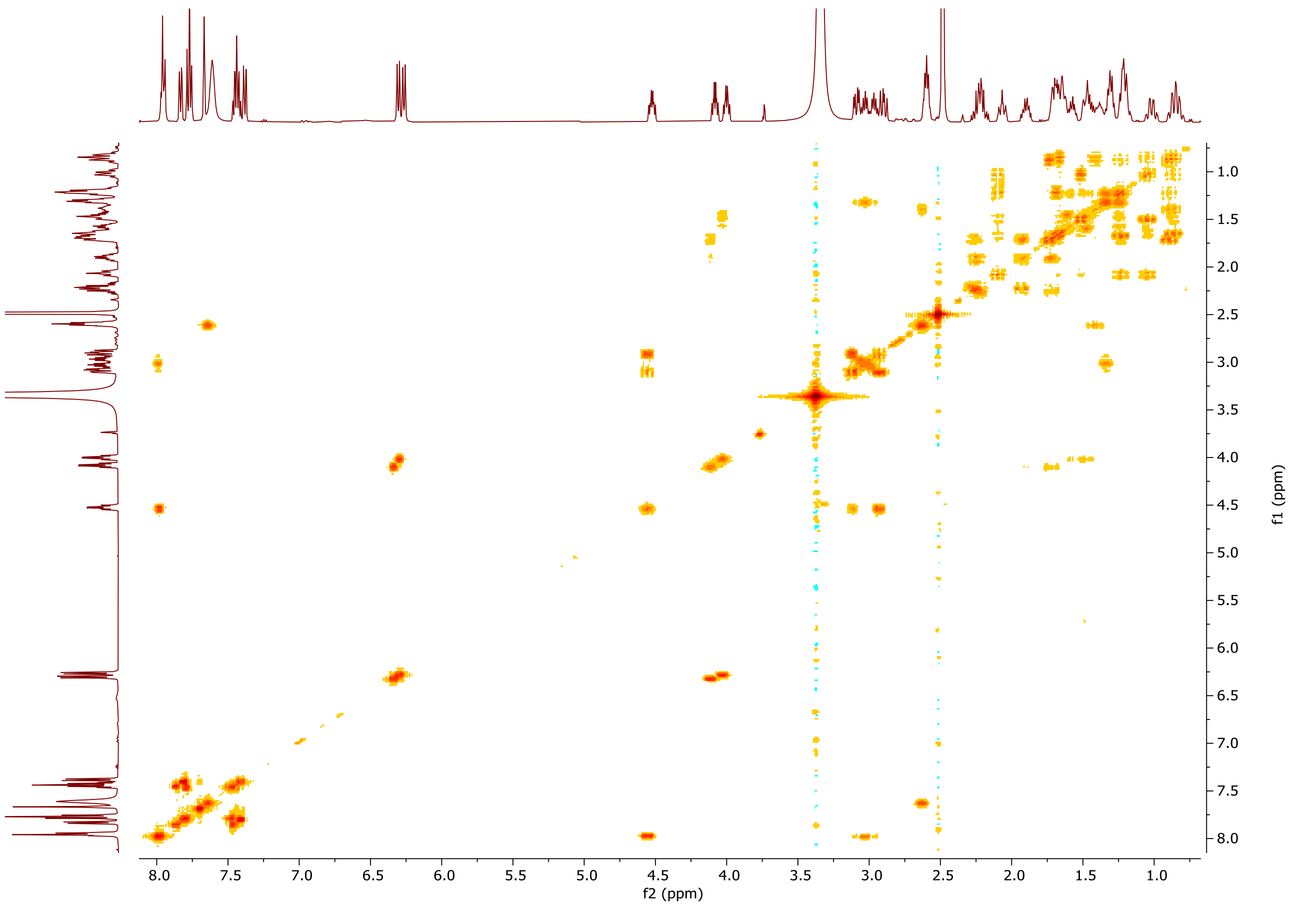

Figure S23: COSY-NMR of Compound **8**

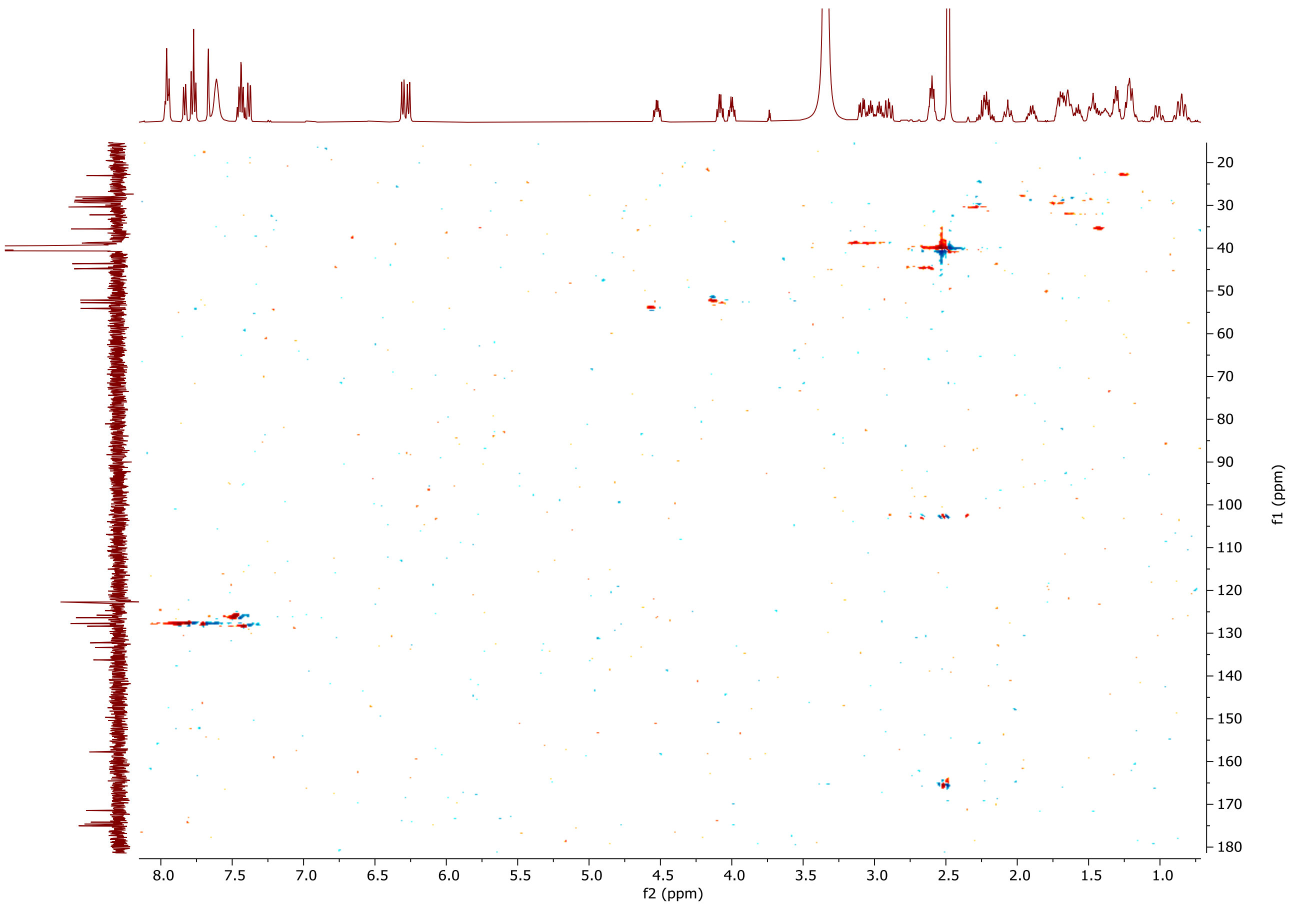

Figure S24: HSQC-NMR of Compound **8**

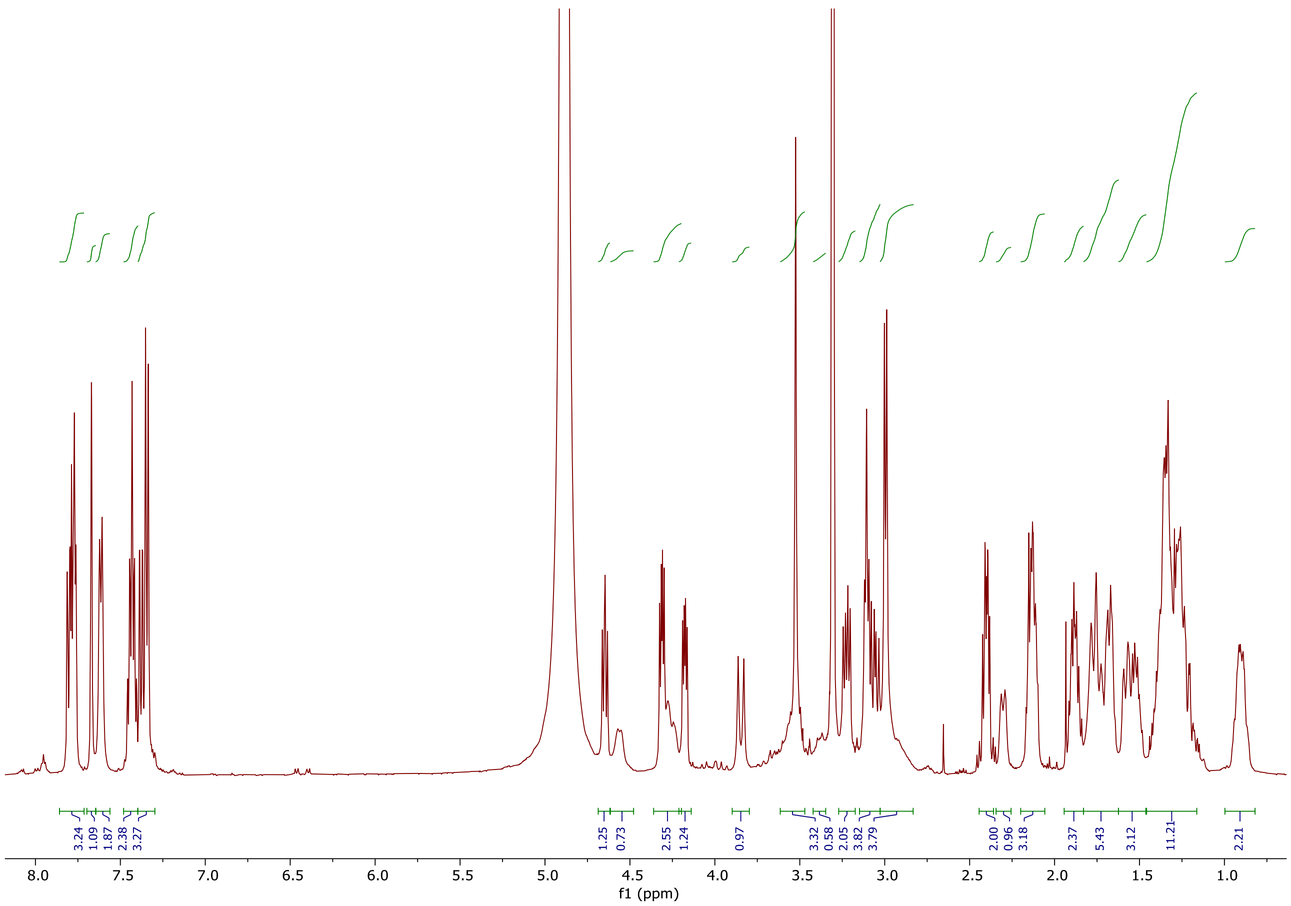

Figure S25: ^{1}H-NMR of Compound **9**

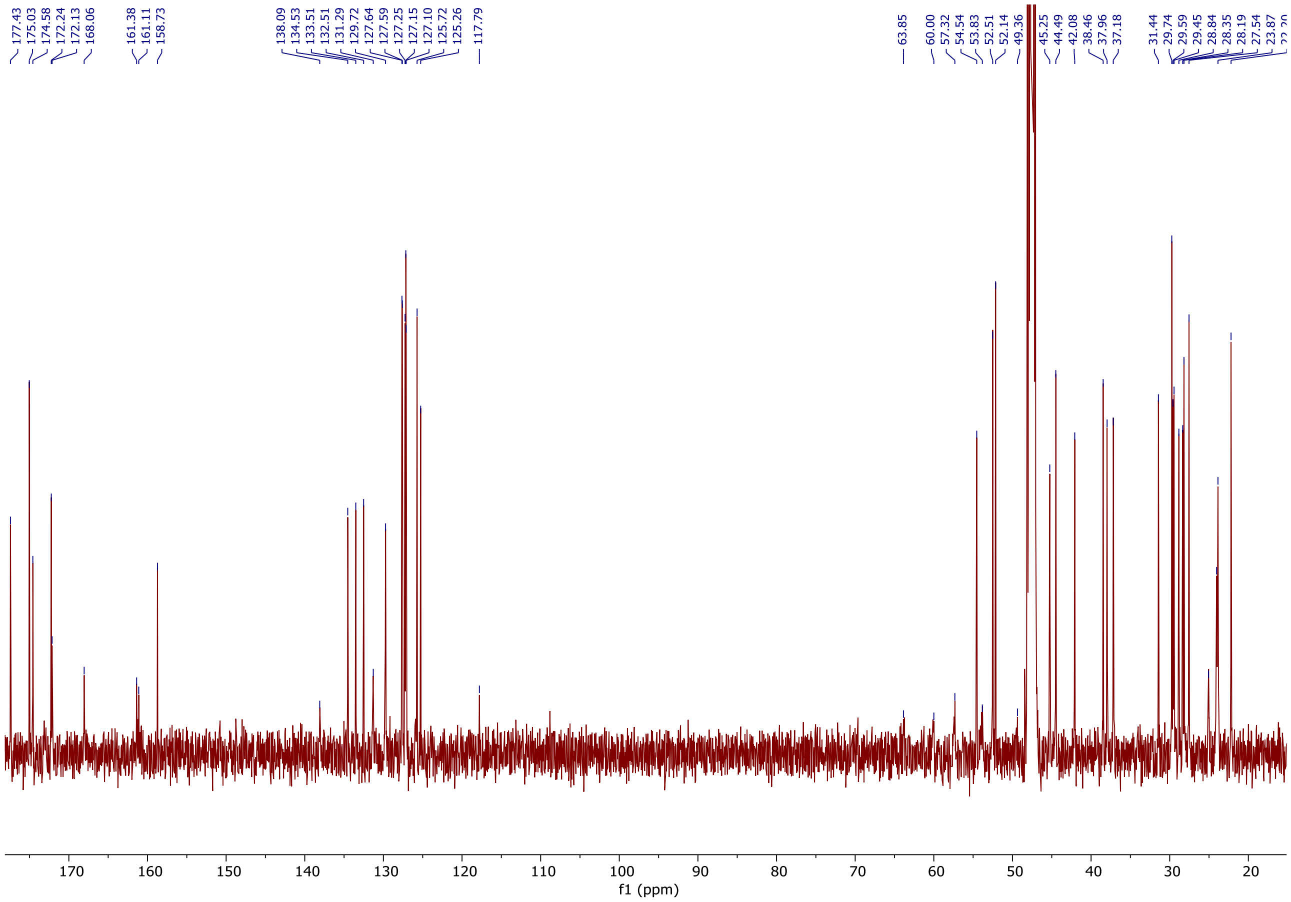

Figure S26: ^{13}C-NMR of Compound **9**

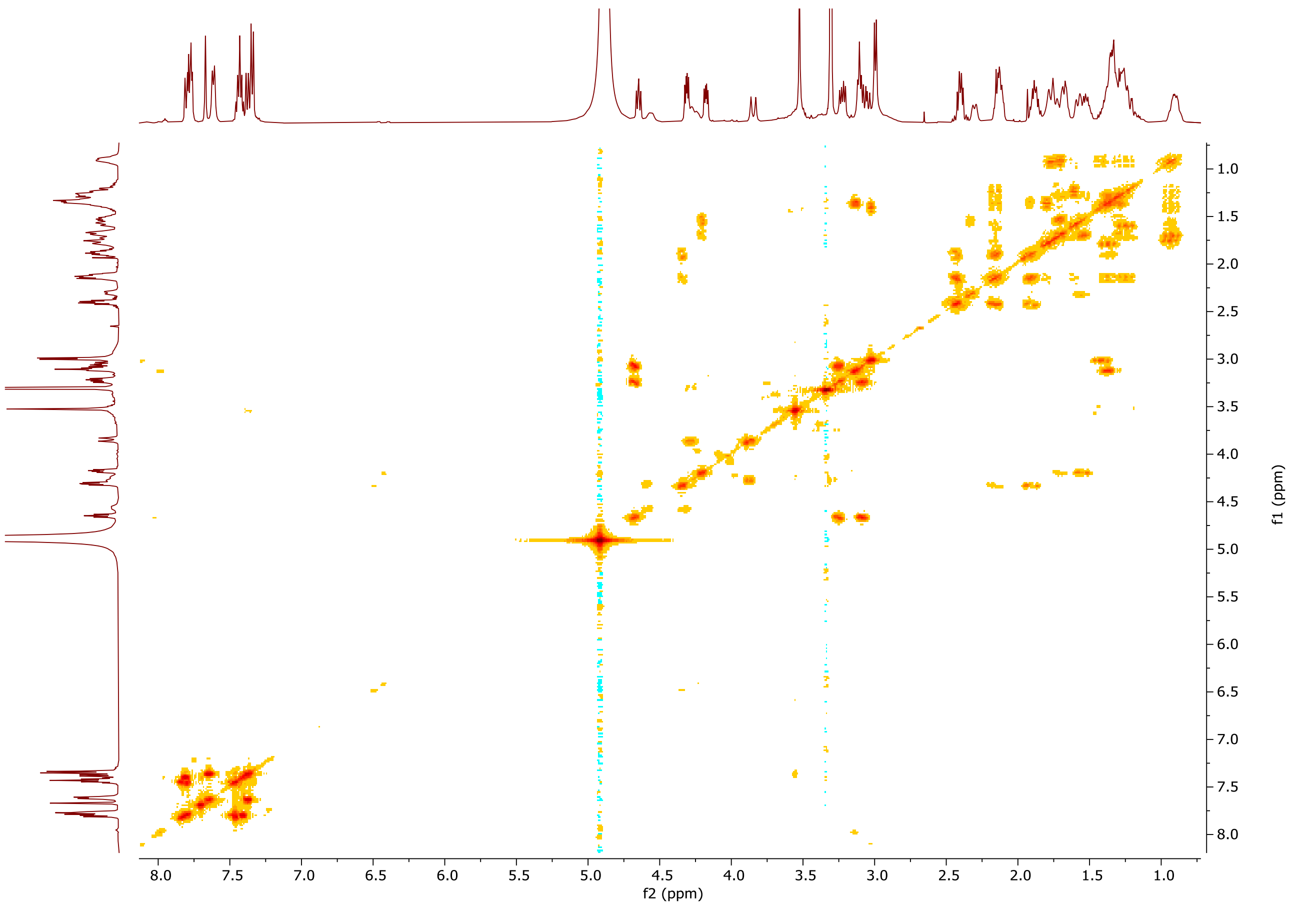

Figure S27: COSY-NMR of Compound **9**

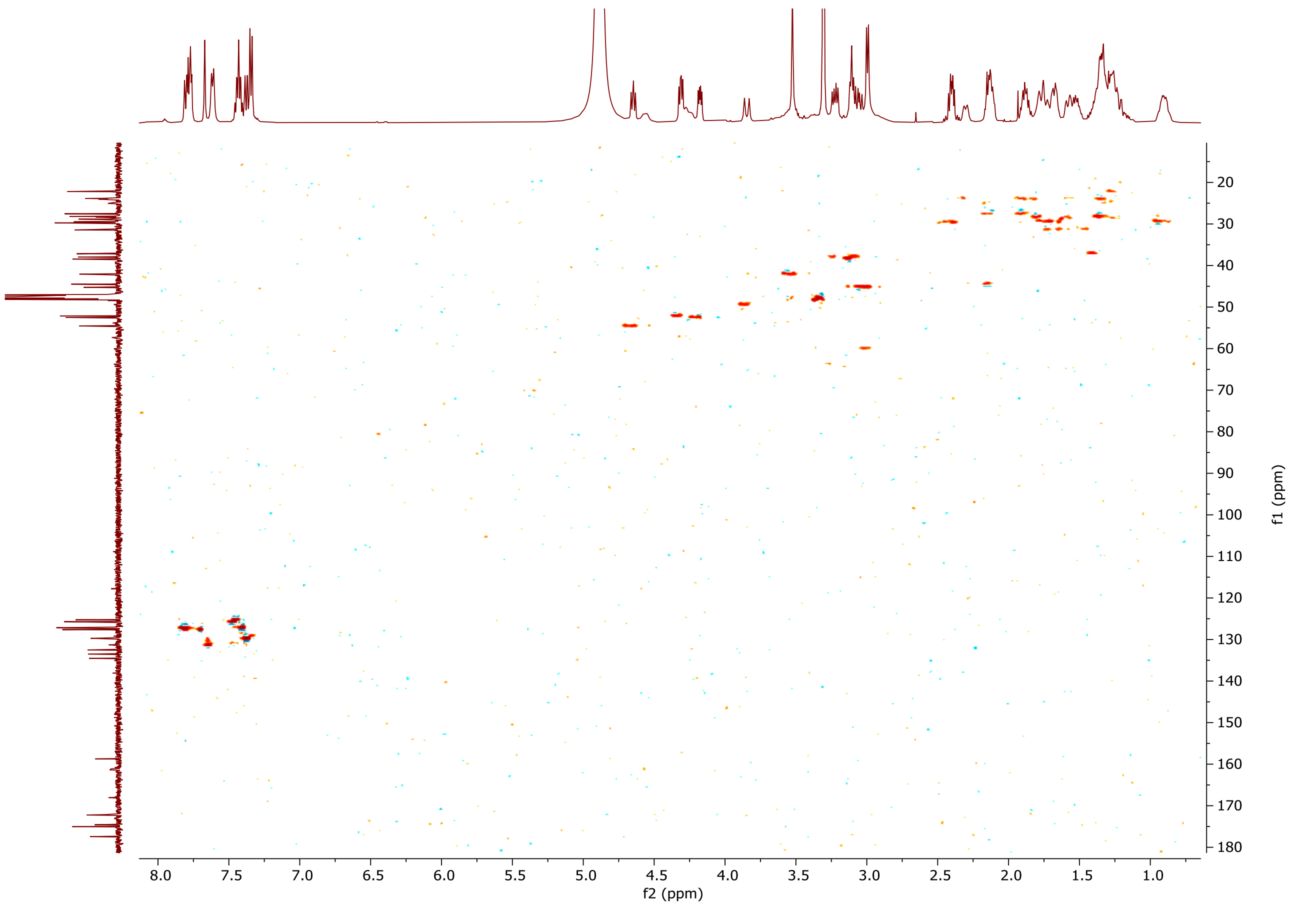

Figure S28: HSQC-NMR of Compound **9**

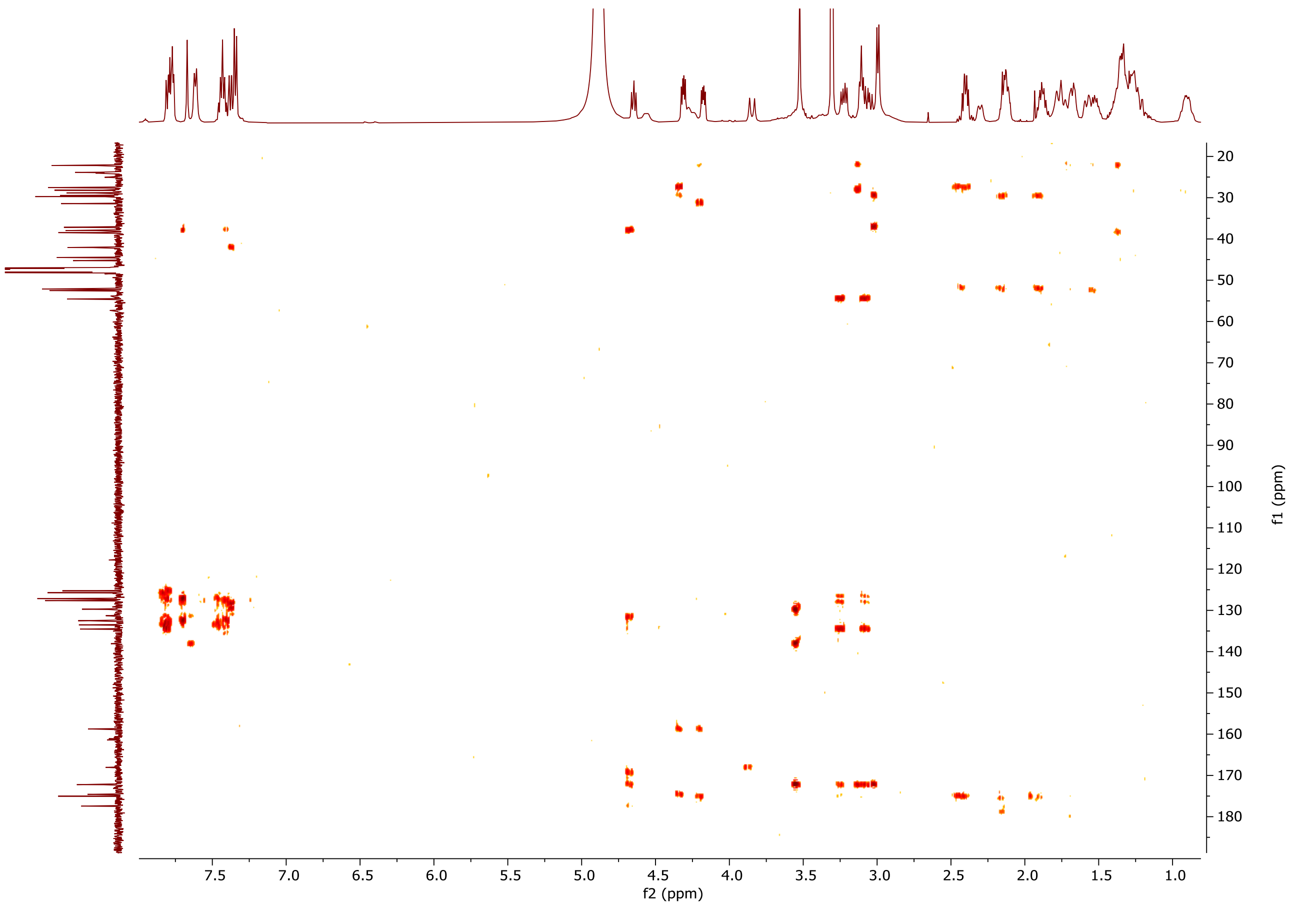

Figure S29: HMBC-NMR of Compound **9**

HPLC CHROMATOGRAMS

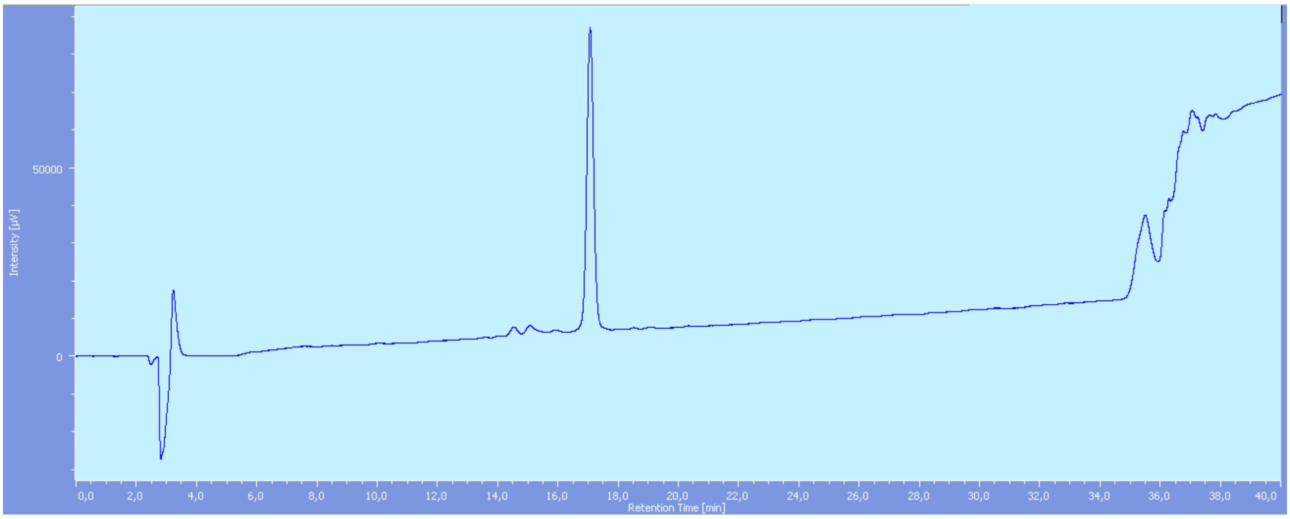

Figure S30: *HPLC chromatogram of product 7*. UV detector: Analytical RP-HPLC condition: column XTerra C18 5µm, 250x4.6 mm; water +0.1%TFA/acetonitrile +0.1%TFA isocratic at 80:20 for 1 min, gradient from 80:20 to 60:40 in 30 min, gradient from 60:40 to 20:80 in 2 min; 1 ml/min, 220 nm, UV detector. R_t: 17.2 min.

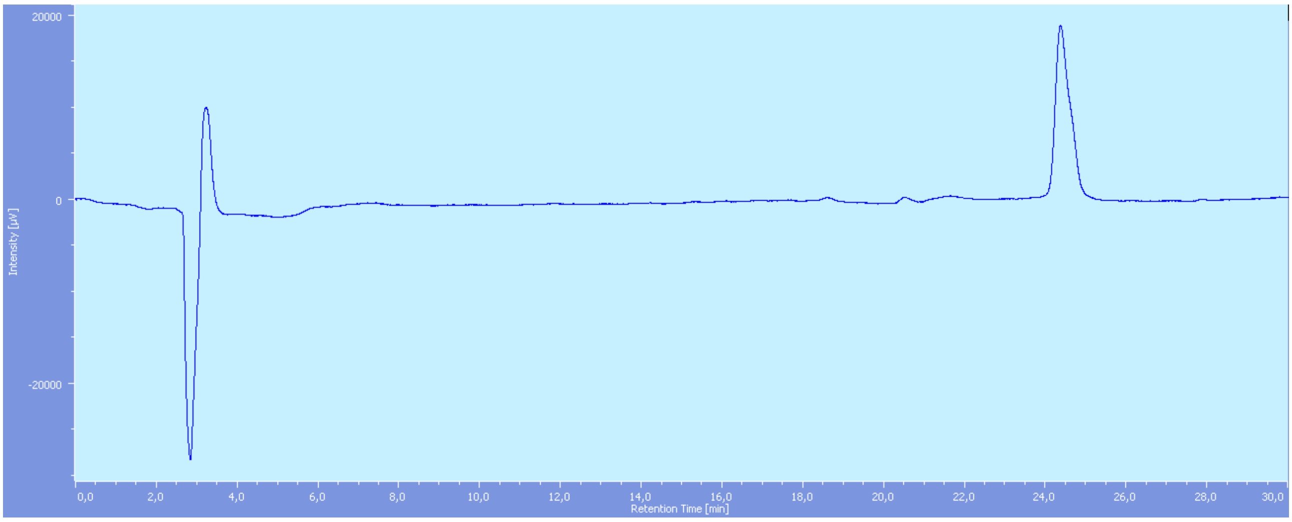

Figure S31: *HPLC chromatogram of product **9***. UV detector: Analytical RP-HPLC condition: column XTerra C18 5µm, 250x4.6 mm; water +0.1%TFA/acetonitrile +0.1%TFA isocratic at 80:20 for 1 min, gradient from 80:20 to 60:40 in 30 min; 1 ml/min, 220 nm, UV detector. R_t: 24.7 min.

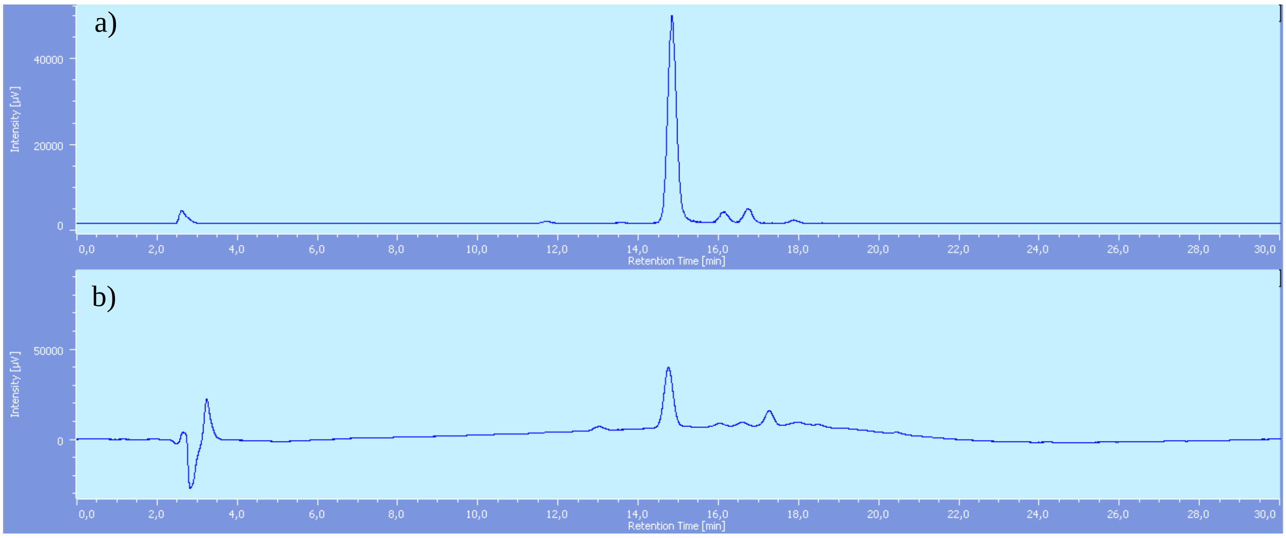

Figure S32: *HPLC chromatogram of crude [^{18}F]1*. a) Radiochemical detector; b) UV detector. Analytical RP-HPLC condition: column XTerra C18 5µm, 250x4.6 mm; water +0.1%TFA/acetonitrile +0.1%TFA isocratic at 80:20 for 1 min, gradient from 80:20 to 60:40 in 30 min, gradient from 60:40 to 20:80 in 2 min; 1 ml/min, 220 nm, UV detector. R_t **[^{18}F] 1**: 14.9 min, Rt precursor **7**: 17.2 min.

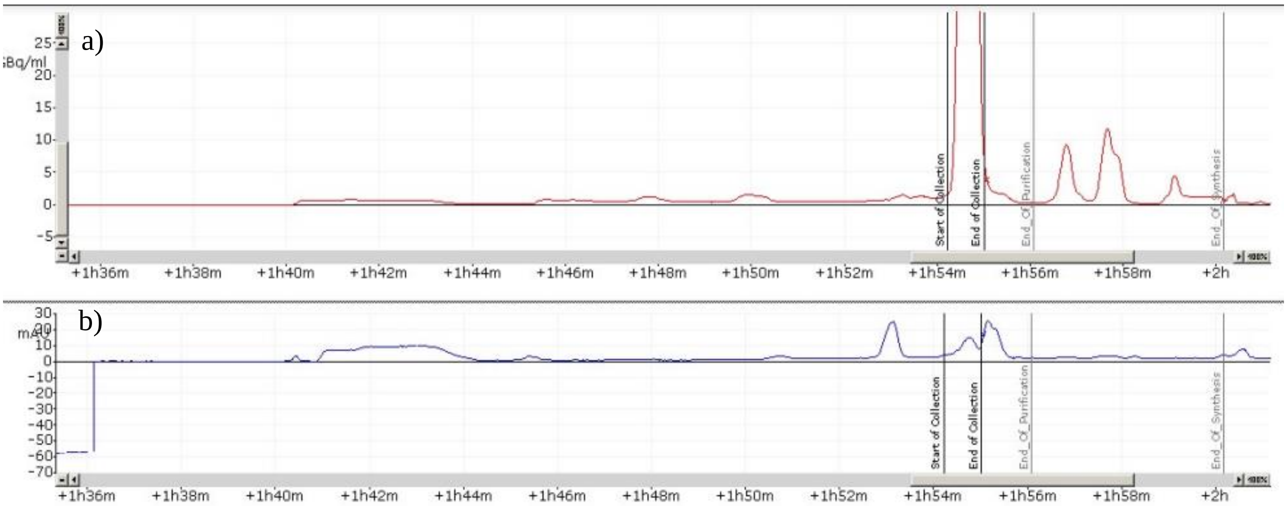

Figure S33: *Semi-preparative RP-HPLC chromatogram of crude [^{18}F]1*. a) Radiochemical detector; b) UV detector. Semi-preparative RP-HPLC condition: column Clarity Oligo-RP 5µm, 250x10 mm; water +0.1%TFA/acetonitrile +0.1%TFA gradient from 80:20 to 60:40 in 30 min; 5 ml/min, 220 nm, UV detector. R_t: 20 min

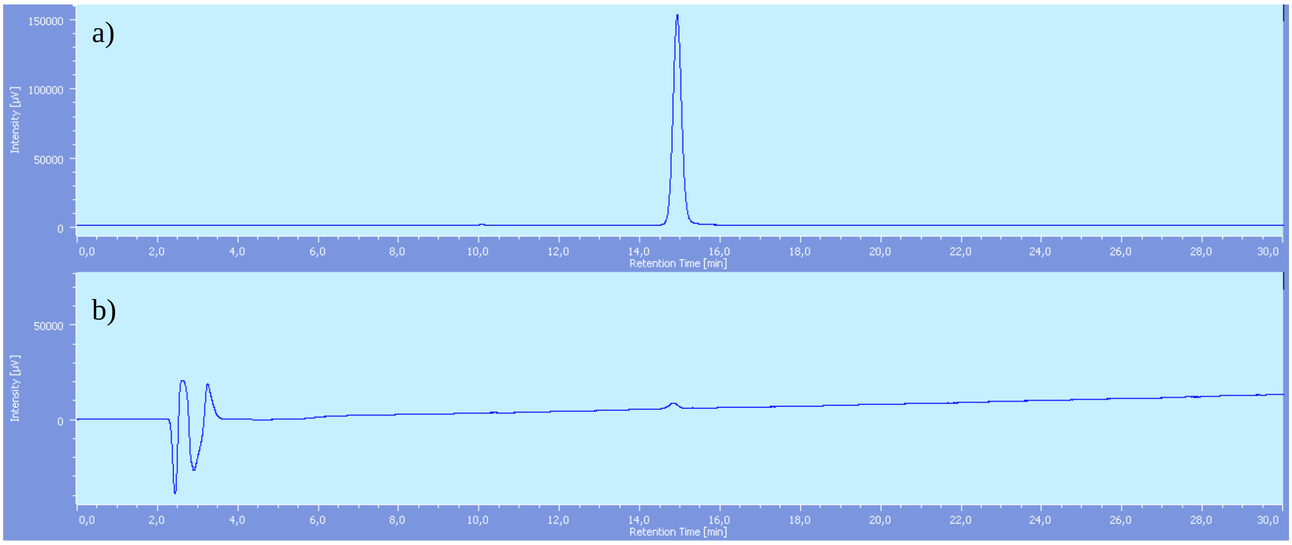

Figure S34: *HPLC chromatogram of pure [^{18}F]1*. a) Radiochemical detector; b) UV detector. Analytical RP-HPLC condition: column XTerra C18 5µm, 250x4.6 mm; water +0.1%TFA/acetonitrile +0.1%TFA isocratic at 80:20 for 1 min, gradient from 80:20 to 60:40 in 30 min, gradient from 60:40 to 20:80 in 2 min; 1 ml/min, 220 nm, UV detector. R_t **[^{18}F]1**: 14.9 min

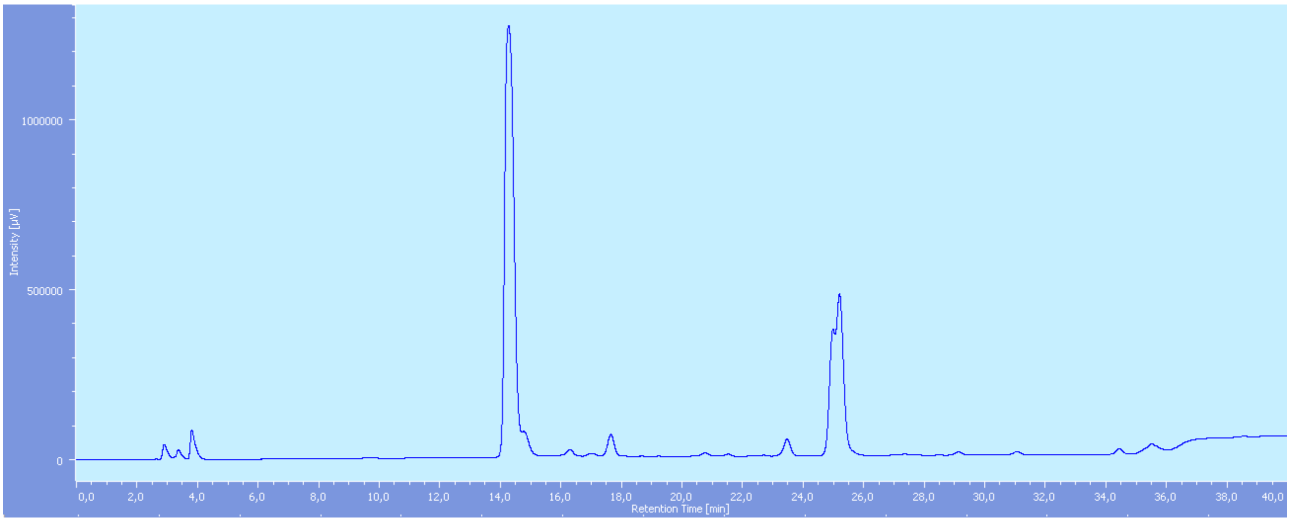

Figure S35: *HPLC chromatogram of crude **8***. UV detector, Analytical RP-HPLC condition: column XTerra C18 5µm, 250x4.6 mm; water +0.1%TFA/acetonitrile +0.1%TFA isocratic at 80:20 for 1 min, gradient from 80:20 to 60:40 in 30 min; 1 ml/min, 220 nm, UV detector. R_t: 14.3 min

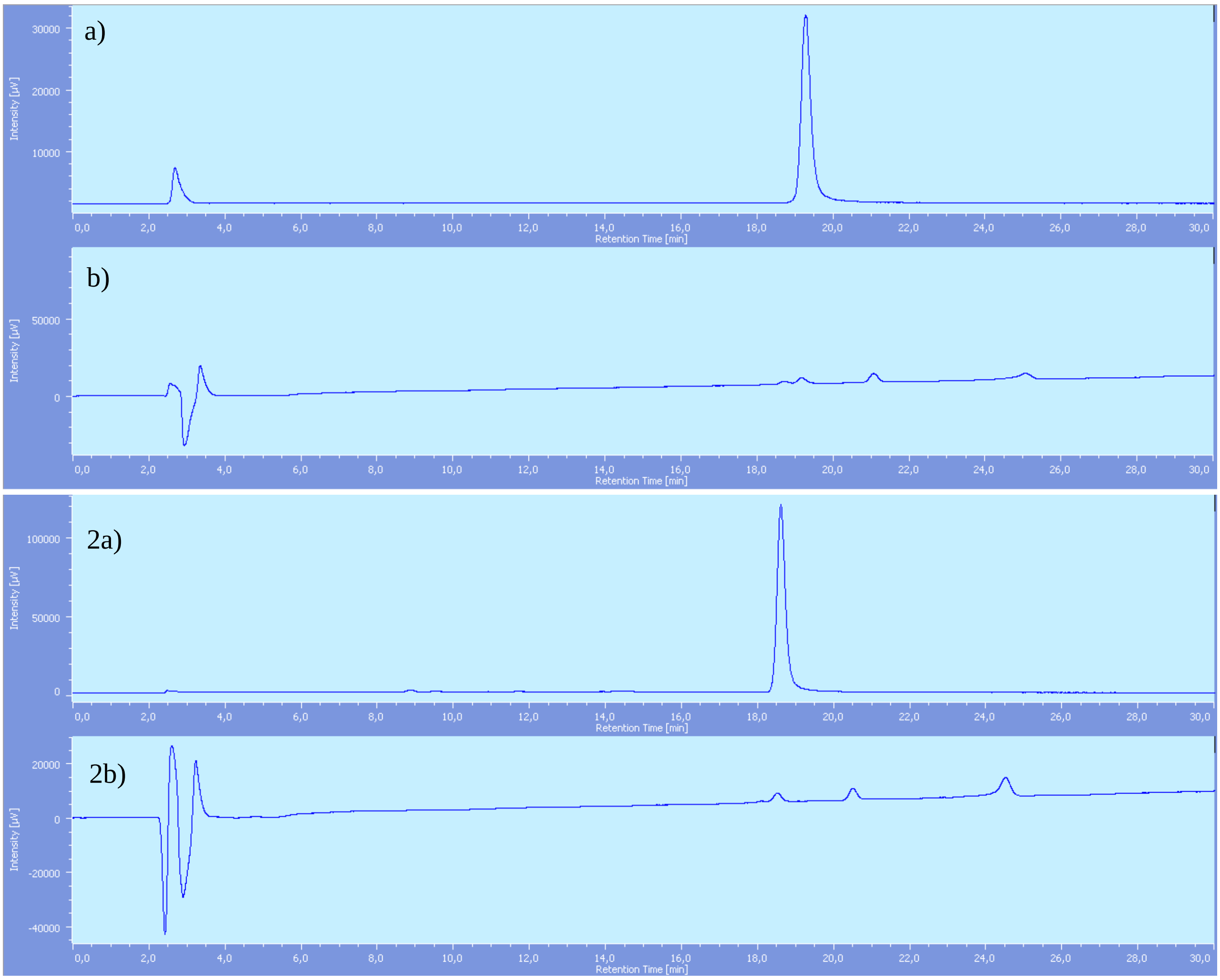

Figure S36: *HPLC chromatogram of [^18^F]2*. a) Radiochemical detector before purification; b) UV detector before purification; 2a) Radiochemical detector after purification; 2b) UV detector after purification. Analytical RP-HPLC condition: column XTerra C18 5µm, 250x4.6 mm; water +0.1%TFA/acetonitrile +0.1%TFA isocratic at 80:20 for 1 min, gradient from 80:20 to 60:40 in 30 min, gradient from 60:40 to 20:80 in 2 min; 1 ml/min, 220 nm, UV detector. Rt [^{18}F] AlF^{2+}: 2.5 min, Rt product **[^{18}F] 2**: 18.6 min, Rt PSMA-617-RESCA complexed with $[Al(OH)]^{2+}$: 20.7 min, Rt precursor **9**: 24.7 min.

CHROMATOGRAMS AND RADIO-TLC FOR IN VITRO STABILITY STUDIES

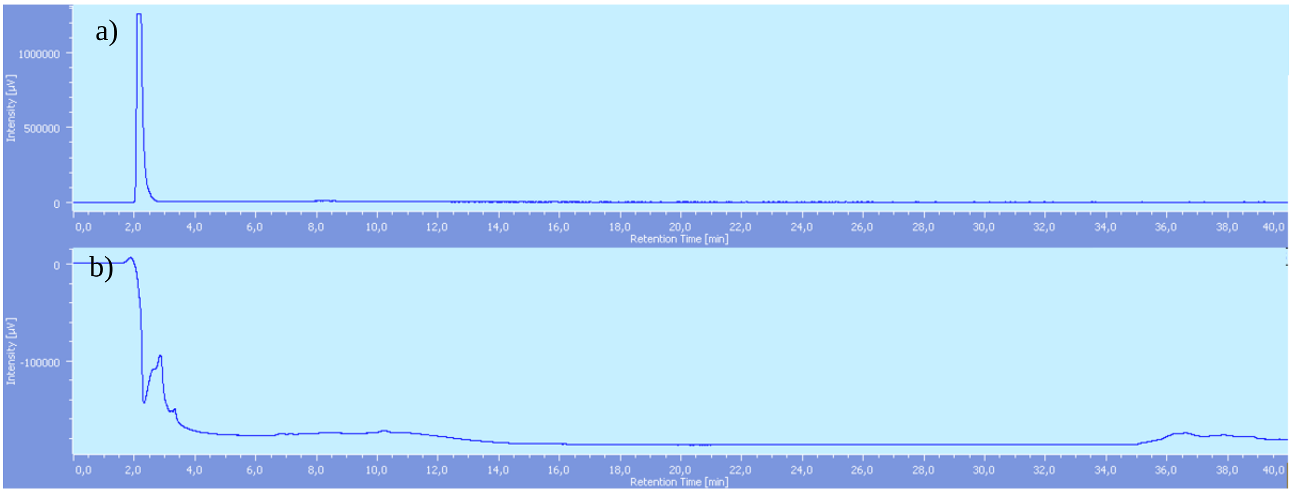

Figure S37: *HPLC chromatogram of [^{18}F]AlF^{2+}*. a) Radiochemical detector; b) UV detector. Analytical RP-HPLC condition: column XTerra C18 5µm, 250x4.6 mm; water +0.1%TFA/acetonitrile +0.1%TFA isocratic at 80:20 for 1 min, gradient from 80:20 to 60:40 in 30 min, gradient from 60:40 to 20:80 in 2 min; 1 ml/min, 220 nm, UV detector. Rt [^{18}F] AlF^{2+}: 2.5 min. Injected activity: ~5 MBq

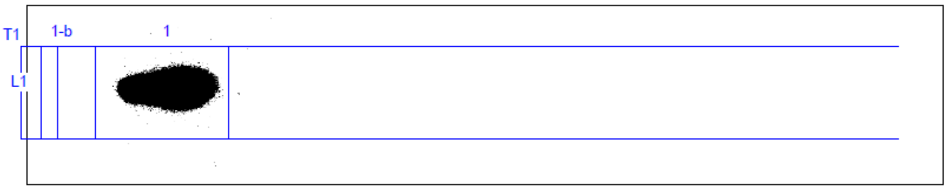

Lane #1
Background Subtraction: Regions = 352.579 DLU /mm2

ID	Dist From Origin(mm)	RF	Gross DLU	Background Subtract	Net DLU	Net % Sum	Net % Max Reg.
1 - Prof	16.8	0.0	15,839,014.3	53,468.8	15,785,545.5	100.0	100.0
1-b - Prof			6,919.5				
Lane			16,066,269.3	352,579.4	15,713,689.8		
UnRes			227,255.0	299,110.6	-71,855.7		

Figure S38: *Radio-TLC of [^{18}F]AlF^{2+}*. Eluent ammonium acetate 0.05M, pH 5.5 / ACN 1:1. Injected activity: 7.5 kBq

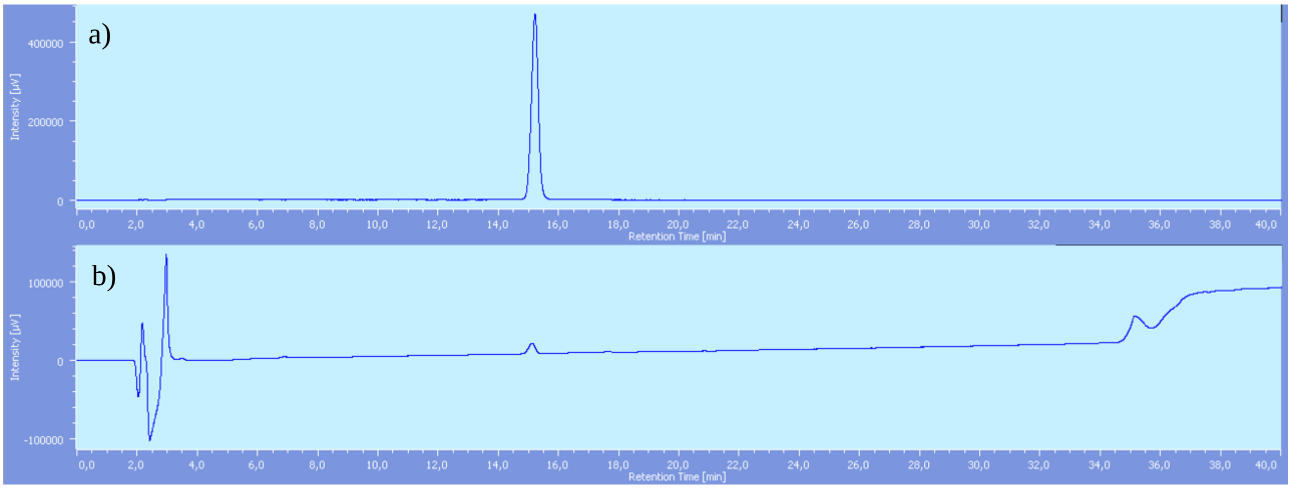

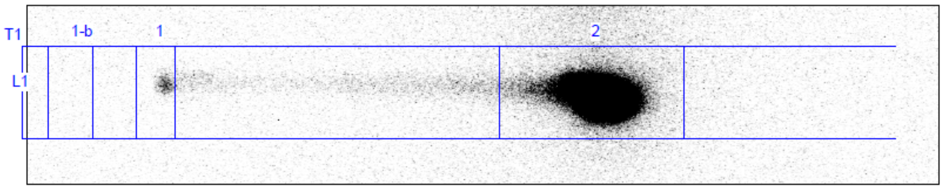

Lane #1
Background Subtraction: Baseline

ID	Dist From Origin(mm)	RF	Gross DLU	Background Subtract	Net DLU	Net % Sum	Net % Max Reg.
1 - Prof	15.9	0.0	41,354.5	10,383.9	30,970.6	0.2	0.2
2 - Prof	66.7	0.7	20,266,044.3	50,224.3	20,215,820.0	99.8	100.0
1-b - Prof			12,079.3				
Lane			20,697,798.7	237,559.0	20,460,239.7		
UnRes			390,399.8	176,950.7	213,449.1		

Figure S39: *HPLC chromatogram and Radio-TLC of [^{18}F]**1** at t=0 in solution*. HPLC: a) Radiochemical detector; b) UV detector. Analytical RP-HPLC condition: column XTerra C18 5µm, 250x4.6 mm; water +0.1%TFA/acetonitrile +0.1%TFA isocratic at 80:20 for 1 min, gradient from 80:20 to 60:40 in 30 min, gradient from 60:40 to 20:80 in 2 min; 1 ml/min, 220 nm, UV detector. R_t **[^{18}F]1**: 14.9 min. Injected activity: ~18 MBq. TLC: Eluent ammonium acetate 0.05M, pH 5.5 / ACN 1:1. Injected activity: 7.5 kBq.

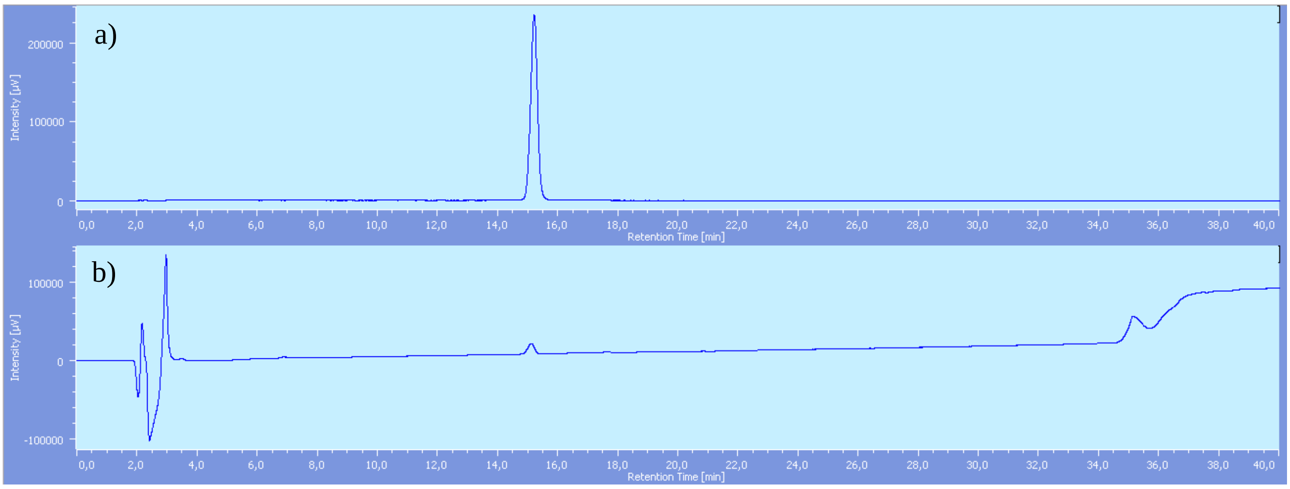

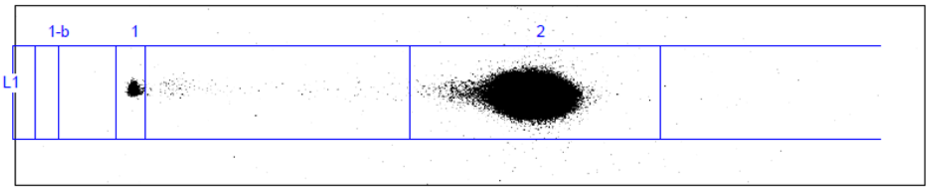

Lane #1
Background Subtraction: Regions = 272.356 DLU /mm2

ID	Dist From Origin(mm)	RF	Gross DLU	Background Subtract	Net DLU	Net % Sum	Net % Max Reg.
**1 - Prof**	13.6	0.0	96,942.2	9,232.4	87,709.8	0.5	0.5
**2 - Prof**	60.3	0.6	19,080,163.1	78,475.3	19,001,687.8	99.5	100.0
**1-b - Prof**			7,531.7				
**Lane**			19,531,280.7	272,355.6	19,258,925.1		
**UnRes**			354,175.4	184,647.8	169,527.5		

Figure S40: *HPLC chromatogram and Radio-TLC of **[^{18}F]1** at t=2h in solution*. HPLC: a) Radiochemical detector; b) UV detector. Analytical RP-HPLC condition: column XTerra C18 5µm, 250x4.6 mm; water +0.1%TFA/acetonitrile +0.1%TFA isocratic at 80:20 for 1 min, gradient from 80:20 to 60:40 in 30 min, gradient from 60:40 to 20:80 in 2 min; 1 ml/min, 220 nm, UV detector. R_t **[^{18}F]1**: 14.9 min. Injected activity: ~9 MBq. TLC: Eluent ammonium acetate 0.05M, pH 5.5 / ACN 1:1. Injected activity: 7.5 kBq.

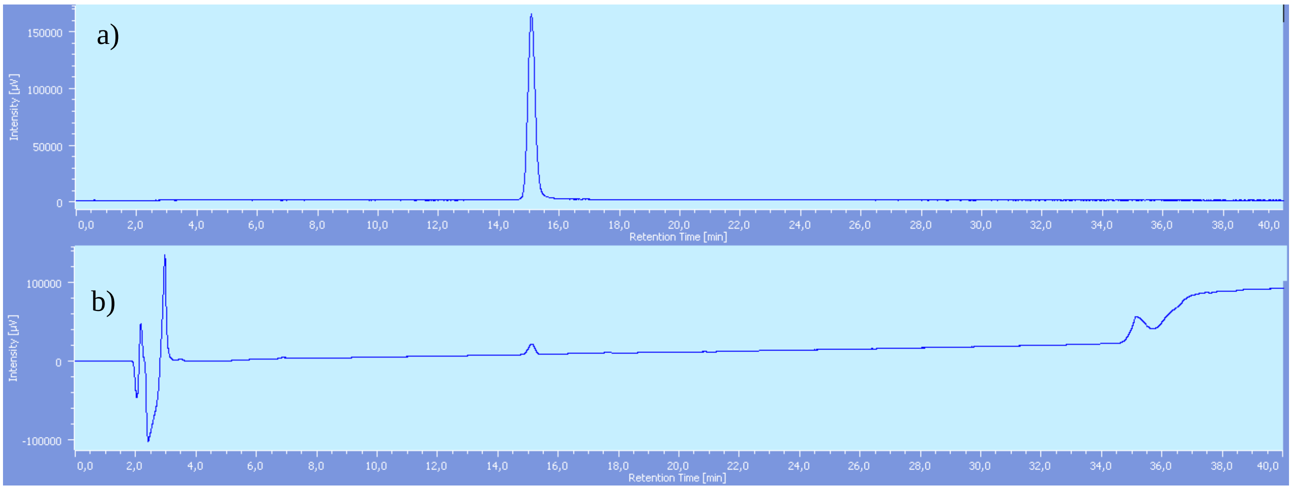

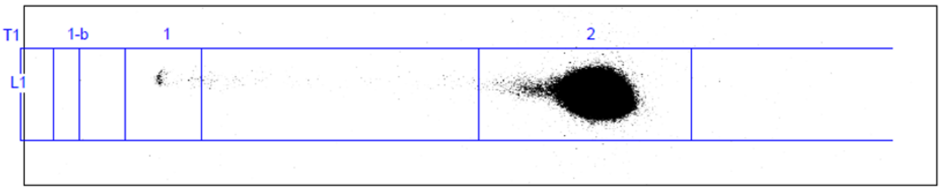

Lane #1
Background Subtraction: Regions = 261.422 DLU /mm2

ID	Dist From Origin(mm)	RF	Gross DLU	Background Subtract	Net DLU	Net % Sum	Net % Max Reg.
1 - Prof	17.1	0.0	78,137.4	22,620.8	55,516.6	0.3	0.3
2 - Prof	66.4	0.7	20,299,526.0	63,664.7	20,235,861.3	99.7	100.0
1-b - Prof			7,695.7				
Lane			20,771,526.0	261,421.8	20,510,104.2		
UnRes			393,862.6	175,136.3	218,726.3		

Figure S41: *HPLC chromatogram and Radio-TLC of* ***[^{18}F]1*** *at t=4h in solution*. HPLC: a) Radiochemical detector; b) UV detector. Analytical RP-HPLC condition: column XTerra C18 5µm, 250x4.6 mm; water +0.1%TFA/acetonitrile +0.1%TFA isocratic at 80:20 for 1 min, gradient from 80:20 to 60:40 in 30 min, gradient from 60:40 to 20:80 in 2 min; 1 ml/min, 220 nm, UV detector. R_t **[^{18}F]1**: 14.9 min. Injected activity: ~4.5 MBq. TLC: Eluent ammonium acetate 0.05M, pH 5.5 / ACN 1:1. Injected activity: 7.5 kBq.

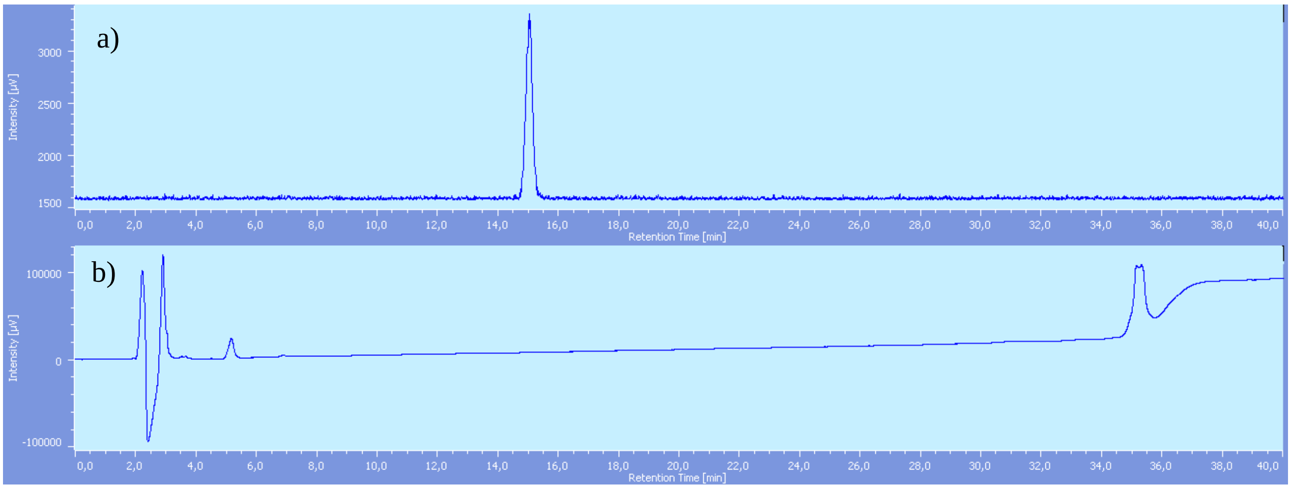

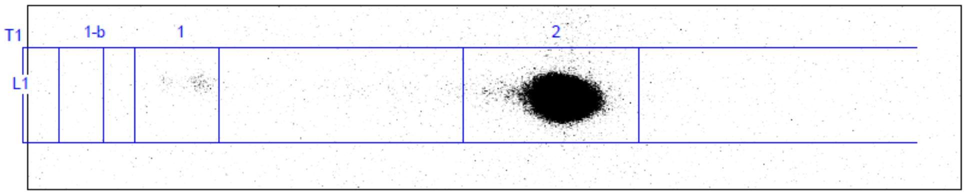

c)

Lane #1
Background Subtraction: Baseline

ID	Dist From Origin(mm)	RF	Gross DLU	Background Subtract	Net DLU	Net % Sum	Net % Max Reg.
**1 - Prof**	17.9	0.0	51,182.0	26,868.1	24,313.9	0.3	0.3
**2 - Prof**	60.7	0.6	8,062,473.3	56,017.5	8,006,455.9	99.7	100.0
**1-b - Prof**			14,447.9				
**Lane**			8,383,451.7	284,142.9	8,099,308.8		
**UnRes**			269,796.3	201,257.3	68,539.0		

Figure S42: *HPLC chromatogram and Radio-TLC of **[^{18}F]1** at t=2h incubation in plasma*. HPLC: a) Radiochemical detector; b) UV detector. Analytical RP-HPLC condition: column XTerra C18 5µm, 250x4.6 mm; water +0.1%TFA/acetonitrile +0.1%TFA isocratic at 80:20 for 1 min, gradient from 80:20 to 60:40 in 30 min, gradient from 60:40 to 20:80 in 2 min; 1 ml/min, 220 nm, UV detector. R$_t$ **[^{18}F]1**: 14.9 min. Injected activity: ~200 kBq. TLC: c) Report of **[^{18}F]1** after plasma pellet precipitation by ACN: Deposition of ACN supernatant. Eluent ammonium acetate 0.05M, pH 5.5 / ACN 1:1. Injected activity: 7.5 kBq.

a)

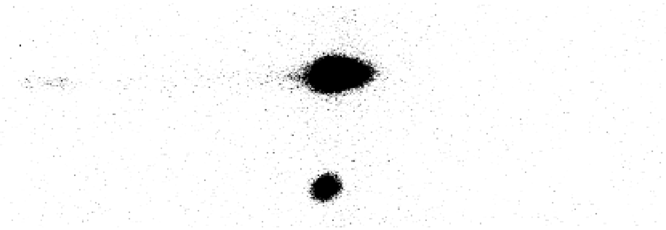

b)

c)

Total activity incubated	3.6 MBq
Supernatant activity	3.2 MBq
Pellet activity	0.4 MBq

Figure S43: *Radio-TLC sheet of **[^{18}F]1** at t=2h incubation in plasma*. TLC: a) **[^{18}F]1** after plasma pellet precipitation by ACN. Deposition of ACN supernatant; b) Deposition of plasma containing **[^{18}F]1** before pellet precipitation by ACN. C) table of recovery activity. Eluent ammonium acetate 0.05M, pH 5.5 / ACN 1:1. Injected activity: 7.5 kBq.

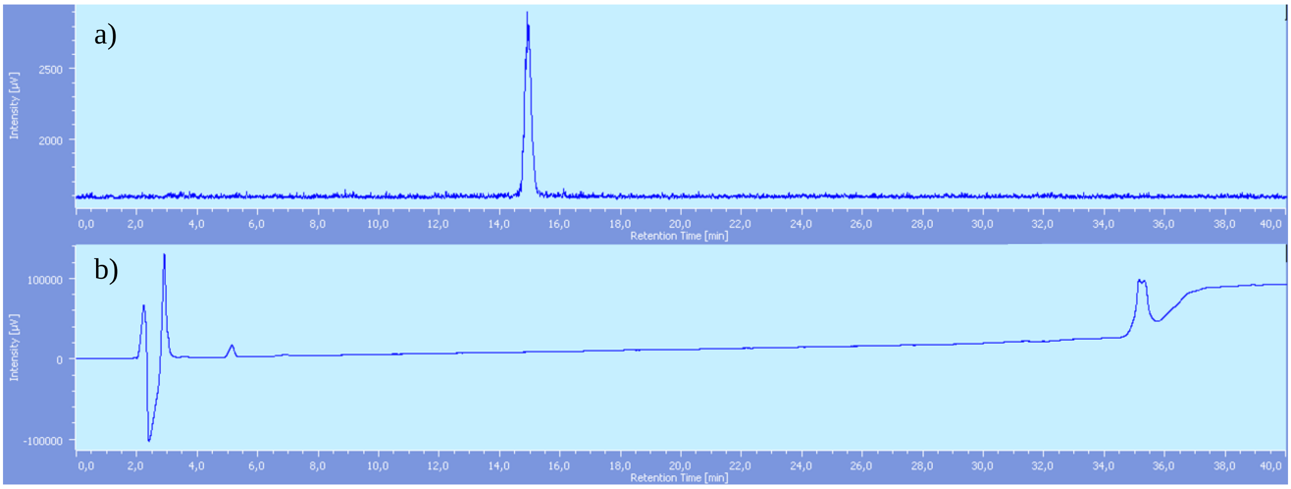

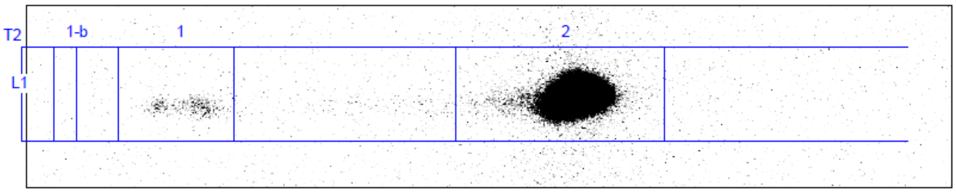

c)

Lane #1
Background Subtraction: Regions = 244.358 DLU /mm2

ID	Dist From Origin(mm)	RF	Gross DLU	Background Subtract	Net DLU	Net % Sum	Net % Max Reg.
1 - Prof	18.0	0.0	75,992.4	31,825.4	44,167.0	0.4	0.4
2 - Prof	62.8	0.6	10,033,493.3	57,547.3	9,975,946.0	99.6	100.0
1-b - Prof			5,885.5				
Lane			10,303,034.4	244,358.1	10,058,676.3		
UnRes			193,548.6	154,985.4	38,563.3		

Figure S44: *HPLC chromatogram and Radio-TLC of **[^{18}F]1** at t=4h incubation in plasma*. HPLC: a) Radiochemical detector; b) UV detector. Analytical RP-HPLC condition: column XTerra C18 5µm, 250x4.6 mm; water +0.1%TFA/acetonitrile +0.1%TFA isocratic at 80:20 for 1 min, gradient from 80:20 to 60:40 in 30 min, gradient from 60:40 to 20:80 in 2 min; 1 ml/min, 220 nm, UV

detector. R_t **[^{18}F]1**: 14.9 min. Injected activity: ~170 kBq. TLC: c) Report of **[^{18}F]1** after plasma pellet precipitation by ACN. Deposition of ACN supernatant. Eluent ammonium acetate 0.05M, pH 5.5 / ACN 1:1. Injected activity: 7.5 kBq.

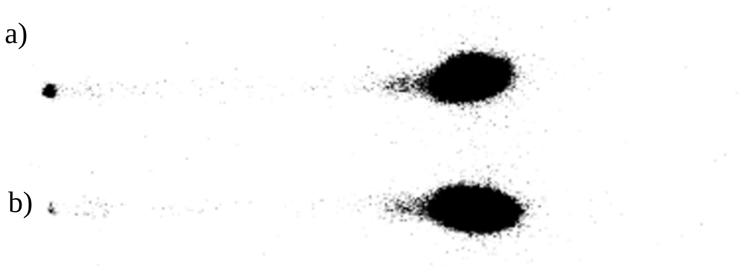

c)

Total activity incubated	3.89 MBq
Supernatant activity	3.44 MBq
Pellet activity	0.45 MBq

Figure S45: *Radio-TLC sheet of **[^{18}F]1** at t=4h incubation in plasma*. TLC: a) **[^{18}F]1** after plasma pellet precipitation by ACN. Deposition of ACN supernatant; b) Deposition of plasma containing **[^{18}F]1** before pellet precipitation by ACN. C) table of recovery activity. Eluents ammonium acetate 0.05M, pH 5.5 / ACN 1:1. Injected activity: 7.5 kBq.

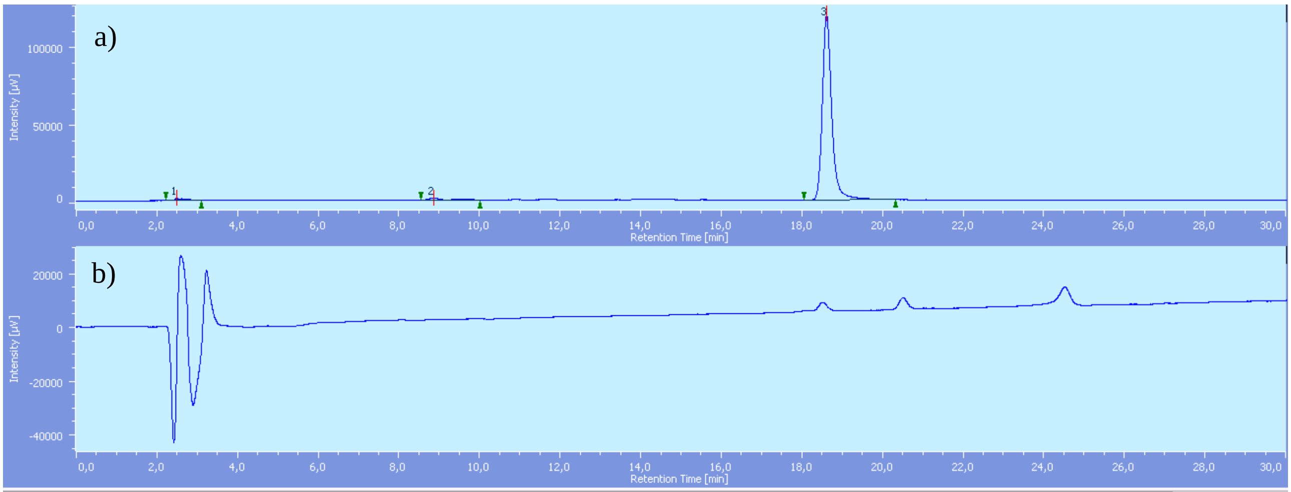

Peak Info. Table: 18FPSMA617RESCA t0 24mag22

#	Peak Name	CH	tR	Area	Height	Area%	Height%	Quantity	NTP	Resolution	Symmetry Factor	Warning
1	Unknown	1	2,483	24815	1384	1,244	1,139	N/A	392	14,352	4,340	
2	Unknown	1	8,867	35971	1481	1,803	1,218	N/A	8255	24,860	2,900	
3	Unknown	1	18,603	1934585	118699	96,954	97,643	N/A	35483	N/A	1,244	

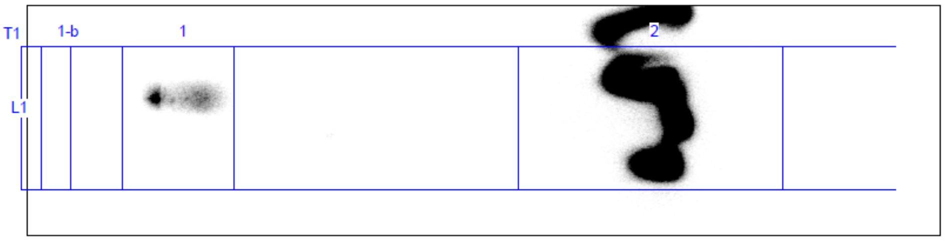

Lane #1
Background Subtraction: Baseline

ID	Dist From Origin(mm)	RF	Gross DLU	Background Subtract	Net DLU	Net % Sum	Net % Max Reg.
1 - Prof	18.1	0.0	2,276,595.7	104,408.3	2,172,187.4	2.3	2.3
2 - Prof	72.5	0.8	93,644,463.3	251,764.7	93,392,698.5	97.7	100.0
1-b - Prof			28,878.9				
Lane			97,068,247.2	830,083.2	96,238,164.0		
UnRes			1,147,188.2	473,910.1	673,278.1		

Figure S46: *HPLC chromatogram and Radio-TLC of* ***[^{18}F]2*** *at t=0 in solution*. HPLC: a) Radiochemical detector; b) UV detector. Analytical RP-HPLC condition: column XTerra C18 5µm, 250x4.6 mm; water +0.1%TFA/acetonitrile +0.1%TFA isocratic at 80:20 for 1 min, gradient from 80:20 to 60:40 in 30 min, gradient from 60:40 to 20:80 in 2 min; 1 ml/min, 220 nm, UV detector. Rt [^{18}F] AlF^{2+}: 2.5 min, Rt product **[^{18}F] 2**: 18.6 min, Rt PSMA-617-RESCA complexed with $[Al(OH)]^{2+}$: 20.7 min, Rt precursor **9**: 24.7 min. Injected activity: ~13 MBq. TLC: Eluent ammonium acetate 0.05M, pH 5.5 / ACN 1:1. Injected activity: 7.5 kBq.

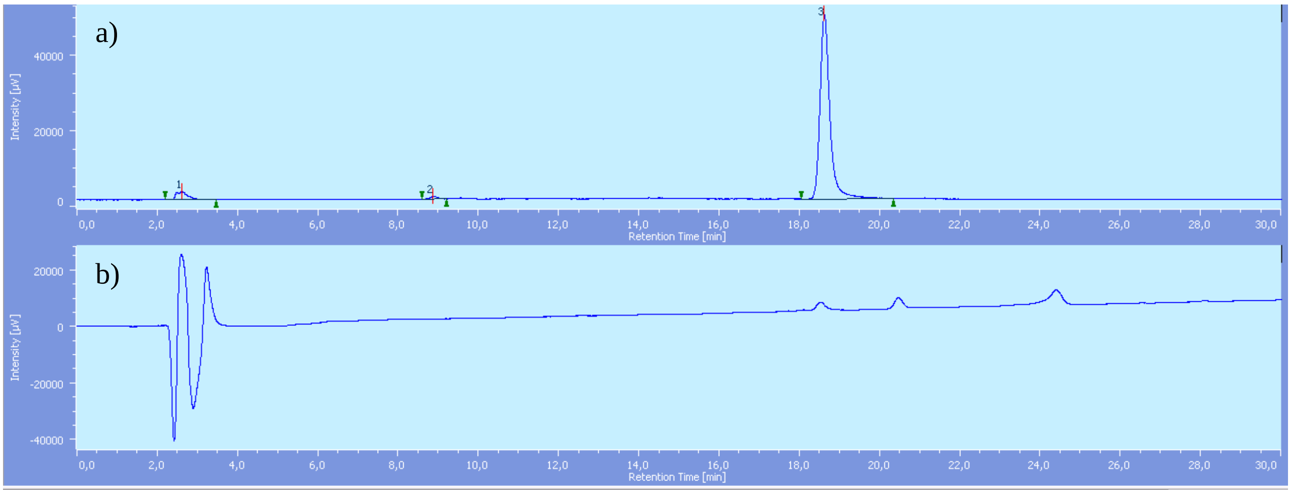

Peak Info. Table: 18FPSMA617RESCA t2h 24mag22

#	Peak Name	CH	tR	Area	Height	Area%	Height%	Quantity	NTP	Resolution	Symmetry Factor	Warning
1	Unknown	1	2,620	41453	2192	4,696	4,248	N/A	411	14,404	1,410	
2	Unknown	1	8,857	8366	654	0,948	1,268	N/A	10166	25,556	1,119	
3	Unknown	1	18,610	832903	48759	94,356	94,484	N/A	32338	N/A	1,355	

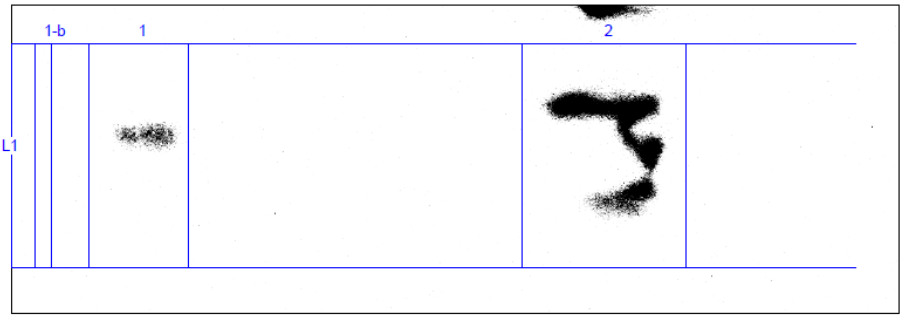

Lane #1
Background Subtraction: Baseline

ID	Dist From Origin(mm)	RF	Gross DLU	Background Subtract	Net DLU	Net % Sum	Net % Max Reg.
1 - Prof	16.5	0.0	692,427.9	29,006.5	663,421.4	4.9	5.2
2 - Prof	71.6	0.8	12,897,411.3	88,101.8	12,809,309.4	95.1	100.0
1-b - Prof			10,823.3				
Lane			13,810,976.9	242,658.8	13,568,318.0		
UnRes			221,137.7	125,550.5	95,587.2		

Figure S47: *HPLC chromatogram and Radio-TLC of **[^{18}F]2** at t=2h in solution*. HPLC: a) Radiochemical detector; b) UV detector. Analytical RP-HPLC condition: column XTerra C18 5µm, 250x4.6 mm; water +0.1%TFA/acetonitrile +0.1%TFA isocratic at 80:20 for 1 min, gradient from 80:20 to 60:40 in 30 min, gradient from 60:40 to 20:80 in 2 min; 1 ml/min, 220 nm, UV detector. Rt [^{18}F] AlF^{2+}: 2.5 min, Rt product **[^{18}F] 2**: 18.6 min, Rt PSMA-617-RESCA complexed with $[Al(OH)]^{2+}$: 20.7 min, Rt precursor **9**: 24.7 min. Injected activity: ~6.5 MBq. TLC: Eluent ammonium acetate 0.05M, pH 5.5 / ACN 1:1. Injected activity: 7.5 kBq.

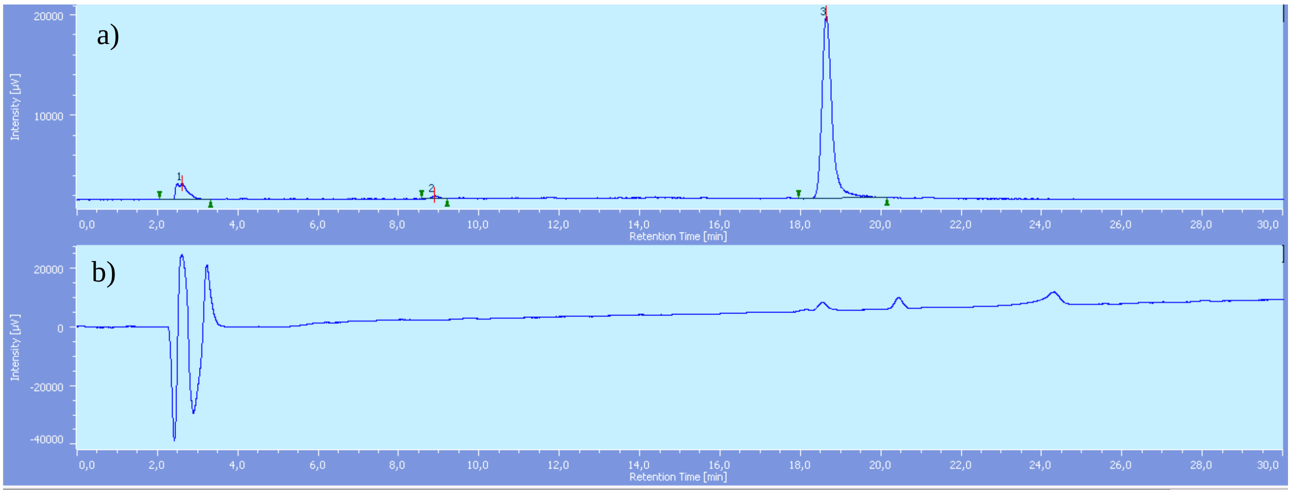

Peak Info. Table: 18FPSMA617RESCA t4h 24mag22

#	Peak Name	CH	tR	Area	Height	Area%	Height%	Quantity	NTP	Resolution	Symmetry Factor	Warning
1	Unknown	1	2,607	31247	1598	8,914	7,958	N/A	386	15,157	1,500	
2	Unknown	1	8,890	3212	275	0,916	1,369	N/A	13967	27,136	1,217	
3	Unknown	1	18,623	316094	18209	90,170	90,672	N/A	31700	N/A	1,435	

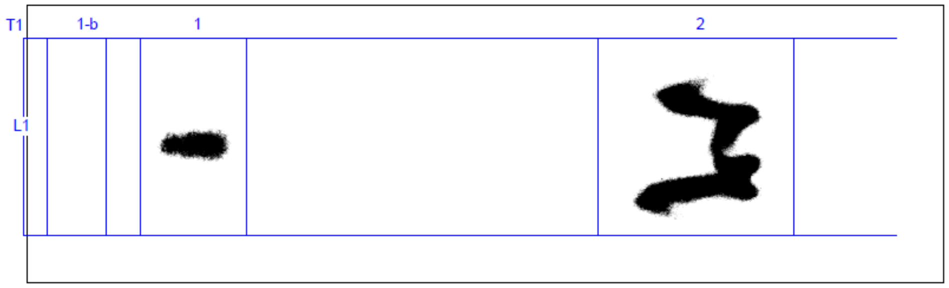

Lane #1
Background Subtraction: Baseline

ID	Dist From Origin(mm)	RF	Gross DLU	Background Subtract	Net DLU	Net % Sum	Net % Max Reg.
**1 - Prof**	18.8	0.1	2,318,118.5	81,217.6	2,236,900.9	10.6	11.8
**2 - Prof**	76.4	0.8	19,053,394.4	151,004.6	18,902,389.8	89.4	100.0
**1-b - Prof**			45,722.5				
**Lane**			21,923,836.4	674,407.1	21,249,429.3		
**UnRes**			552,323.5	442,184.8	110,138.6		

Figure S48: *HPLC chromatogram and Radio-TLC of* ***[^{18}F]2*** *at t=4h in solution*. HPLC: a) Radiochemical detector; b) UV detector. Analytical RP-HPLC condition: column XTerra C18 5µm, 250x4.6 mm; water +0.1%TFA/acetonitrile +0.1%TFA isocratic at 80:20 for 1 min, gradient from 80:20 to 60:40 in 30 min, gradient from 60:40 to 20:80 in 2 min; 1 ml/min, 220 nm, UV detector. Rt [^{18}F] AlF^{2+}: 2.5 min, Rt product **[^{18}F] 2**: 18.6 min, Rt PSMA-617-RESCA complexed with $[Al(OH)]^{2+}$: 20.7 min, Rt precursor **9**: 24.7 min. Injected activity: ~3.75 MBq. TLC: Eluent ammonium acetate 0.05M, pH 5.5 / ACN 1:1. Injected activity: 7.5 kBq.

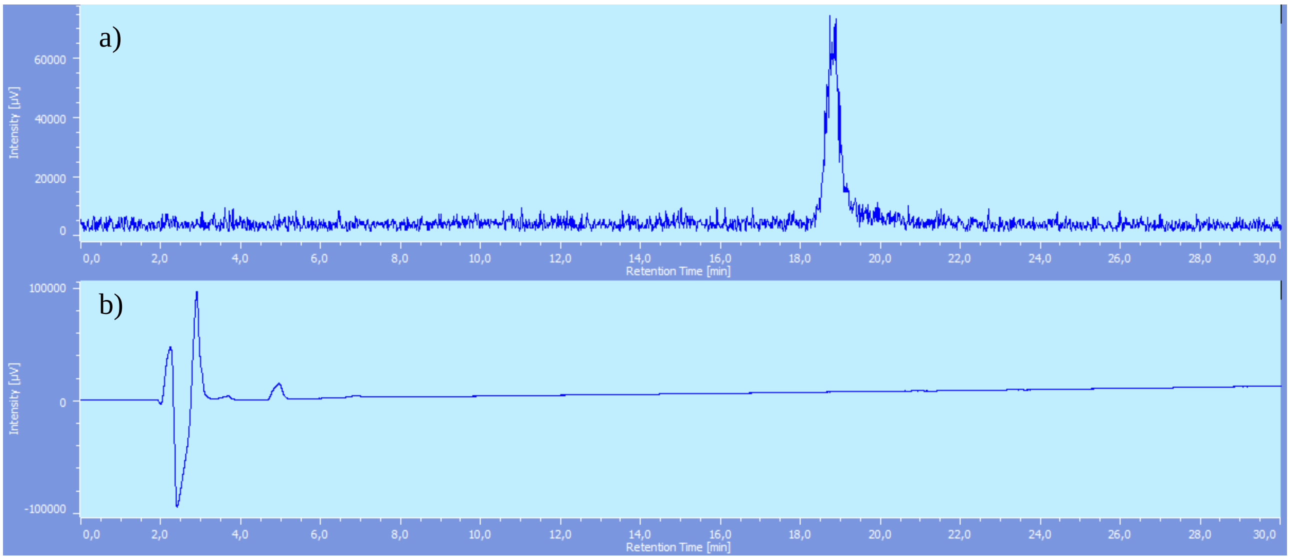

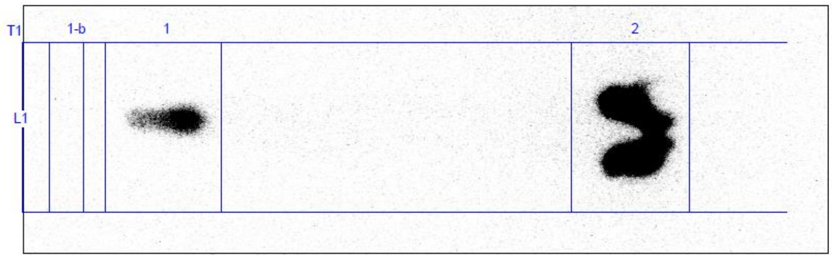

c)

Lane #1
Background Subtraction: Baseline

ID	Dist From Origin(mm)	RF	Gross DLU	Background Subtract	Net DLU	Net % Sum	Net % Max Reg.
1 - Prof	19.5	0.1	652,979.7	62,455.0	590,524.7	4.3	4.5
2 - Prof	79.8	0.9	13,290,051.7	63,924.5	13,226,127.2	95.7	100.0
1-b - Prof			18,001.7				
Lane			14,387,007.0	411,835.4	13,975,171.6		
UnRes			443,975.6	285,455.9	158,519.7		

Figure S49: *HPLC chromatogram and Radio-TLC of* ***[18F]2*** *at t=2h incubation in plasma*. HPLC: a) Radiochemical detector; b) UV detector. Analytical RP-HPLC condition: column XTerra C18 5µm, 250x4.6 mm; water +0.1%TFA/acetonitrile +0.1%TFA isocratic at 80:20 for 1 min, gradient from 80:20 to 60:40 in 30 min, gradient from 60:40 to 20:80 in 2 min; 1 ml/min, 220 nm, UV detector. Rt [^{18}F] AlF^{2+}: 2.5 min, Rt product **[^{18}F] 2**: 18.6 min. Injected activity: ~160 kBq. TLC: c) Report of **[^{18}F]2** after plasma pellet precipitation by ACN. Deposition of ACN supernatant. Eluent ammonium acetate 0.05M, pH 5.5 / ACN 1:1. Injected activity: 7.5 kBq.

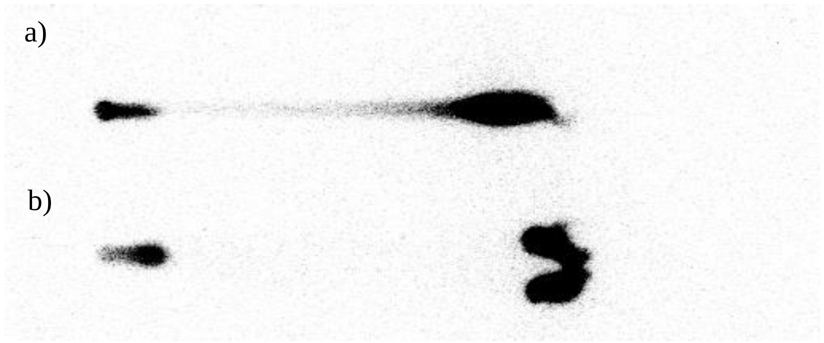

c)

Total activity incubated	3.91 MBq
Supernatant activity	3.46 MBq
Pellet activity	0.45 MBq

Figure S50: *Radio-TLC sheet of* ***[18F]2*** *at t=2h incubation in plasma*. TLC: a) Deposition of plasma containing **[^{18}F]2** before pellet precipitation by ACN; b) **[^{18}F]2** after plasma pellet precipitation by ACN. Deposition of ACN supernatant. c) table of recovery activity. Eluent ammonium acetate 0.05M, pH 5.5 / ACN 1:1. Injected activity: 7.5 kBq.

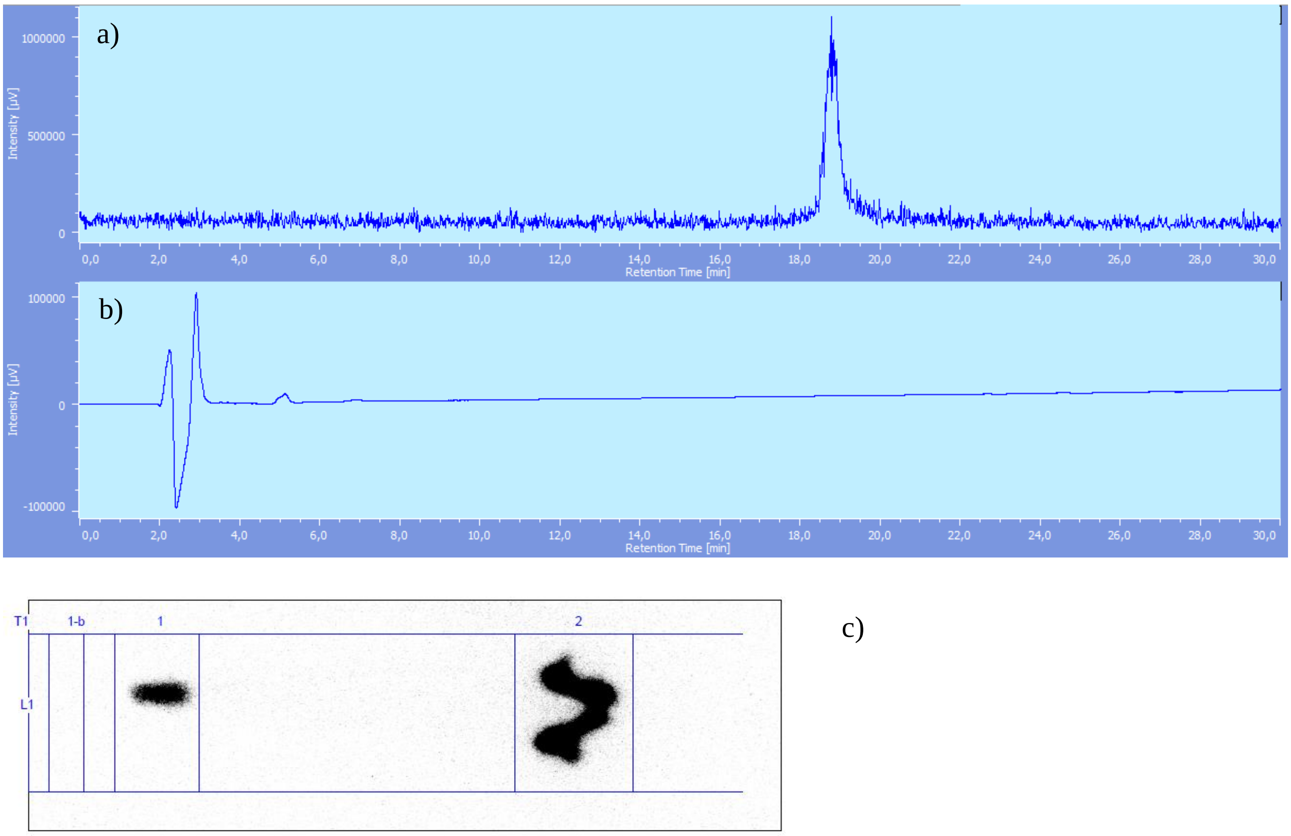

Lane #1
Background Subtraction: Baseline

ID	Dist From Origin(mm)	RF	Gross DLU	Background Subtract	Net DLU	Net % Sum	Net % Max Reg.
1 - Prof	18.1	0.0	847,778.1	48,763.2	799,015.0	6.8	7.3
2 - Prof	75.8	0.8	11,033,331.1	67,828.5	10,965,502.6	93.2	100.0
1-b - Prof			20,531.9				
Lane			12,335,019.4	411,003.8	11,924,015.6		
UnRes			453,910.2	294,412.2	159,498.0		

Figure S51: *HPLC chromatogram and Radio-TLC of **[^{18}F]2** at t=4h incubation in plasma*. HPLC: a) Radiochemical detector; b) UV detector. Analytical RP-HPLC condition: column XTerra C18 5µm, 250x4.6 mm; water +0.1%TFA/acetonitrile +0.1%TFA isocratic at 80:20 for 1 min, gradient from 80:20 to 60:40 in 30 min, gradient from 60:40 to 20:80 in 2 min; 1 ml/min, 220 nm, UV detector. Rt [^{18}F] AlF^{2+}: 2.5 min, Rt product **[^{18}F] 2**: 18.6 min. Injected activity: ~ 170 kBq. TLC: c) Report of **[^{18}F]2** after plasma pellet precipitation by ACN. Deposition of ACN supernatant. Eluent ammonium acetate 0.05M, pH 5.5 / ACN 1:1. Injected activity: 7.5 kBq.

a)

b)

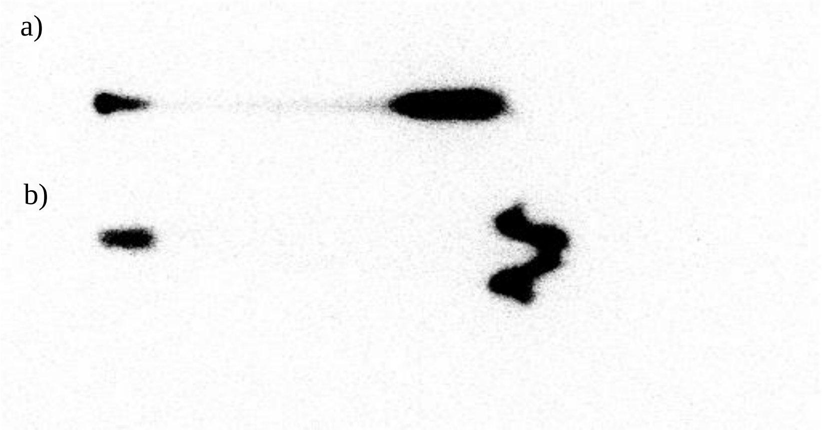

c)

Total activity incubated	4.62 MBq
Supernatant activity	4.05 MBq
Pellet activity	0.57 MBq

Figure S52: *Radio-TLC sheet of* ***[¹⁸F]2*** *at t=4h incubation in plasma*. TLC: a) Deposition of plasma containing **[^{18}F]2** before pellet precipitation by ACN; b) **[^{18}F]2** after plasma pellet precipitation by ACN. Deposition of ACN supernatant. c) table of recovery activity. Eluent ammonium acetate 0.05M, pH 5.5 / ACN 1:1. Injected activity: 7.5 kBq.

MS SPECTRA

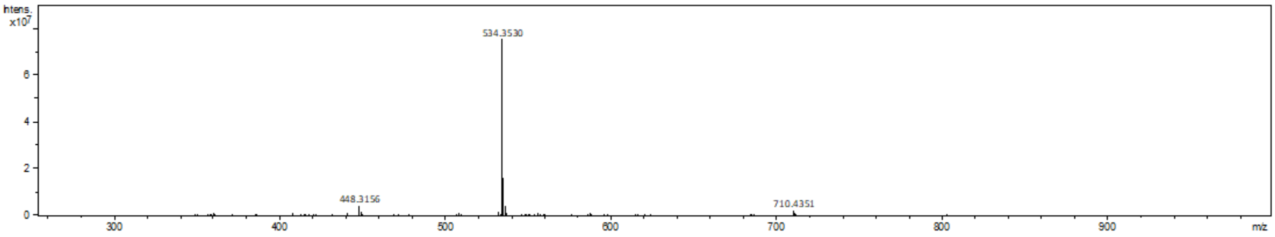

Figure S53: MS (m/z) (MS ESI+) of Compound **4**

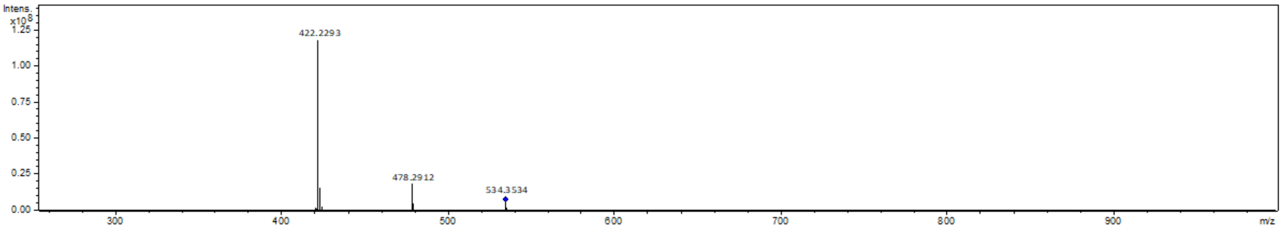

Figure S54: MS2 (m/z) (MS ESI+) of Compound **4**

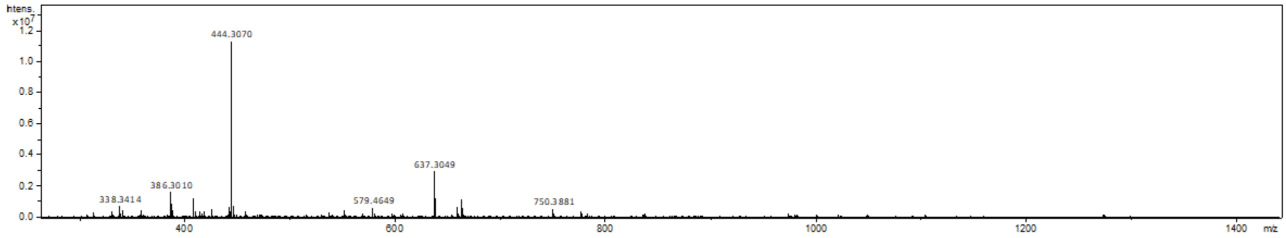

Figure S55: MS (m/z) (MS ESI+) of Compound **5**

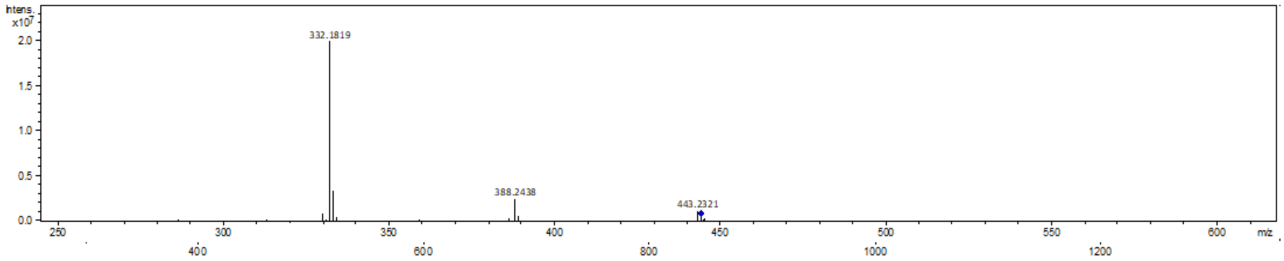

Figure S56: MS2 (m/z) (MS ESI+) of Compound **5**

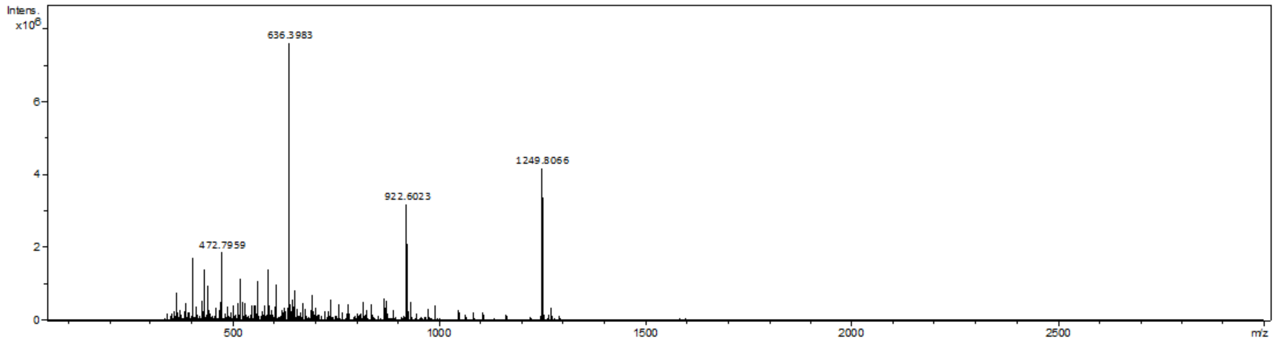

Figure S57: MS (m/z) (MS ESI+) of Compound **6**

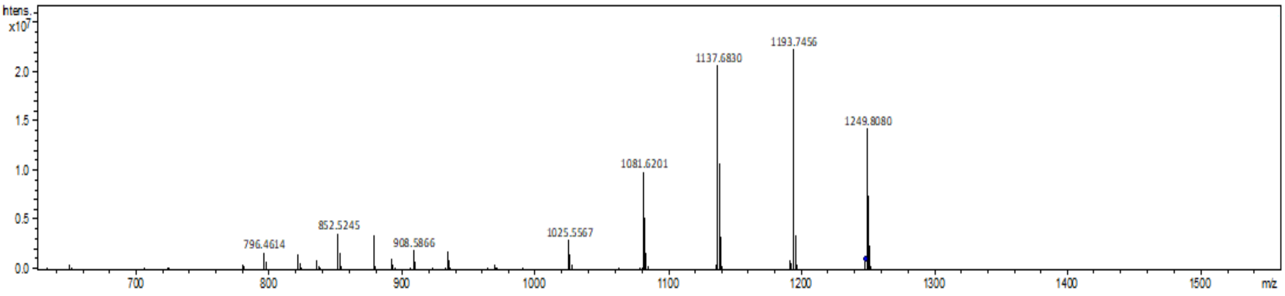

Figure S58: MS2 (m/z) (MS ESI+) of Compound **6**

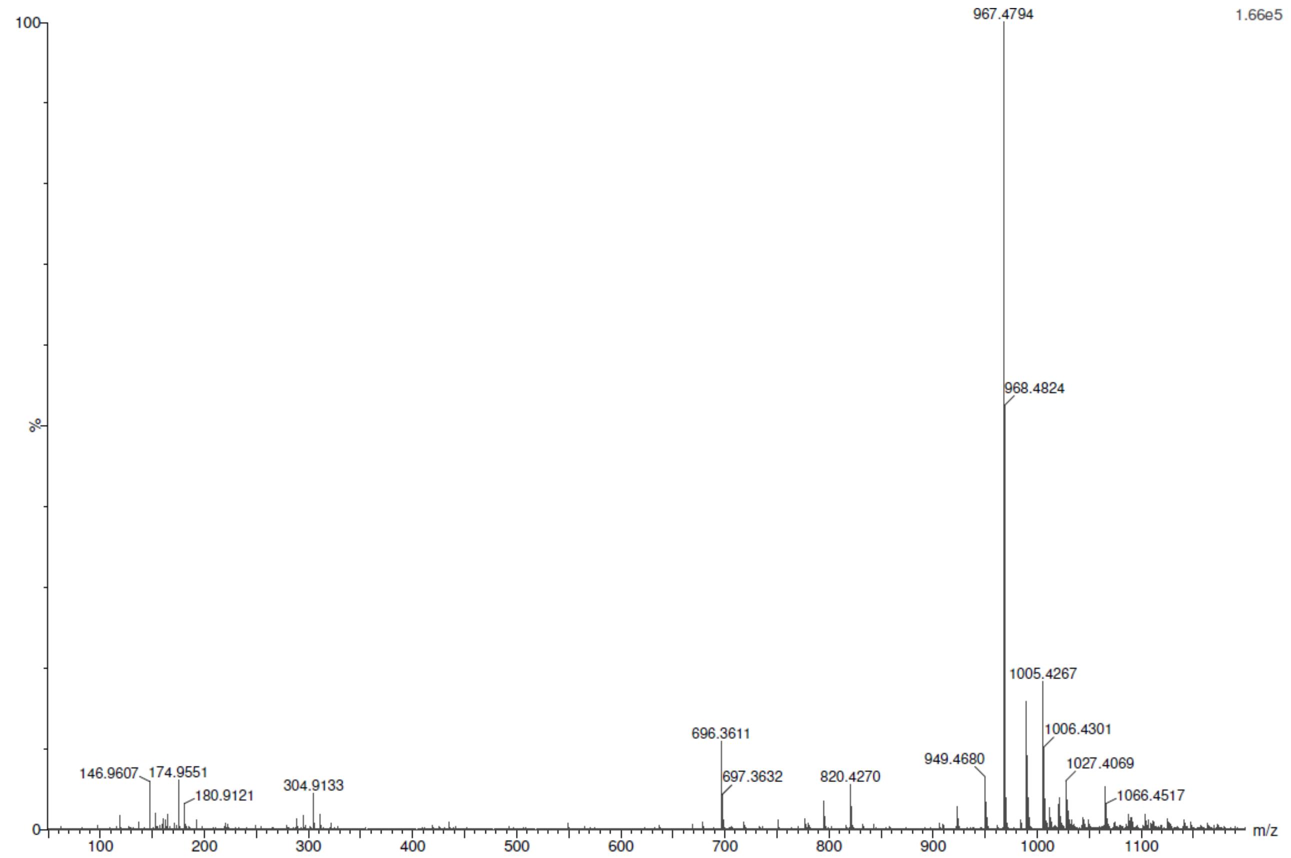

Figure S59: MS (m/z) (TOF MS ESI-) of Compound **7**

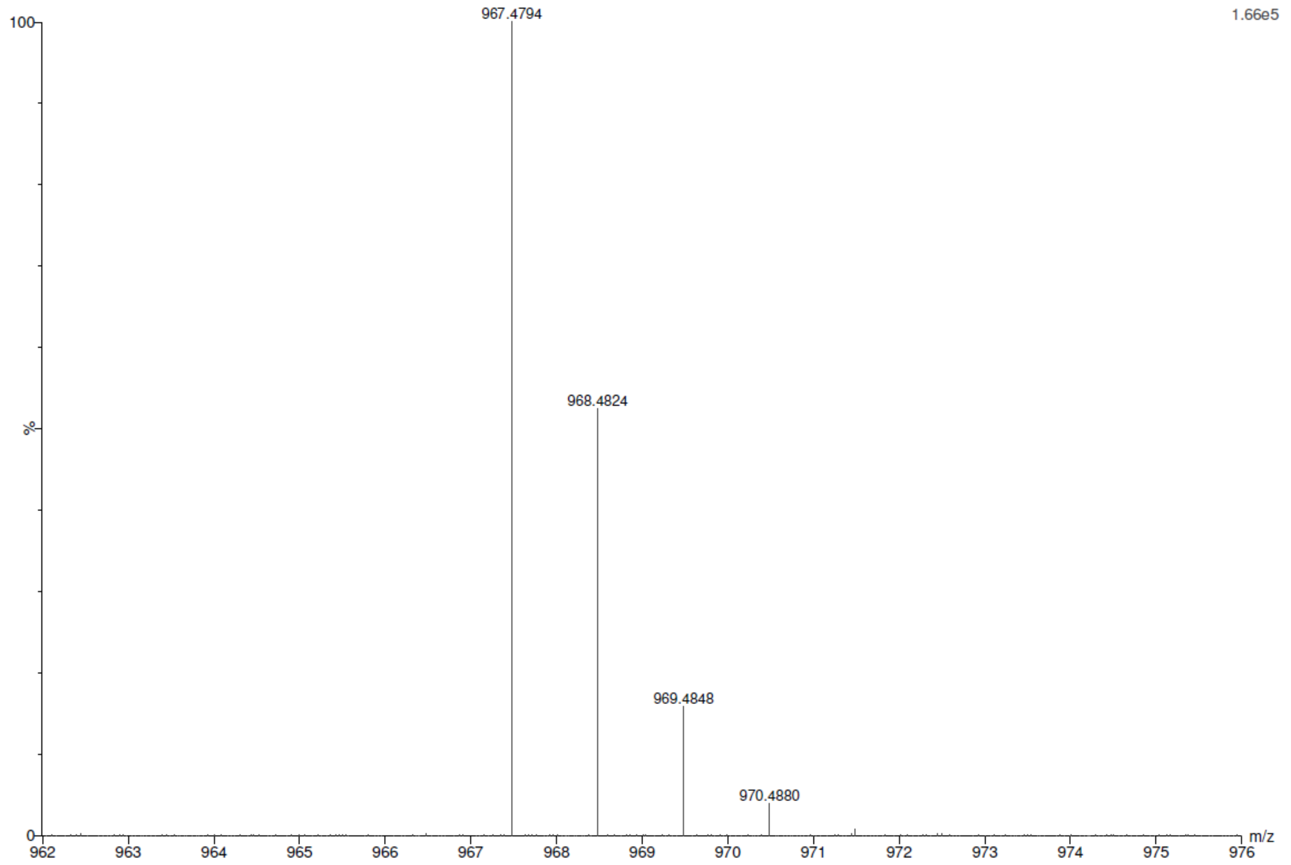

Figure S60: MS (m/z) (TOF MS ESI-) of Compound **7**

Elemental Composition Report | Page 1

Single Mass Analysis
Tolerance = 5.0 PPM / DBE: min = -2.5, max = 200.0
Element prediction: Off
Number of isotope peaks used for i-FIT = 5

Monoisotopic Mass, Even Electron Ions
1 formula(e) evaluated with 1 results within limits (all results (up to 1000) for each mass)
Elements Used:
C: 47-47 H: 67-69 N: 8-8 O: 14-14 Na: 0-3
MAIAN-214_MS+MSMS_06 315 (3.484) AM2 (Ar,40000.0,0.00,0.00); Cm (313:315) 1: TOF MS ES- 1.66e+005

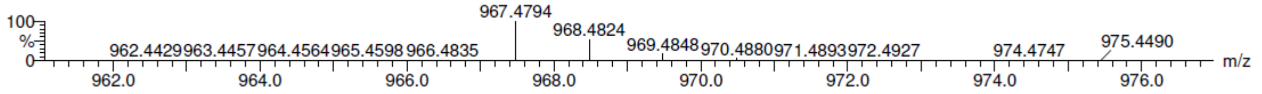

	Mass	Calc. Mass	mDa	PPM	DBE	i-FIT	Norm	Conf(%)	Formula
Minimum:					-2.5				
Maximum:			5.0	5.0	200.0				
	967.4794	967.4777	1.7	1.8	18.5	608.9	n/a	n/a	C47 H67 N8 O14

Figure S61: Elemental composition report (TOF MS ESI-) of Compound **7**

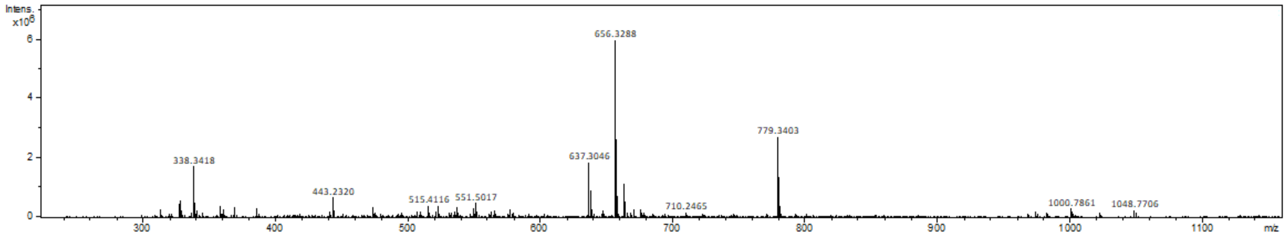

Figure S62: MS (m/z) (MS ESI+) of Compound **8**

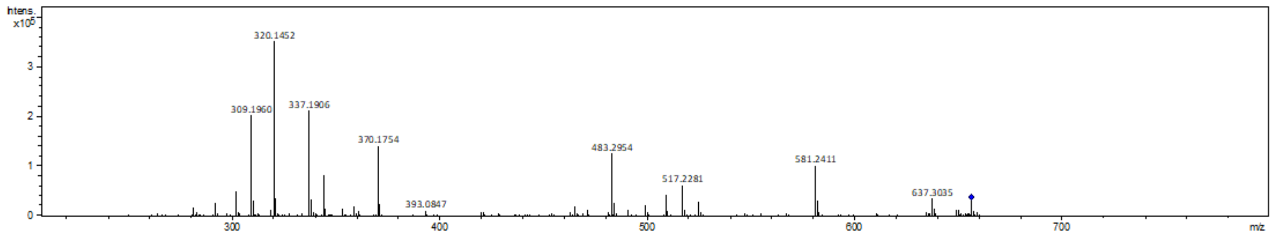

Figure S63: MS2 (m/z) (MS ESI+) of Compound **8**

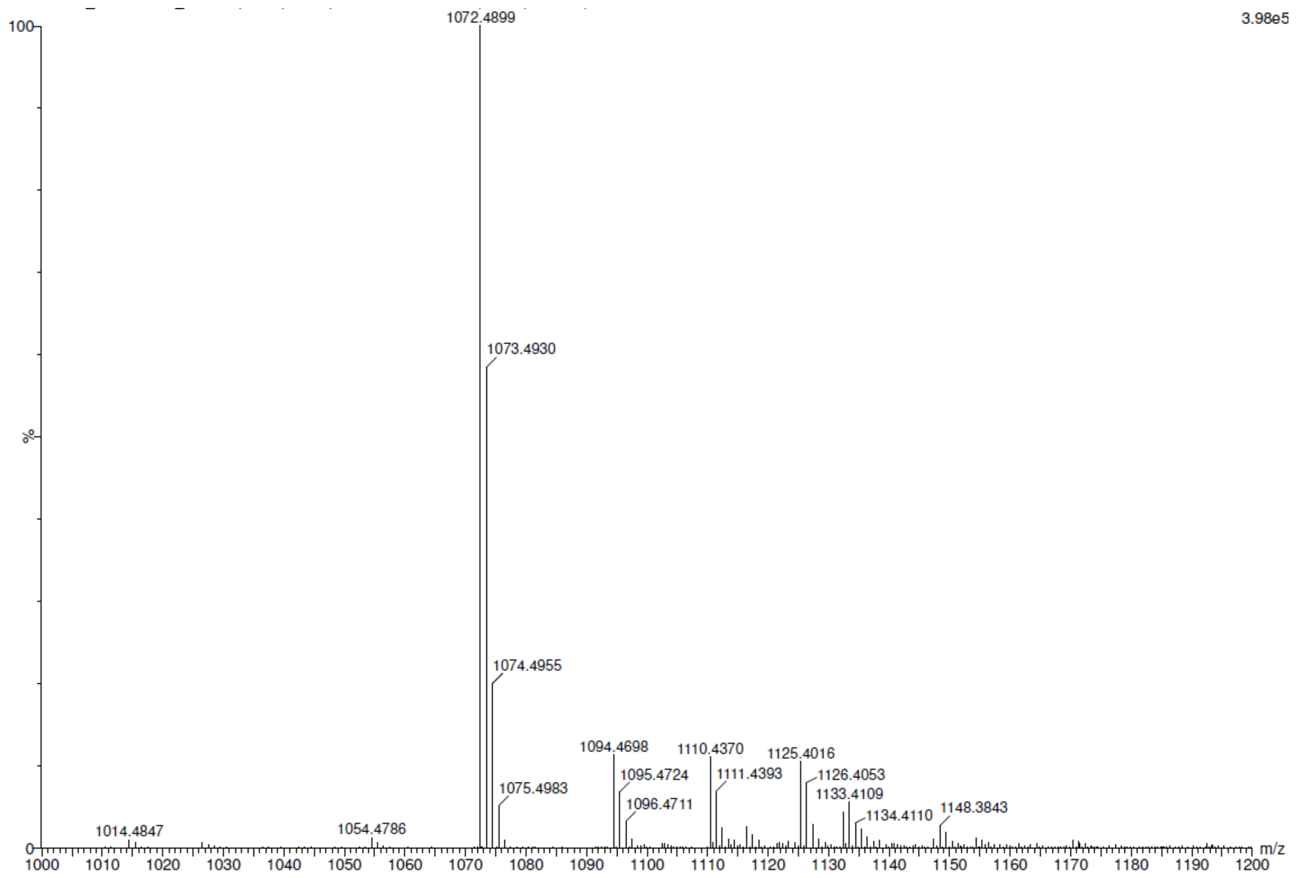

Figure S64: MS (m/z) (TOF MS ESI-) of Compound **9**

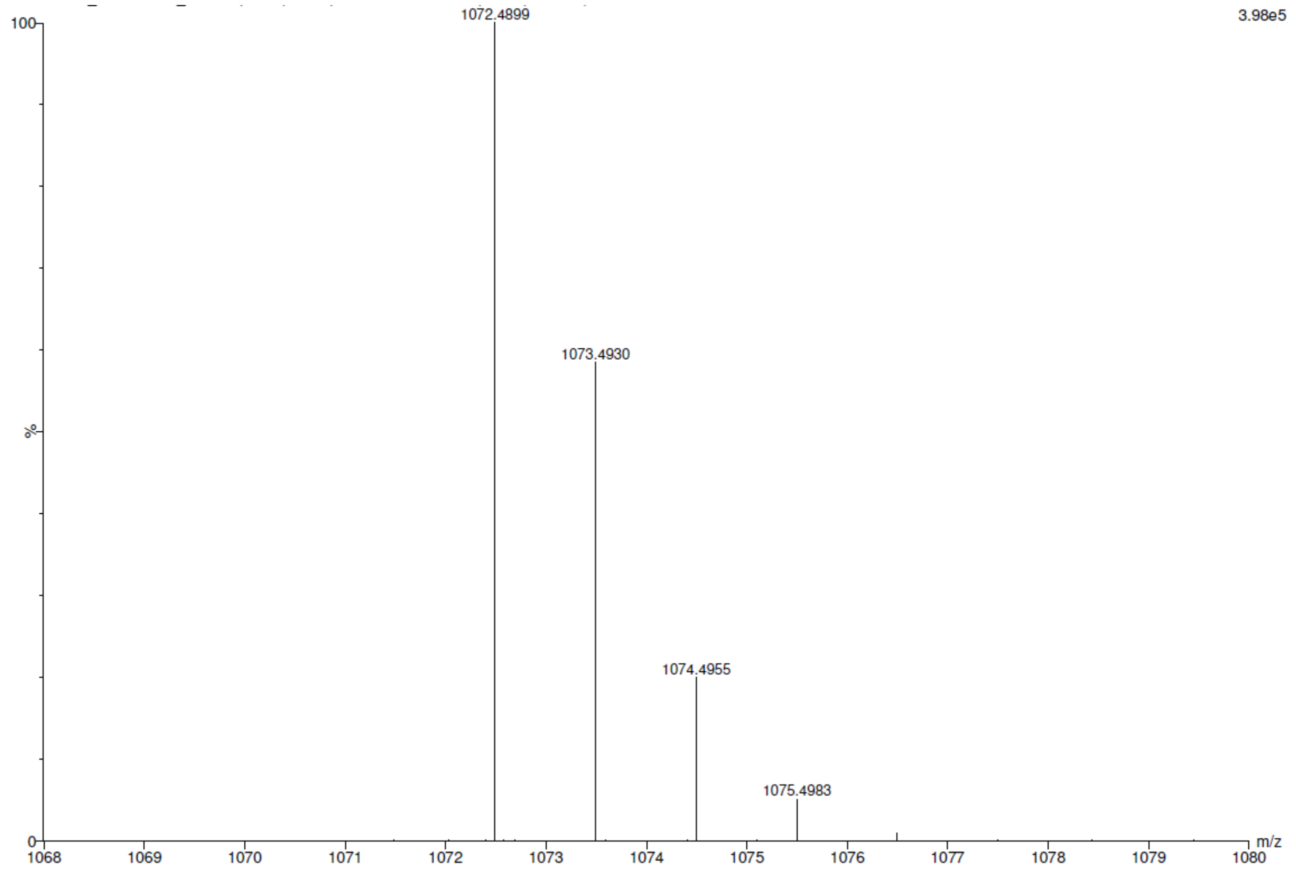

Figure S65: MS (m/z) (TOF MS ESI-) of Compound **9**

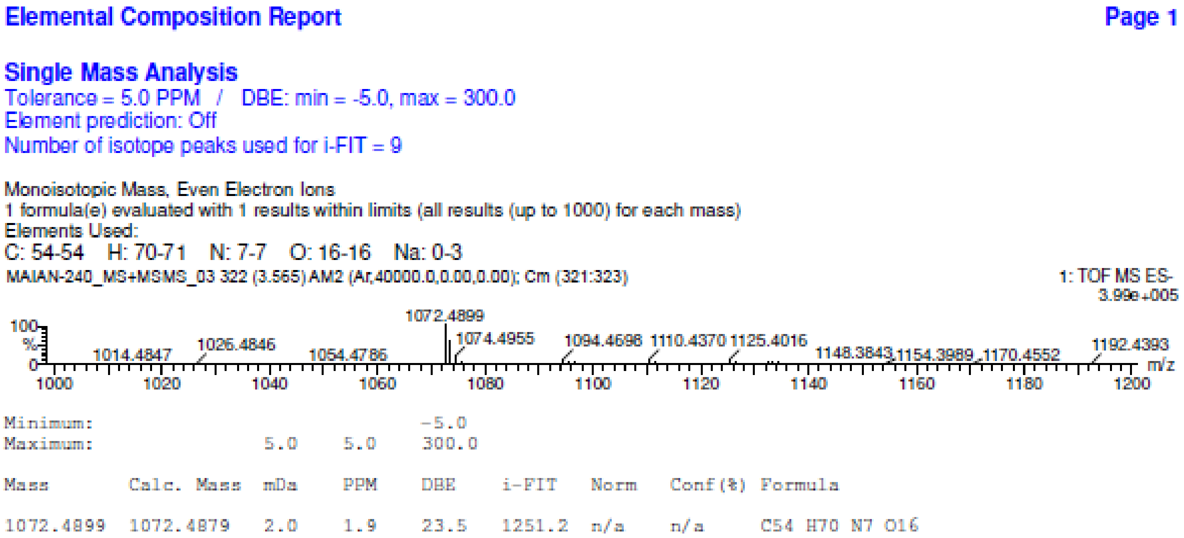

Elemental Composition Report Page 1

Single Mass Analysis
Tolerance = 5.0 PPM / DBE: min = -5.0, max = 300.0
Element prediction: Off
Number of isotope peaks used for i-FIT = 9

Monoisotopic Mass, Even Electron Ions
1 formula(e) evaluated with 1 results within limits (all results (up to 1000) for each mass)
Elements Used:
C: 54-54 H: 70-71 N: 7-7 O: 16-16 Na: 0-3

Minimum:				-5.0				
Maximum:		5.0	5.0	300.0				
Mass	Calc. Mass	mDa	PPM	DBE	i-FIT	Norm	Conf(%)	Formula
1072.4899	1072.4879	2.0	1.9	23.5	1251.2	n/a	n/a	C54 H70 N7 O16

Figure S66: Elemental composition report (TOF MS ESI-) of Compound **9**

BIODISTRIBUTION IMAGES

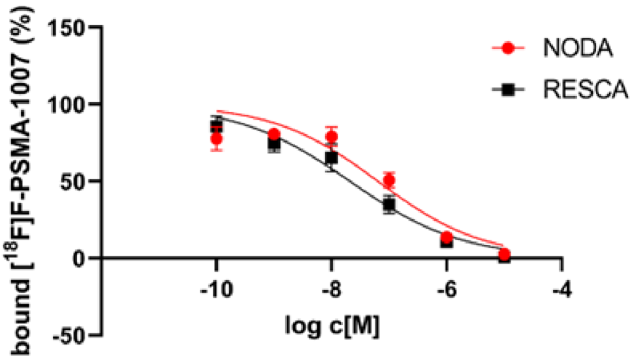

Figure S67: **In vitro binding assays in PC3-PIP cells**

[^{18}F]F-PSMA-1007 binding to PSMA in PC3-PIP cells in presence of different concentration of PSMA-617-NODA (**7**) and PSMA-617-RESCA (**9**). Inhibition constants were 64 and 20 nM for PSMA-617-NODA and PSMA-617-RESCA respectively.

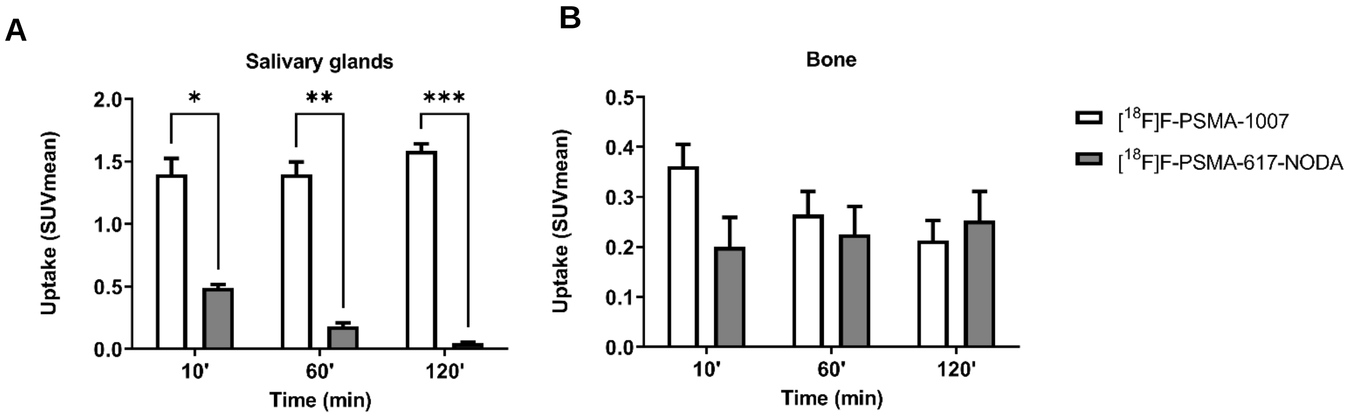

Figure S68. PET/CT uptake quantification of [^{18}F]F-PSMA-617-NODA ([^{18}F]1) in salivary glands and bone compared to clinical standard [^{18}F]F-PSMA-1007.

Radiotracer uptake was assessed after 10, 60 and 120 minutes post injection by whole-body PET/CT acquisitions. The quantification data are reported for A) salivary glands and B) bone as SUV mean. Bars, mean ± SD (n=4 mice); *, $p<0.05$, **, $p<0.01$ and ***$p<0.001$ by 2-way ANOVA Sidak's multiple comparison test.

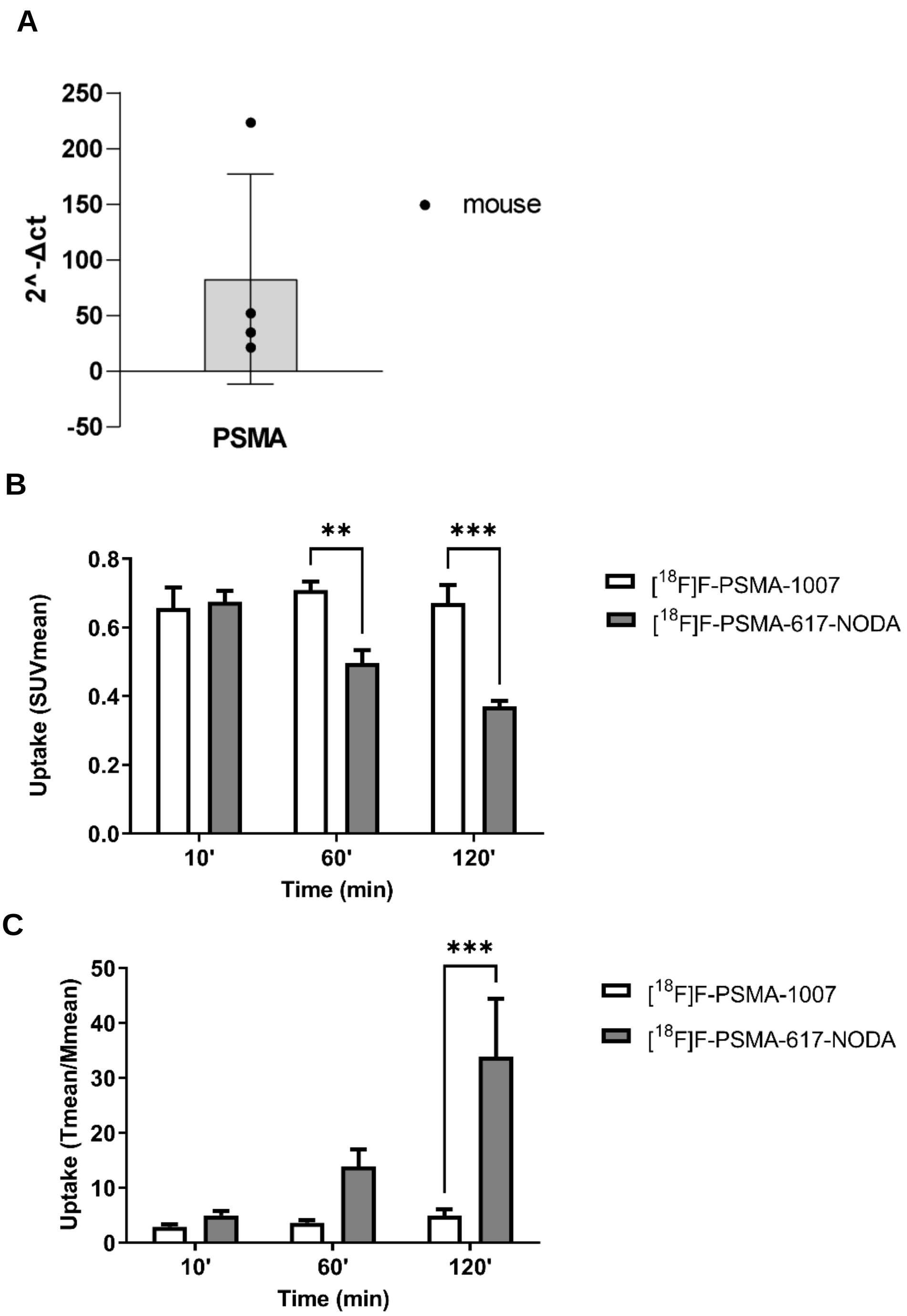

Figure S69: Uptake evaluation of clinical standard [^{18}F]F-PSMA-1007 in prostate cancer model (LNCaP cells) compared to [^{18}F]F-PSMA-617-NODA ([^{18}F]1).

A) The relative quantification data of PSMA expression in prostate tumor tissue are reported as Δct. **B)** The quantification data are reported as SUV mean of tumor uptake, and as **C)** tumor to muscle ratios (tumor SUV mean/muscle SUV mean). Bars, mean ± SD (n=4 mice); **, p<0.01 and ***p<0.001 by 2-way ANOVA Sidak's multiple comparison test.

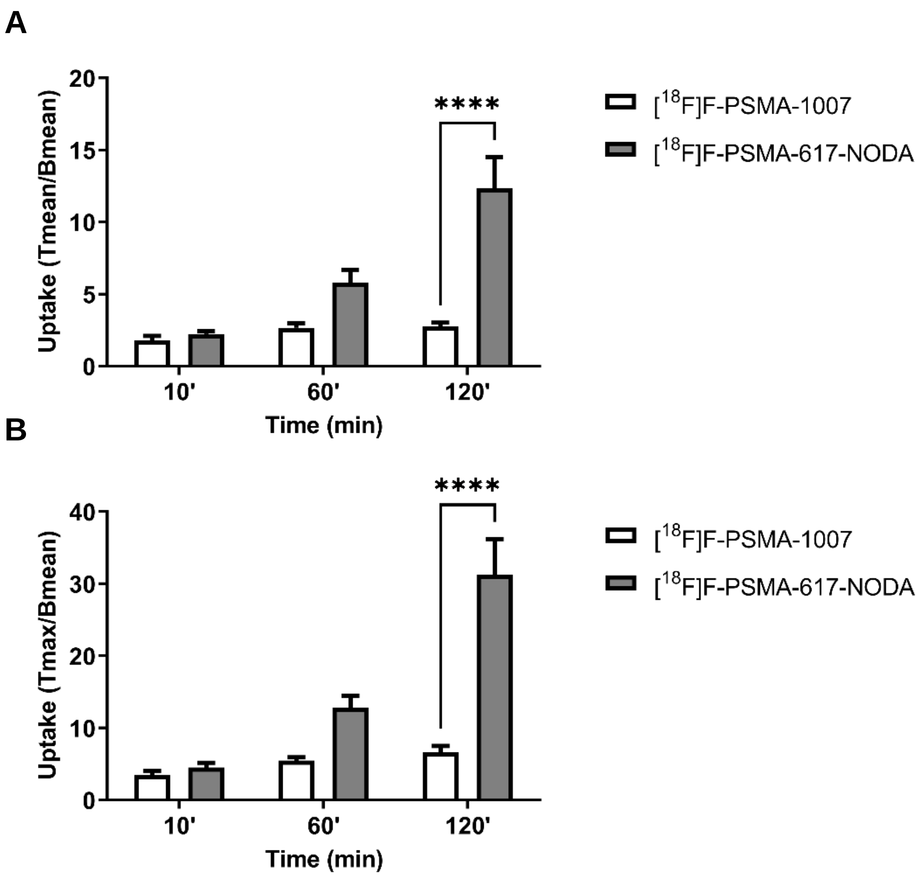

S70: Uptake evaluation of [^{18}F]F-PSMA-1007 and [^{18}F]F-PSMA-617-NODA ([^{18}F]1) in prostate cancer model

The quantification data are reported as tumour to blood ratios (**A)** tumour SUV mean/blood SUV mean, and **B)** tumour SUV max/blood SUV mean). Bars, mean ± SD (n=4 mice); ****p<0.0001 by 2-way ANOVA Sidak’s multiple comparison test.

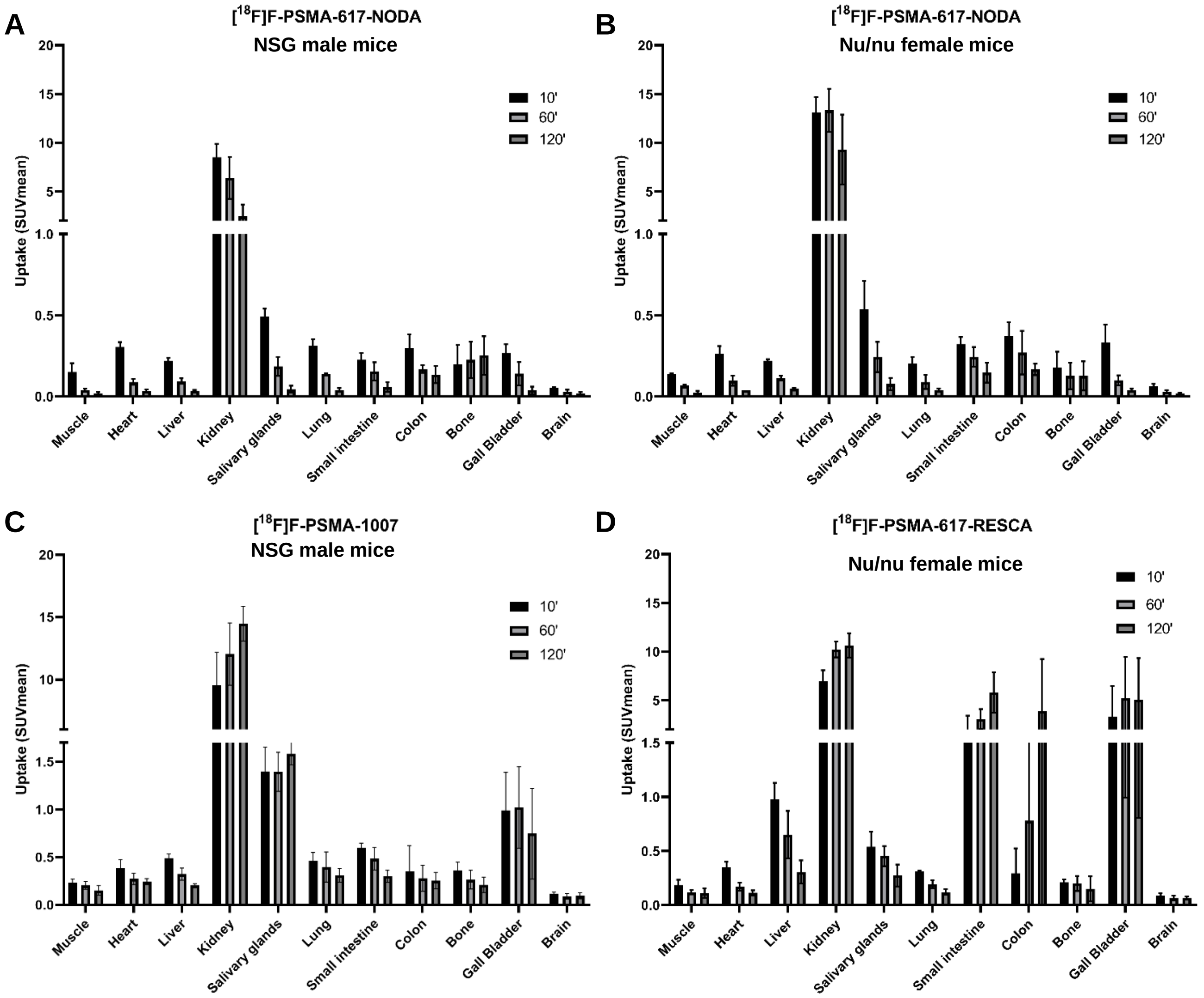

S71: Biodistribution of [^{18}F]F-PSMA-617-NODA ([^{18}F]1), [^{18}F]F-PSMA-1007 and [^{18}F]F-PSMA-617-RESCA ([^{18}F]2). Biodistribution of [^{18}F]F-PSMA-617-NODA in NSG male mice (A) and in nu/nu female mice (B), of [^{18}F]F-PSMA-1007 in NSG male mice (C) and of [^{18}F]F-PSMA-617-RESCA in nu/nu female mice (D).

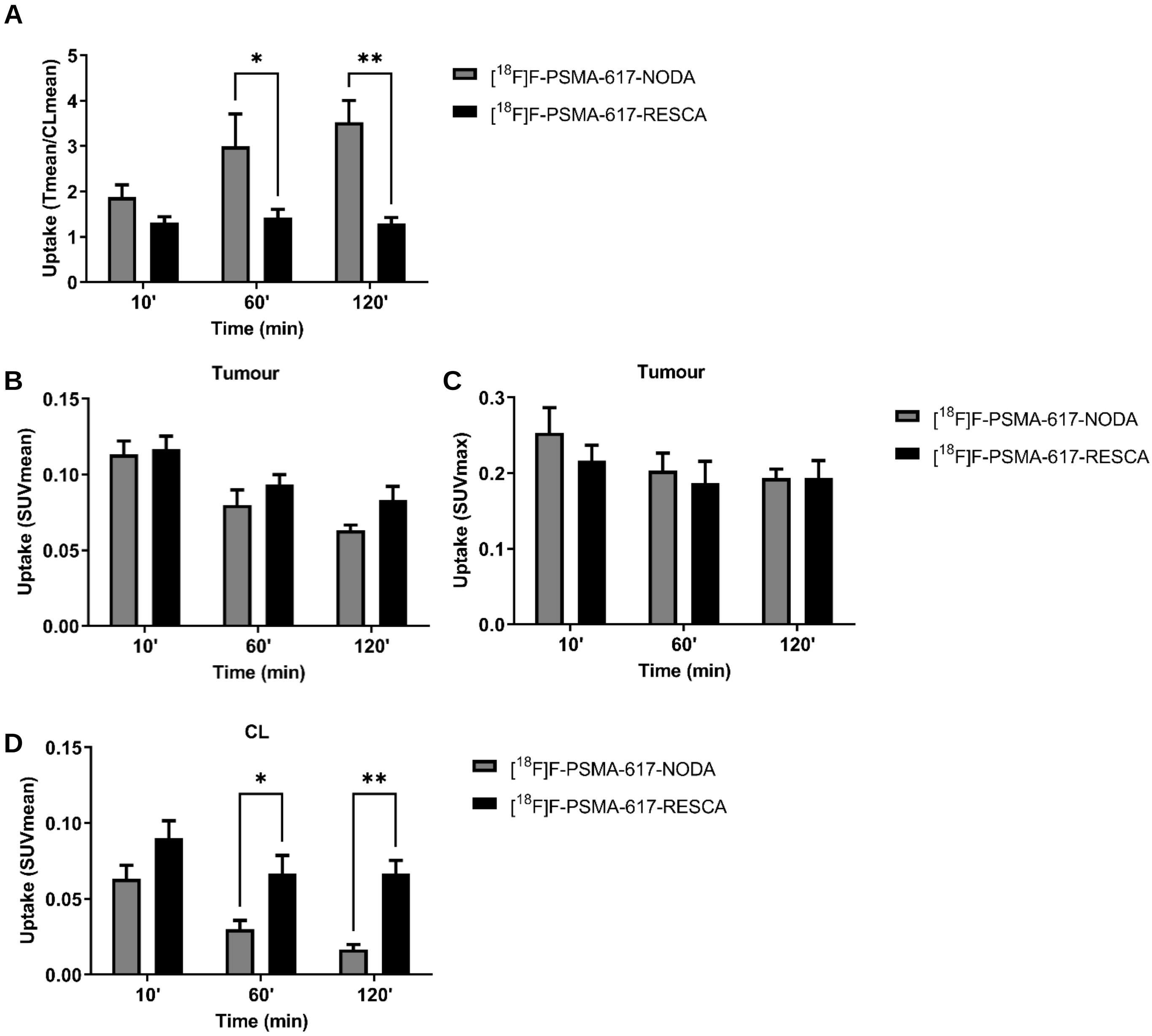

Figure S72. Uptake evaluation of [^{18}F]F-PSMA-617-NODA ([^{18}F]1) in GBM model (Gli36ΔEGFR cells) compared to [^{18}F]F-PSMA-617-RESCA ([^{18}F]2).

The tumour uptake results are expressed as tumor to contralateral ratios (tumour SUV mean/contralateral SUV mean) (A) and as absolute uptake expressed as SUVmean (B) and SUVmax (C). The graph reports absolute uptake in healthy brain contralateral to the tumour (CL) expressed as SUVmean (D). Bars, mean ± SD (n=3 mice); *, $p<0.05$, **, $p<0.01$ by 2-way ANOVA Sidak's multiple comparison test.

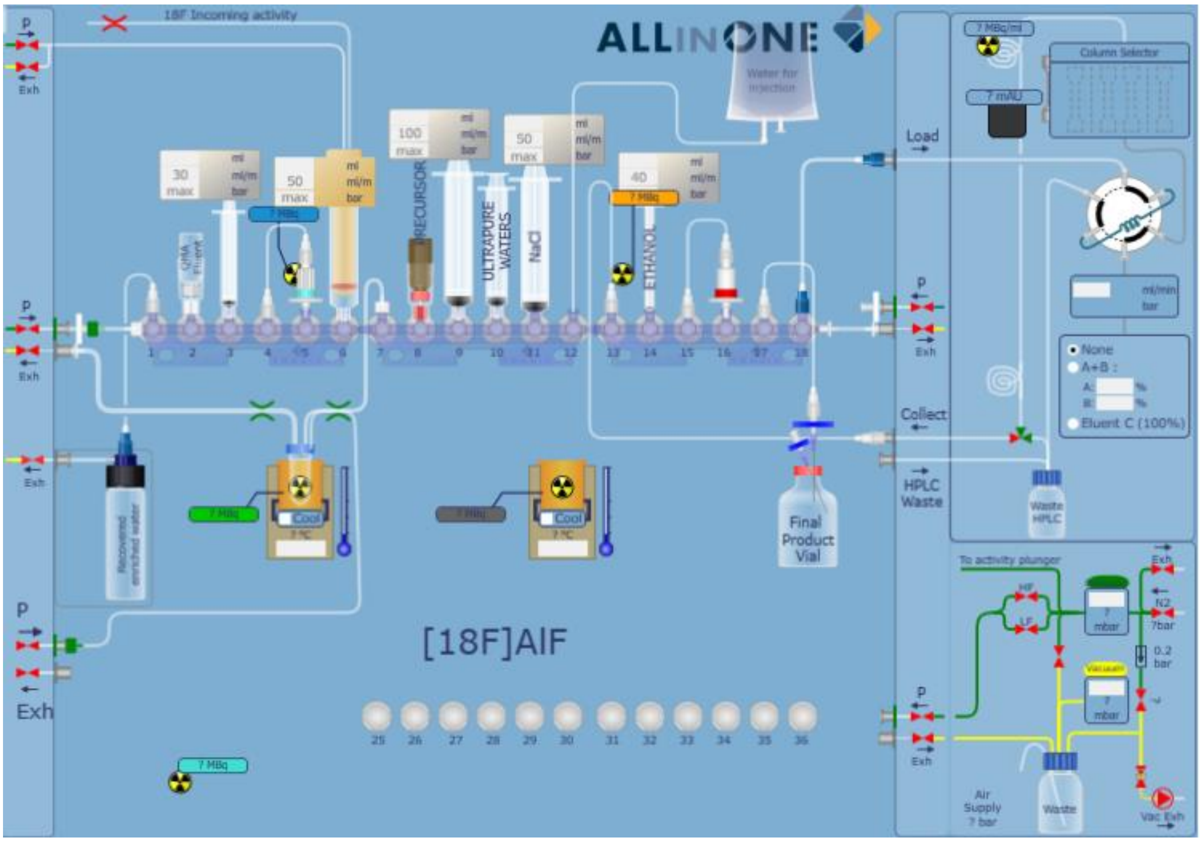

Figure S73: C***onfiguration of Trasis All-in-One automated system. Main reagent positions are indicated:*** **P2: 0.1M sodium acetate buffer pH=4 (300 µL); P5: QMA Sep-Pak cartridge; P8: precursor in 2mM solution (350 µL of EtOH and 100 µL of 0.1M sodium acetate buffer pH=4); P10: ultrapure water (10 mL); P11: Saline physiological solution (19 mL); P14: Ethanol (1 mL); P16: C_{18} Sep-Pak cartridge; Reactor: $AlCl_3$ 0.2 mM solution (50 µL).**

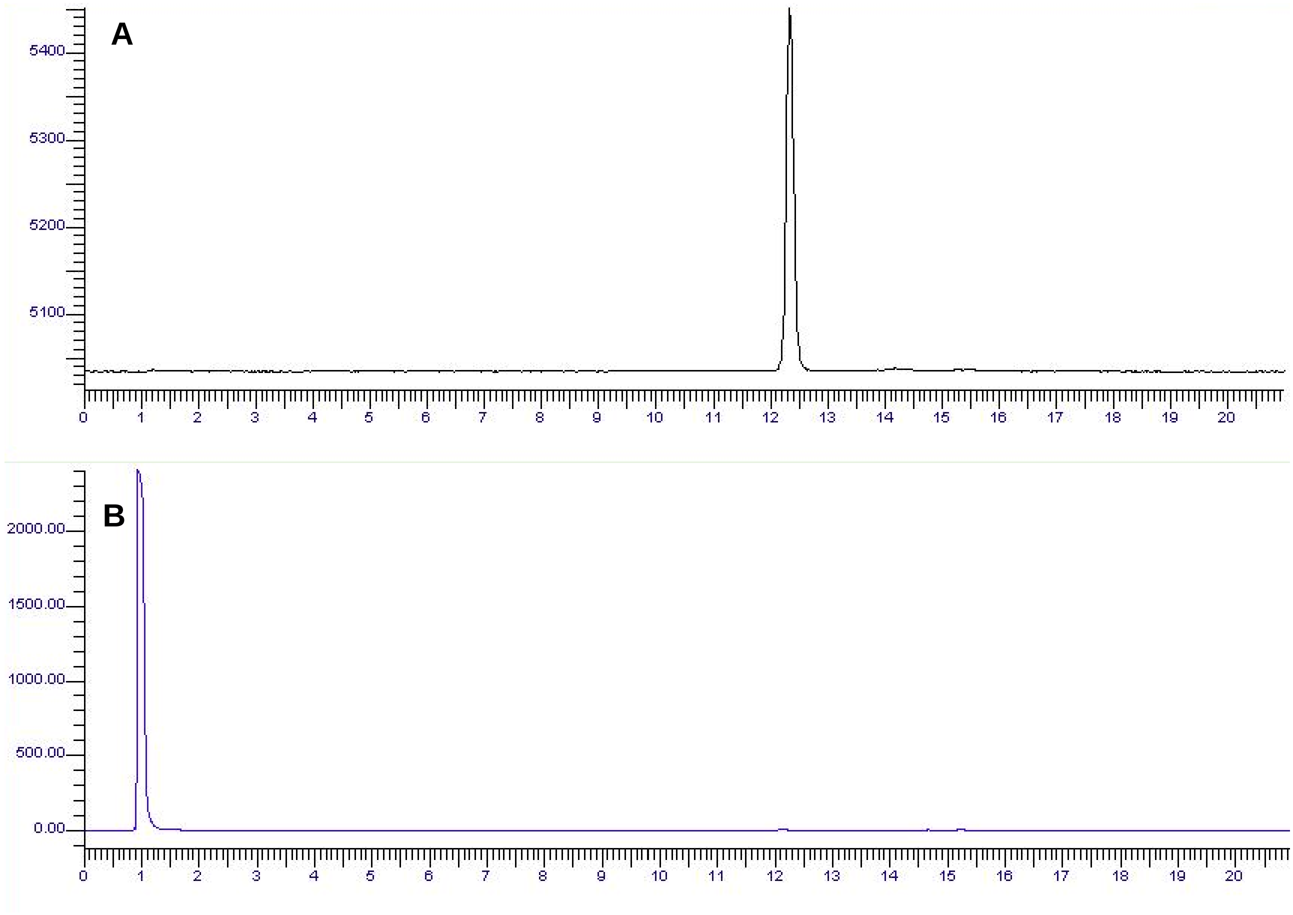

Figure S74: buffer phosphate pH=2.55/ acetonitrile isocratic 77:23 for 2 min, gradient from 77:23 to 70:30 in 12 min, gradient from 70:30 to 40:60 in 3 min, isocratic 40:60 for 4 min; 1.3 ml/min, 225 nm, A) Radiochemical detector, B) UV detector. Rt= 12.1 min
